# Alternate aerosol and systemic immunisation with a recombinant viral vector for tuberculosis, MVA85A: A phase I randomised controlled trial

**DOI:** 10.1371/journal.pmed.1002790

**Published:** 2019-04-30

**Authors:** Zita-Rose Manjaly Thomas, Iman Satti, Julia L. Marshall, Stephanie A. Harris, Raquel Lopez Ramon, Ali Hamidi, Alice Minhinnick, Michael Riste, Lisa Stockdale, Alison M. Lawrie, Samantha Vermaak, Morven Wilkie, Henry Bettinson, Helen McShane

**Affiliations:** 1 Jenner Institute, Nuffield Department of Clinical Medicine, University of Oxford, Oxford, United Kingdom; 2 Oxford Centre for Respiratory Medicine, Nuffield Department of Clinical Medicine, University of Oxford, Oxford, United Kingdom; John Hopkins University, UNITED STATES

## Abstract

**Background:**

There is an urgent need for an effective tuberculosis (TB) vaccine. Heterologous prime–boost regimens induce potent cellular immunity. MVA85A is a candidate TB vaccine. This phase I clinical trial was designed to evaluate whether alternating aerosol and intradermal vaccination routes would boost cellular immunity to the *Mycobacterium tuberculosis* antigen 85A (Ag85A).

**Methods and findings:**

Between December 2013 and January 2016, 36 bacille Calmette-Guérin–vaccinated, healthy UK adults were randomised equally between 3 groups to receive 2 MVA85A vaccinations 1 month apart using either heterologous (Group 1, aerosol–intradermal; Group 2, intradermal–aerosol) or homologous (Group 3, intradermal–intradermal) immunisation. Bronchoscopy and bronchoalveolar lavage (BAL) were performed 7 days post-vaccination. Adverse events (AEs) and peripheral blood were collected for 6 months post-vaccination. The laboratory and bronchoscopy teams were blinded to treatment allocation. One participant was withdrawn and was replaced. Participants were aged 21–42 years, and 28/37 were female. In a per protocol analysis, aerosol delivery of MVA85A as a priming immunisation was well tolerated and highly immunogenic. Most AEs were mild local injection site reactions following intradermal vaccination. Transient systemic AEs occurred following vaccination by both routes and were most frequently mild. All respiratory AEs following primary aerosol MVA85A (Group 1) were mild. Boosting an intradermal MVA85A prime with an aerosolised MVA85A boost 1 month later (Group 2) resulted in transient moderate/severe respiratory and systemic AEs. There were no serious adverse events and no bronchoscopy-related complications. Only the intradermal–aerosol vaccination regimen (Group 2) resulted in modest, significant boosting of the cell-mediated immune response to Ag85A (*p* = 0.027; 95% CI: 28 to 630 spot forming cells per 1 × 10^6^ peripheral blood mononuclear cells). All 3 regimens induced systemic cellular immune responses to the modified vaccinia virus Ankara (MVA) vector. Serum antibodies to Ag85A and MVA were only induced after intradermal vaccination. Aerosolised MVA85A induced significantly higher levels of Ag85A lung mucosal CD4+ and CD8+ T cell cytokines compared to intradermal vaccination. Boosting with aerosol-inhaled MVA85A enhanced the intradermal primed responses in Group 2. The magnitude of BAL MVA-specific CD4+ T cell responses was lower than the Ag85A-specific responses. A limitation of the study is that while the intradermal–aerosol regimen induced the most potent cellular Ag85A immune responses, we did not boost the last 3 participants in this group because of the AE profile. Timing of bronchoscopies aimed to capture peak mucosal response; however, peak responses may have occurred outside of this time frame.

**Conclusions:**

To our knowledge, this is the first human randomised clinical trial to explore heterologous prime–boost regimes using aerosol and systemic routes of administration of a virally vectored vaccine. In this trial, the aerosol prime–intradermal boost regime was well tolerated, but intradermal prime–aerosol boost resulted in transient but significant respiratory AEs. Aerosol vaccination induced potent cellular Ag85A-specific mucosal and systemic immune responses. Whilst the implications of inducing potent mucosal and systemic immunity for protection are unclear, these findings are of relevance for the development of aerosolised vaccines for TB and other respiratory and mucosal pathogens.

**Trial registration:**

ClinicalTrials.gov NCT01954563.

## Abstract 2

In a phase I randomized controlled trial, Helen McShane and colleagues investigate different combinations of intradermal and aerosol prime and boost vaccination for the MVA85A candidate tuberculosis vaccine for safety and ability to stimulate an immune response.

## Author summary

**Why was this study done?:**

- A vaccine for tuberculosis (TB) that is more effective than the bacille Calmette-Guérin (BCG) vaccine is urgently needed.
- Vaccines are usually given as injections into muscle or skin. However, giving a TB vaccine as an inhaler might be a better way of stimulating a protective immune response in the lungs, the point of entry for TB into the body.
- This study was designed to test whether alternating inhaled and injected routes with the same vaccine would stimulate a strong immune response.

**What did the researchers do and find?:**

- This was a phase I blinded randomised controlled clinical trial of 36 BCG-vaccinated, healthy UK adults to receive 2 vaccinations with MVA85A, a test TB vaccine, 28 days apart. Participants received 2 injections into the skin, or 1 injection in the skin and 1 inhaled vaccination. We looked at the immune response induced in the blood and also in the lungs.
- In general the vaccines were well tolerated. Participants who received an injection in the skin and then an inhaled vaccine had some chest symptoms, which all resolved without any treatment.
- The vaccine induced strong immune responses in all groups, but these were slightly stronger in the group that received an injection in the skin and then an inhaled vaccine.

**What do these findings mean?:**

- This study demonstrated that giving a TB vaccine by inhaler was well tolerated when that vaccine was a virus that was made to express a TB protein and when the first dose of vaccine was given by aerosol.
- However, giving the first dose of vaccine as an injection and the second dose as an inhaler 1 month later was not well tolerated, and this particular regimen will not be developed further.
- Inhaled vaccines stimulate immune responses in the blood and the lungs.

## Introduction

Tuberculosis (TB) is the leading global cause of death from a single infectious pathogen, causing 10 million new cases and an estimated 1.6 million deaths in 2017 [1].

The only licensed vaccine, bacille Calmette-Guérin (BCG), is effective at protecting against disseminated disease, but the protection conferred against pulmonary TB in adults is highly variable [2–5]. A more effective vaccine that provides universal protection against pulmonary TB is urgently needed. Recent results from 2 phase II efficacy trials are encouraging. First, revaccination with intradermal BCG led to a 45.4% reduction of sustained *Mycobacterium tuberculosis (M*.*tb)* infection compared to placebo, as measured by Quantiferon conversion [6]. Furthermore, a subunit protein/adjuvant candidate vaccine, M72/AS01e, demonstrated 54% efficacy against TB disease in *M*.*tb* latently infected individuals [7]. These results demonstrate proof of concept in humans. However, further research and development into the most effective vaccination strategy is still needed.

Vaccine delivery via the mucosal route may improve protective immunity. The lungs are the primary route of entry of the causative organism, *M*.*tb*, via inhalation of aerosolised droplets containing infectious bacilli [8,9]. Adults with smear positive pulmonary TB are the main source of transmission. An effective vaccine delivered by aerosol may induce more potent local, vaccine-induced immunity at the site of future *M*.*tb* exposure. Furthermore, an aerosolised TB vaccine would also offer practical advantages including needle-free delivery.

Preclinical animal studies and clinical trials have provided proof of concept for this aerosol vaccination approach [10–16]. One candidate TB vaccine, modified vaccinia virus Ankara (MVA) expressing the mycobacterial antigen 85A (MVA85A), boosted pre-existing BCG-induced immune responses when delivered intradermally in UK adults [17]. However, the immunogenicity was much weaker in South African infants, and no significant improvement in efficacy over BCG alone was seen in a phase IIb efficacy trial [18]. Aerosol delivery may be one way of enhancing immunogenicity, particularly at the site of *M*.*tb* exposure. The safety and feasibility of delivering MVA85A by aerosol has previously been demonstrated in a phase I clinical trial [19]. In this earlier trial, MVA85A delivered by aerosol was well tolerated and highly immunogenic, and induced significantly less systemic humoral anti-vector immunity than intradermal administration [19]. This finding is of interest as anti-vector immunity can limit use and re-use of recombinant viral vectors.

We therefore conducted a second phase I experimental medicine clinical trial to evaluate the safety and immunogenicity of sequential homologous immunisations with MVA85A using both the aerosol and intradermal routes of delivery, with the hypothesis that this vaccination strategy may enhance the immune response to the insert antigen 85A (Ag85A) and minimise anti-vector immunity.

## Methods

### Study design

This phase I randomised, blinded clinical trial was registered with ClinicalTrials.gov (NCT01954563) and was conducted in accordance with good clinical practice. Ethical (NRES South Central-Oxford A, 13/SC/0329), Medicines and Healthcare products Regulatory Agency, National Health Service Research and Development, and University of Oxford GMO approvals for the study were obtained. The University of Oxford was the sponsor. The trial was undertaken at the Centre for Clinical Vaccinology and Tropical Medicine, University of Oxford, and at Oxford University Hospitals NHS Foundation Trust between December 2013 and January 2016.

Thirty-six healthy, BCG-vaccinated UK adults aged 21 to 42 years were randomised equally between 3 groups to receive 2 MVA85A vaccinations 1 month apart using either heterologous (Group 1, aerosol–intradermal; Group 2, intradermal–aerosol) or homologous (Group 3, intradermal–intradermal) immunisation.

An independent local safety committee was established, chaired by Professor Brian Angus, Fellow of the Royal College of Physicians, Director of the Oxford Centre for Clinical Tropical Medicine, and Infectious Diseases/General Medicine Consultant at the Oxford University Hospitals NHS Foundation Trust. The committee was informed of any untoward adverse event (AE) profile that differed from past experience with MVA85A.

Written informed consent was obtained from all volunteers prior to screening. Participants were clinically evaluated and deemed eligible if they were healthy, with normal spirometry and normal peripheral oxygen saturations; normal baseline haematology, coagulation, and biochemistry blood results; a normal chest radiograph; and negative serological testing for hepatitis B, hepatitis C, and HIV. Latent *M*.*tb* infection in participants was excluded by a negative ex vivo enzyme-linked immunospot (ELISpot) response to early secreted antigenic target–6 (ESAT-6) and culture filtrate protein–10 (CFP-10) peptides. Current smokers, those using nasally instilled or inhaled drugs, and anyone with a history of respiratory disease were excluded. Following an amendment to restart enrolment into Group 2, volunteers with any history of atopy were also excluded from enrolment into the trial. A fourth regime of aerosol prime–aerosol boost was not included in the trial design because of concerns about the feasibility of enrolment of a larger study.

Group 1 received MVA85A administered by aerosol, and a second intradermal dose of MVA85A 1 month later. Group 2 received MVA85A administered intradermally, and then a second dose of aerosolised MVA85A 1 month later. Group 3 received both doses of MVA85A intradermally, with 1 month between immunisations. To maintain blinding, the vaccine was paired with a saline placebo so that both an injection and aerosol inhalation were administered to all volunteers at each vaccination time point. Blood was taken at every scheduled trial visit (screening, day 0 [D0], D2, D7, D14, D28, D35, D42, D84, and D168). Bronchoscopy and bronchoalveolar lavage (BAL) were performed 7 days after each vaccination (D7 and D35).

Three volunteers in Group 2 received placebo instead of MVA85A on D28 following a safety review.

### Sample size and study endpoints

The primary objective of the study was to investigate the safety of a 5 × 10^7^ pfu dose of MVA85A administered by aerosol, compared to the same dose administered intradermally, through actively and passively collected data on AEs. We also aimed to determine the tolerability of the MVA85A boosting by heterologous route regimes evaluated in this clinical trial. We hypothesised that there would be no substantial difference in severity of AEs between the 3 groups in this trial, or for the different routes of vaccine delivery. These endpoints are primarily descriptive, designed to establish any substantial or clinically significant safety differences between groups. As a proof-of-concept phase I safety study, the sample size of 12 participants per group was determined based on the feasibility of the number to recruit, screen, enrol, and follow up in practical terms, whilst also allowing the determination of any substantial and clinically significant differences in safety and cell-mediated immunity between the 3 groups [19] and was not designed to produce statistically powered results.

Secondary objectives were characterisation of the mucosal and systemic immunogenicity of viral vector (MVA) and insert (Ag85A) by comprehensive characterisation of humoral and cellular immune responses and evaluation of the functional relevance of anti-vector immunity induced by aerosol and systemic immunisation in MVA85A prime followed by MVA85A boost administered 4 weeks later. Specifically, the primary immunological endpoint (ELISpot response to Ag85A peptide stimulation) was designed to test the hypothesis that a heterologous prime–boost regime (Group 1 or Group 2) would result in a stronger and more sustained cellular immune response than intradermal vaccination alone (Group 3). A 2-fold increase was considered immunologically meaningful. The secondary endpoint (ELISpot response to MVA stimulation) tests the hypothesis that mucosal immunisation with MVA85A does not result in induction of systemic anti-vector immunity by comparing the mucosal and systemic immunogenicity of viral vector (MVA) between Group 3 (intradermal vaccinations only) and the heterologous regimes Group 1 and Group 2. Extrapolating effect size and variability from the completed initial aerosol vaccine safety study allowed us to determine that 12 volunteers were needed to have 80% power to detect an immunologically meaningful 2-fold difference in the summed peptide pools, and 8 volunteers to achieve the same for the single peptide pool of 85A [19].

### Randomisation and blinding

Participants were randomised with variable block randomisation by sequentially numbered opaque sealed envelopes, prepared by an independent trial statistician, which were opened at enrolment. Following an interim amendment to pause enrolment into Group 2 for safety review, envelopes with that group number were omitted whilst enrolment into the other 2 groups continued in the randomised sequence. Following the safety review and consultation with the chair of the local safety committee, enrolment into Group 2 was restarted with the implementation of more stringent exclusion criteria and a step-wise approach to volunteer recruitment into this group: 1 volunteer was to be enrolled into this group, with the subsequent volunteer being enrolled only after completion of both vaccinations and favourable safety review in the previous volunteer. Once the first 2 participants were enrolled this way with a favourable safety review, subsequent volunteers were then enrolled. Participants, the bronchoscopist, and the senior immunologist remained blinded throughout the trial. As this was a phase I experimental medicine study, the trial clinician was not blinded for logistical and safety reasons because the trial clinician was overseeing vaccination and monitoring safety data.

### Vaccination

MVA85A was manufactured under good manufacturing practice conditions by IDT Biologika. The dose of MVA85A was 5 × 10^7^ pfu, administered in a volume of 135 μl for intradermal injection and diluted with 0.9% sodium chloride to a volume of 1 ml for aerosol vaccine delivery using the Omron MicroAir U22 ultrasonic mesh nebuliser (Omron Healthcare).

The last 3 volunteers in Group 2 received placebo by both intradermal and aerosol routes on D28 as a safety measure. Their immunogenicity data have been excluded from the immunological analysis apart from their D7 BAL responses.

### Bronchoscopy and BAL

Fibre-optic flexible bronchoscopy procedures were performed on all volunteers 7 days after each vaccination at Oxford University Hospitals NHS Foundation Trust. Written consent was obtained. Intravenous sedation (midazolam up to 3 mg and fentanyl up to 100 μg), local anaesthesia spray above the cords (by metered dose valve, xylocaine delivering approximately 0.02 mg in 0.1 ml/spray), and lidocaine 2% (2 ml) above and below the cords were administered. Macroscopic appearances were examined and photographs taken before BAL was collected from the right middle lobe using 100 ml of normal saline. No endobronchial biopsies were taken.

Vital signs were monitored throughout the procedure, and volunteers observed for 90 minutes after the procedure.

### Clinical AE monitoring

Safety was assessed by the frequency and severity of any AEs during the trial period. Expected local skin AEs (pain, erythema, scaling, itch, warmth, and swelling), respiratory AEs (cough, sore throat, wheeze, dyspnoea, sputum production, haemoptysis, and chest pain), and systemic AEs (fever, feverishness, fatigue, malaise, headache, myalgia, arthralgia, and nausea) were solicited from participants using a diary card for 14 days after each vaccination and reviewed at every clinic visit. Routine biochemical and haematological parameters were measured on D7 and D35. Following a protocol amendment, additional safety blood tests on D2 and D30 were introduced. Volunteers were trained in the use of a handheld spirometer (Micro Spirometer, CareFusion) for daily home measurement of forced expiratory volume in 1 second (FEV_1_) and forced vital capacity (FVC) daily for 14 days after each vaccination. Peripheral oxygen saturation measurements were taken at all clinic visits.

### Ex vivo interferon-γ (IFNγ) ELISpot

The ex vivo IFNγ ELISpot assay was performed on fresh peripheral blood mononuclear cells (PBMCs) separated from whole blood (WB) as previously described [20]. Samples were collected at baseline and on D7, D14, D28, D35, D42, D84, and D168 of the trial. PBMCs (3 × 10^5^/well) were stimulated with a single pool of 15mer peptides spanning the length of Ag85A, MVA CD4+ T cell and CD8+ T cell epitopes, ESAT-6 and CFP-10 peptides (Peptide Protein Research), and staphylococcal enterotoxin B (SEB, positive control; Sigma). IFNγ production from PBMCs in unstimulated wells was taken as background. Presented results are spot forming cells (SFC) per 1 × 10^6^ PBMCs, calculated by subtracting background responses from the mean responses of triplicate wells and correcting for number of cells in the well.

### BAL separation and stimulation

BAL was transported to the laboratory at ambient room temperature and processed within 2 hours. BAL samples were centrifuged, supernatant cryopreserved, and cell pellets pooled and counted. Then 1 × 10^6^ BAL cells were stimulated with Ag85A, MVA CD4, and MVA CD8 peptides (2 μg/ml). SEB (5 μg/ml) was used as positive control for the assay, while unstimulated cells were used as negative control. Co-stimulatory antibodies, anti-CD28 and anti-CD49d (BD) at 1 μg/ml each, were added to all samples. After a 2-hour incubation in 5% CO_2_ at 37 °C, 3 μg/ml of Brefeldin A (Sigma-Aldrich) was added, and the samples were incubated overnight and stained the following morning.

### WB stimulation

Fresh WB was stimulated, lysed, fixed, and cryopreserved as previously described [19]. Samples were collected at baseline and on D7, D14, D28, D35, D42, and D168 of the trial. WB samples were stimulated with either Ag85A peptide pool or a pool of MVA CD4 and CD8 peptides (2 μg/ml each) or SEB as a positive control in the presence of co-stimulatory antibodies, anti-CD28 and anti-CD49d (BD). Unstimulated samples were included as negative controls. Red blood cells were lysed and samples cryopreserved.

### Intracellular cytokine staining (ICS)

ICS was performed as previously described [19]. Fixed WB cells were thawed, permeabilised, and stained for CD3-AF700 (eBioscience), CD4-Pacific Blue (BioLegend), CD8-APC/H7 (BD), CD14 and CD19 on ECD (Beckman Coulter), IFNγ-PECY7 (eBioscience), TNFα-AF-647 (BioLegend), IL2-PE (Beckman Coulter), and IL17-AF488 (BioLegend). BAL cells were stained fresh using the same antibody panel as the WB.

Cells were first stained for viability (Live/Dead Stain, Invitrogen) before surface antibody staining for CD4, CD14, and CD19. Next, samples were permeabilised before addition of intracellular cytokine antibodies for CD3, CD8, IFNγ, TNFα, IL2, and IL17.

All stained cells were fixed with 1% paraformaldehyde for immediate acquisition on a BD LSRII flow cytometer. All flow cytometry data were analysed on FlowJo (TreeStar) V8 for BAL data or V9 for WB ICS data.

Gating strategy for WB samples was as follows: Lymphocytes were gated on a forward scatter area (FSC-A) versus side scatter (SSC). Next, duplets were excluded on a forward scatter height (FSC-H) versus FSC-A. Also, CD14+ and CD19+ cells were excluded. For BAL samples, dead cells were also excluded. Within CD3+ lymphocytes, CD4+ and CD8+ subsets were determined, and this was followed by gating on cytokine+ cells (S1 Fig).

Presented are percentages of cytokine positive CD4+ and CD8+ T cells in antigen stimulated samples with background responses in unstimulated cells subtracted. Polyfunctional response analysis was done using Spice and Pestle (http://exon.niaid.nih.gov/spice/).

### Enzyme-linked immunosorbent assay (ELISA)

Insert- and vector-specific antibody responses were assessed by ELISA in serum and BAL fluid samples as previously described [19]. Serum samples were collected at baseline and on D7, D14, D28, D35, D42, and D168. Nunc Immunoplates were coated with 2 ug/ml r85A (Lionex) or 5 × 10^5^ pfu/well non-recombinant MVA (Virus Vector Core Group, Jenner Institute) and incubated overnight for adsorption. Plates were then blocked with casein (Pierce) and diluted serum (1:10 in casein) or neat BAL fluid. Samples were added in triplicate wells and incubated for 2 hours. Bound antibodies were detected using goat anti-human secondary antibody conjugated to alkaline phosphatase (Sigma-Aldrich), and plates were developed by adding substrate. Optical density (OD) was read at 405 nm absorbance. Data presented are OD values with background subtracted. Acceptable threshold for background was an OD value of <0.15 at 405 nm absorbance in blank wells.

### Statistical analysis

All statistical analysis was performed using GraphPad Prism (version 7 and version 8) and R version 3.5.2. Clinical AEs were summarised by frequency and severity of AEs by group. Non-parametric tests were applied as the data were not normally distributed on normality testing. Within each group, paired data were analysed using Wilcoxon matched pairs test for comparison of 2 sets of paired data or Friedman`s test for comparison of 3 or more paired datasets. To compare unpaired datasets between groups, the Mann–Whitney test was used for comparison of 2 datasets and the Kruskal–Wallis test was used for comparison of 3 datasets. Dunn’s multiple comparison test was used for post hoc corrections where applicable. Significant differences in medians between time points or groups are presented along with their *p*-value. The significance level was assumed to be *p* < 0.05 with a 95% CI. *p*-values have been rounded to the 3^rd^ decimal place. An area under the curve (AUC) analysis of deviation from baseline was used to compare the overall spirometry values between the 3 study groups in the time period following vaccination (negative peak area only) as well as for the IFNγ ELISpot responses over the trial period. This analysis made use of data collected at all time points. The AUC was compared between the 3 groups using the Kruskal–Wallis test. The subsequent pairwise comparisons were done using Mood’s median test.

For the final 3 volunteers in Group 2, data following the intradermal MVA85A prime was included in the safety analysis but not the data following the saline aerosol placebo at D28. Data from these volunteers were not included in the ELISpot, ELISA, or WB ICS analysis at any time point. However, data from these volunteers were included in the D7 BAL analysis.

## Results

### Participants

**Fig 1:**
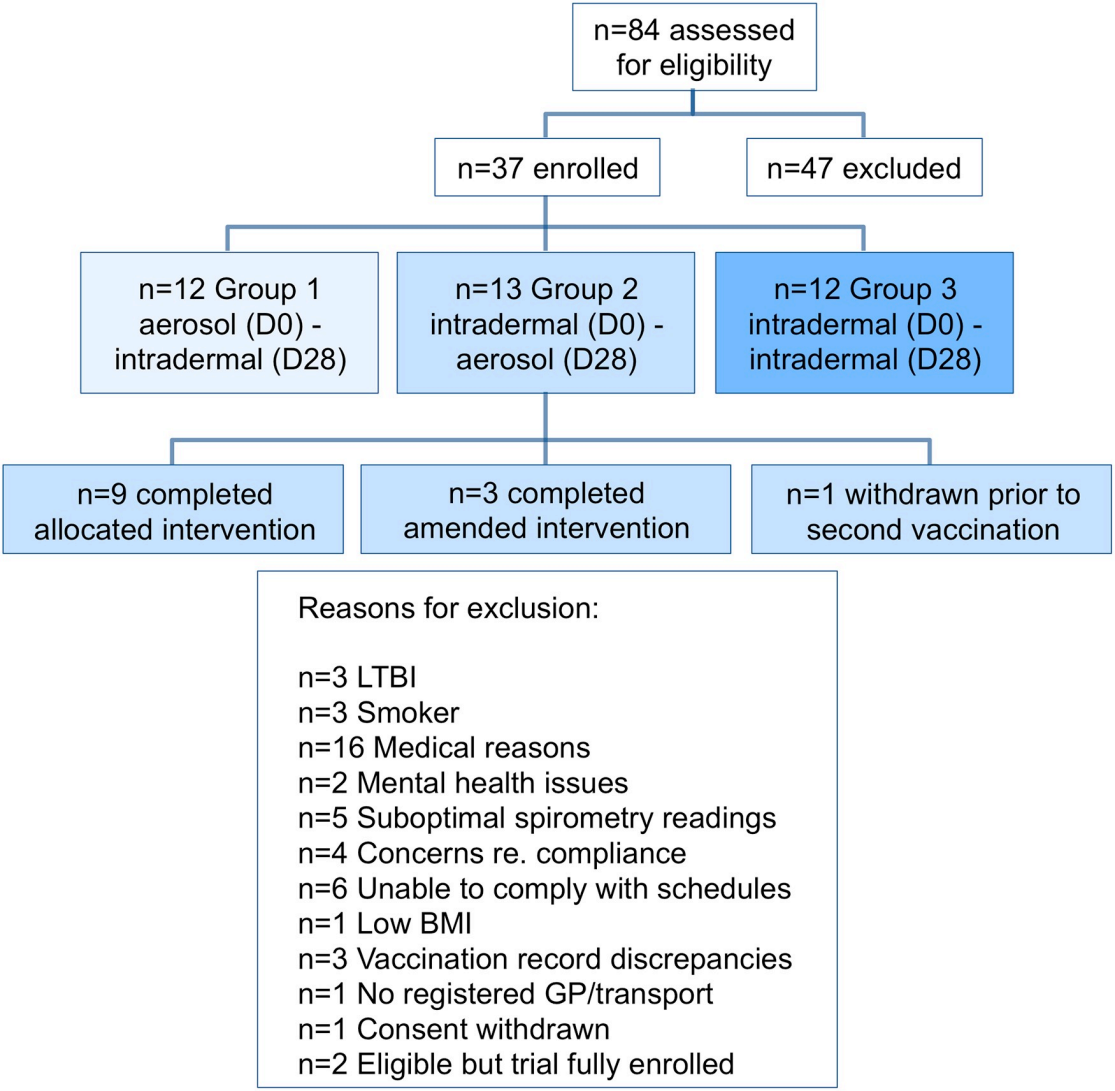
CONSORT diagram. GP, general practitioner; LTBI, latent tuberculosis infection.

Enrolment details are summarised in the CONSORT diagram (Fig 1). In total, 84 volunteers were screened for eligibility. Between December 4, 2013, and August 26, 2015, 37 BCG-vaccinated, healthy adults (aged 21–42 years old) were enrolled into the trial; 36 completed the trial. One participant was withdrawn prior to their D28 vaccination because of an inability to complete the trial that was not evident at enrolment.

There were no significant differences in baseline demographics between the groups apart from sex. Group 3 had significantly more male participants than either Group 1 or 2 (S1 Table).

### Clinical AEs

**Fig 2:**
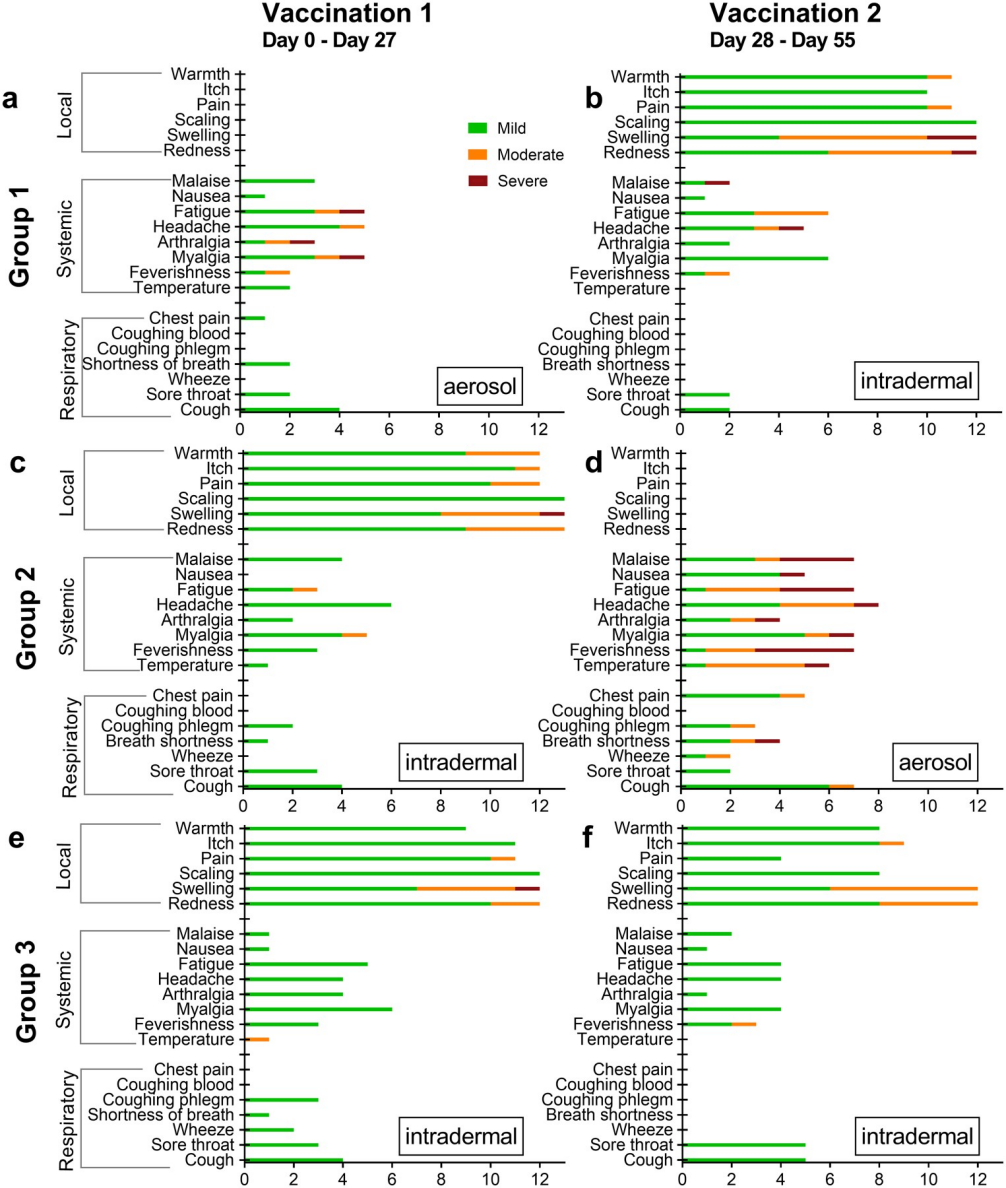
Frequency of related solicited adverse events. Frequency of related solicited adverse events experienced at least once; recorded at peak severity grading in the 28 days following each vaccination. Red = severe, yellow = moderate, gree*n* = mild. *X* axis = number of volunteers, *Y* axis = symptoms. (a and b) Group 1, *n* = 12; (c and d) Group 2, vaccination 1, *n* = 13, vaccination 2, *n* = 9; (e and f) Group 3, *n* = 12.

There were no serious adverse events (SAEs) during this trial. The numbers of participants experiencing AEs are shown in Fig 2 and in S2 Table. The most frequently occurring AEs were local reactions at the injection site after intradermal MVA85A vaccination in all groups (D28 for Group 1, D0 for Group 2, and both D0 and D28 for Group 3; 52% of all AEs; Fig 2). The majority of these local reactions to intradermal vaccination were mild in severity (81%).

Systemic AEs occurred throughout the trial period following vaccination by both routes (Fig 2; S2 Table). Solicited systemic AEs most frequently occurred following the aerosol boost vaccination in Group 2, comprising 66% of all systemic AEs after an aerosol vaccination (Group 1, D0 aerosol prime; Group 2, D28 aerosol boost). Of all the systemic AEs occurring in Group 2 after aerosol boost vaccination, 59% were graded as moderate or severe (S3 Table).

All solicited respiratory AEs following the D0 vaccination were graded as mild regardless of vaccination route (Group 1, aerosol; Groups 2 and 3, intradermal). Following the D28 vaccinations (Group 1 and 3, intradermal; Group 2, aerosol), 14% of all respiratory AEs were graded as moderate and 3% as severe, and these occurred exclusively in Group 2.

We reviewed the clustering of AEs by group, and compared the number of AEs individuals experienced across the groups (S4 Table). The only significant difference was that participants in Group 3, who received 2 intradermal injections, were more likely to experience local AEs than those in Groups 1 or 2. There was no difference in systemic or respiratory AEs by group.

Overall, solicited systemic AEs were more severe, and solicited respiratory AEs were more frequent and more severe, following the D28 aerosol boost vaccination (Group 2) compared to either of the other boost vaccinations.

There were no clinically significant differences (defined as a >15% decrease from the individual’s baseline) in FEV_1_ or FVC compared to baseline after the first vaccination in any group (S2 Fig; *p* = 0.808). Following the D28 vaccination, volunteers in Group 2 demonstrated a clinically significant drop in FEV_1_ and FVC in the 7 days following their aerosol boost vaccination, which was not observed in the other 2 groups during that period (S2 Fig; difference in median AUC: Group 1 versus Group 2 FEV_1_ 0.47 [95% CI: 0.29 to 0.66], *p* = 0.014, FVC 0.53 [95% CI: 0.39 to 0.69], *p* = 0.014; and Group 2 versus Group 3 FEV_1_ −0.47 [95% CI: −0.66 to −0.26], *p* = 0.014, FVC −0.77 [95% CI: −1.13 to −0.32], *p* = 0.014). In Group 2 volunteers, the greatest median reduction in FEV_1_ was a 20% decrease from baseline (IQR 25% to 13%) and in FVC was 18% (IQR 25% to 10%) on the day following the boost vaccination, which returned to normal within 72 hours in all volunteers. Peripheral oxygen saturation obtained at clinic visits was always within the normal range, except as anticipated during bronchoscopy, when volunteers were sedated, during which time oxygen was provided as is routine clinical practice.

There were no procedural complications during the bronchoscopies, and macroscopic appearances in all groups were reported as normal, with occasional mild erythema and other incidental findings that were not felt to be clinically significant by the bronchoscopist.

Enrolment into Group 2 was temporarily paused after 2 out of 4 volunteers experienced transient mild to moderate respiratory AEs, severe systemic AEs, and a transient reduction in pulmonary function following their D28 aerosol boost vaccination. Both volunteers made a full recovery within 72 hours following vaccination and proceeded with their scheduled bronchoscopy on D35. Macroscopic appearances at bronchoscopy were unremarkable. Review of clinical and immunology data collected for these volunteers revealed no significant differences compared to other enrolled volunteers, apart from a clinical history of atopy. Despite a protocol amendment and subsequent exclusion of volunteers with any history of atopy, 5 subsequent volunteers displayed similar clinical features following their D28 aerosol boost (totalling 7 out of 9 enrolled volunteers). These AEs were all transient and resolved fully within 6 days. However, in light of the frequency of these AEs, this arm of the trial was discontinued. The protocol was amended, and the remaining 3 volunteers who had already been enrolled into Group 2 (and had received their first intradermal MVA85A vaccination) received saline placebo by both aerosol and intradermal injection on D28.

### Ex vivo IFNγ ELISpot

**Fig 3:**
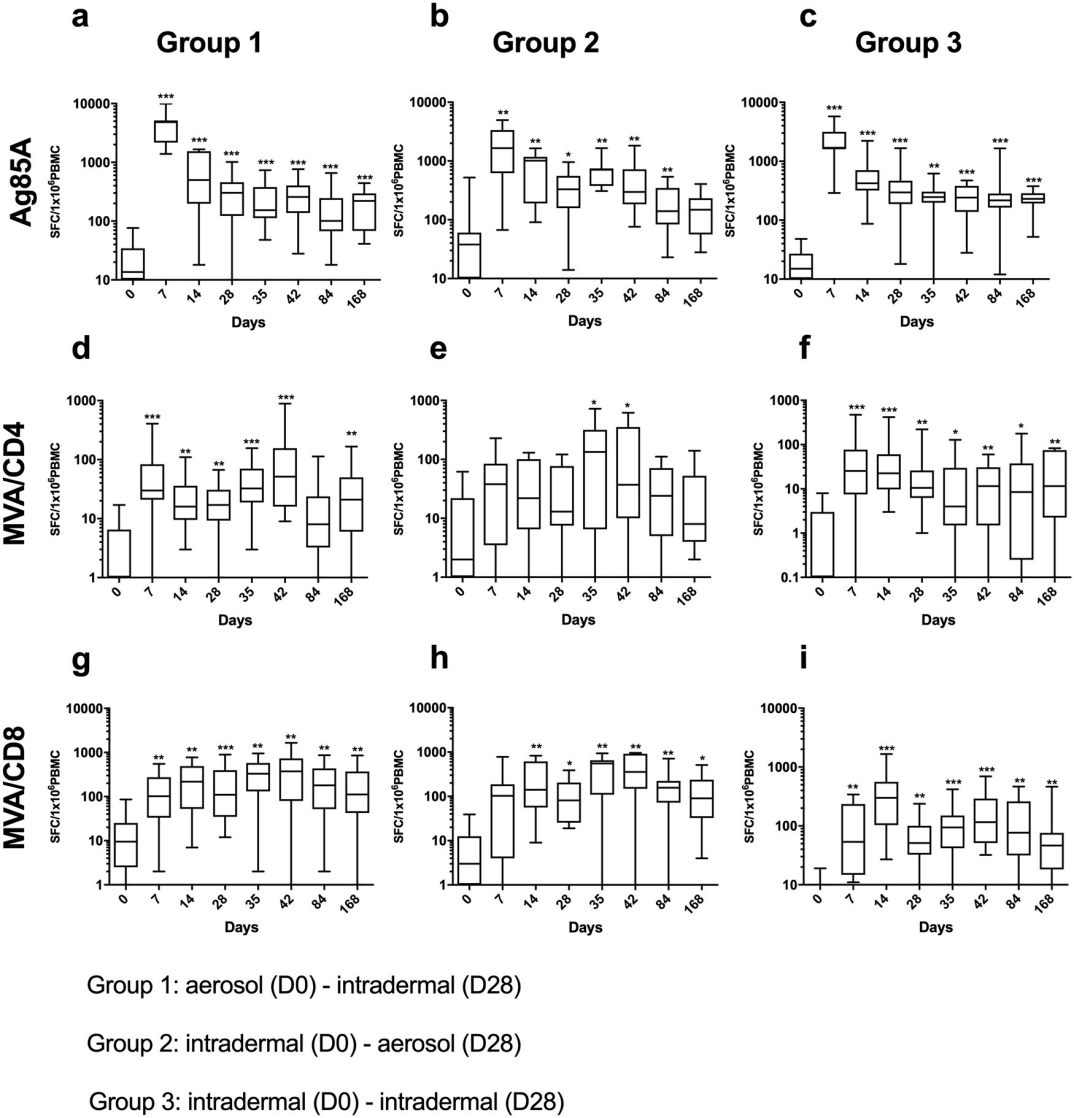
Frequency of antigen-specific IFNγ secreting cells to Ag85A, MVA CD4 peptides, and MVA CD8 peptides by group. Frequency of antigen-specific IFNγ secreting cells to (a–c) Ag85A peptides (top row), (d–f) MVA CD4 peptides (middle row), and (g–i) MVA CD8 peptides (bottom row) in Group 1 (left column), Group 2 (middle column), and Group 3 (right column). *X* axis: time points in days, *Y* axis: SFC per 1 × 10^6^ PBMCs. Shown are significant differences from baseline (day 0). Non-significant differences (*p* > 0.05) omitted for clarity. *p < 0.05, **p < 0.01, ***p < 0.001. Ag85A, antigen 85A; MVA, modified vaccinia virus Ankara; PBMC, peripheral blood mononuclear cell; SPC, spot forming cells.

There were no significant differences in baseline IFNγ ELISpot responses between the groups to any of the studied antigens (Fig 3; *p* > 0.05).

Median AUC analysis of Ag85A-specific IFNγ ELISpot responses demonstrated no significant differences between groups (*p* = 0.912, 1-way ANOVA [Kruskal–Wallis] adjusted for multiple comparisons, and *p* > 0.05, Mann–Whitney test; S7 Table).

In all 3 groups, Ag85A-specific IFNγ ELISpot responses peaked at D7 following the first vaccination (Fig 3; D7 versus D0: Group 1, *p* = 0.001 [95% CI: 2,057 to 4,980 SFC per 1 × 10^6^ PBMCs]; Group 2, *p* = 0.004 [95% CI: 371 to 4,169 SFC per 1 × 10^6^ PBMCs]; Group 3, *p* = 0.001 [95% CI: 1,606 to 3,619 SFC per 1 × 10^6^ PBMCs]). Responses after an aerosol prime vaccination (Group 1) on D7 were significantly higher than after an intradermal prime (Groups 2 and 3): Group 1 versus Group 2 (*p* = 0.017, 95% CI: 40 to 4,167 SFC per 1 × 10^6^ PBMCs) and Group 3: (*p* = 0.021, 95% CI: 411 to 3,342 SFC per 1 × 10^6^ PBMCs).

The Ag85A-specific responses were significantly higher on D35 than before the boost vaccination on D28 in Group 2 only (Fig 3; D35 versus D28: *p* = 0.027 [95% CI: 28 to 630 SFC per 1 × 10^6^ PBMCs]). These Group 2 responses at D35 following the aerosol boost were significantly higher than those in Groups 1 and 3 at D35 after the intradermal boost (Group 2 versus Group 1: *p* = 0.002 [95% CI: 186 to 609 SFC per 1 × 10^6^ PBMCs]; Group 2 versus Group 3: *p* < 0.001 [95% CI: 125 to 524 SFC per 1 × 10^6^ PBMCs]).

Ag85A-specific responses remained significantly elevated compared to D0 (baseline) in Groups 1 and 3 until D168 (*p* = 0.001 for both Group 1 [95% CI: 59 to 258 SFC per 1 × 10^6^ PBMCs] and Group 3 [95% CI: 170 to 243 SFC per 1 × 10^6^ PBMCs]) and in Group 2 until D84 (*p* = 0.004 [95% CI: 12 to 199 SFC per 1 × 10^6^ PBMCs]). There was no significant difference between the groups at D168 (all *p* > 0.05).

Median AUC analysis of MVA-specific CD4+ and CD8+ T cell IFNγ ELISpot responses demonstrated no significant differences between groups (*p* = 0.280 and *p* = 0.499 for MVA CD4+ and CD8+ T cells, respectively, 1-way ANOVA [Kruskal–Wallis] adjusted for multiple comparisons; *p* > 0.05, Mann–Whitney test; S7 Table). MVA-specific CD4+ T cell responses on D7 were significantly higher than baseline in Group 1 and 3 (*p* = 0.001 for both Group 1 [95% CI: 11 to 86 SFC per 1 × 10^6^ PBMCs] and Group 3 [95% CI: 6 to 78 SFC per 1 × 10^6^ PBMCs]). In Group 2, the MVA-specific CD4+ T cell responses were only significantly higher than baseline at D35 (D35 versus D0: *p* = 0.042 [95% CI: 45 to 414 SFC per 1 × 10^6^ PBMCs]). Following the D28 boost vaccination there was a significant increase in MVA-specific CD4+ T cell responses above the pre-boost D28 responses in Groups 1 and 2 (D42 versus D28: *p* = 0.007 for Group 1 [95% CI: 5 to 102 SFC per 1 × 10^6^ PBMCs] and *p* = 0.039 for Group 2 [95% CI: 2 to 300 SFC per 1 × 10^6^ PBMCs]).

MVA-specific CD8+ T cell responses peaked at D14 in all groups. Responses in Groups 1, 2, and 3 were significantly higher following the D28 vaccination (D42 versus D28: Group 1, *p* = 0.027 [95% CI: 15 to 398 SFC per 1 × 10^6^ PBMCs], Group 2, *p* = 0.008 [95% CI: 97 to 745 SFC per 1 × 10^6^ PBMCs], and Group 3, *p* = 0.009 [95% CI: 27 to 253 SFC per 1 × 10^6^ PBMCs]).

### WB ICS

**Fig 4:**
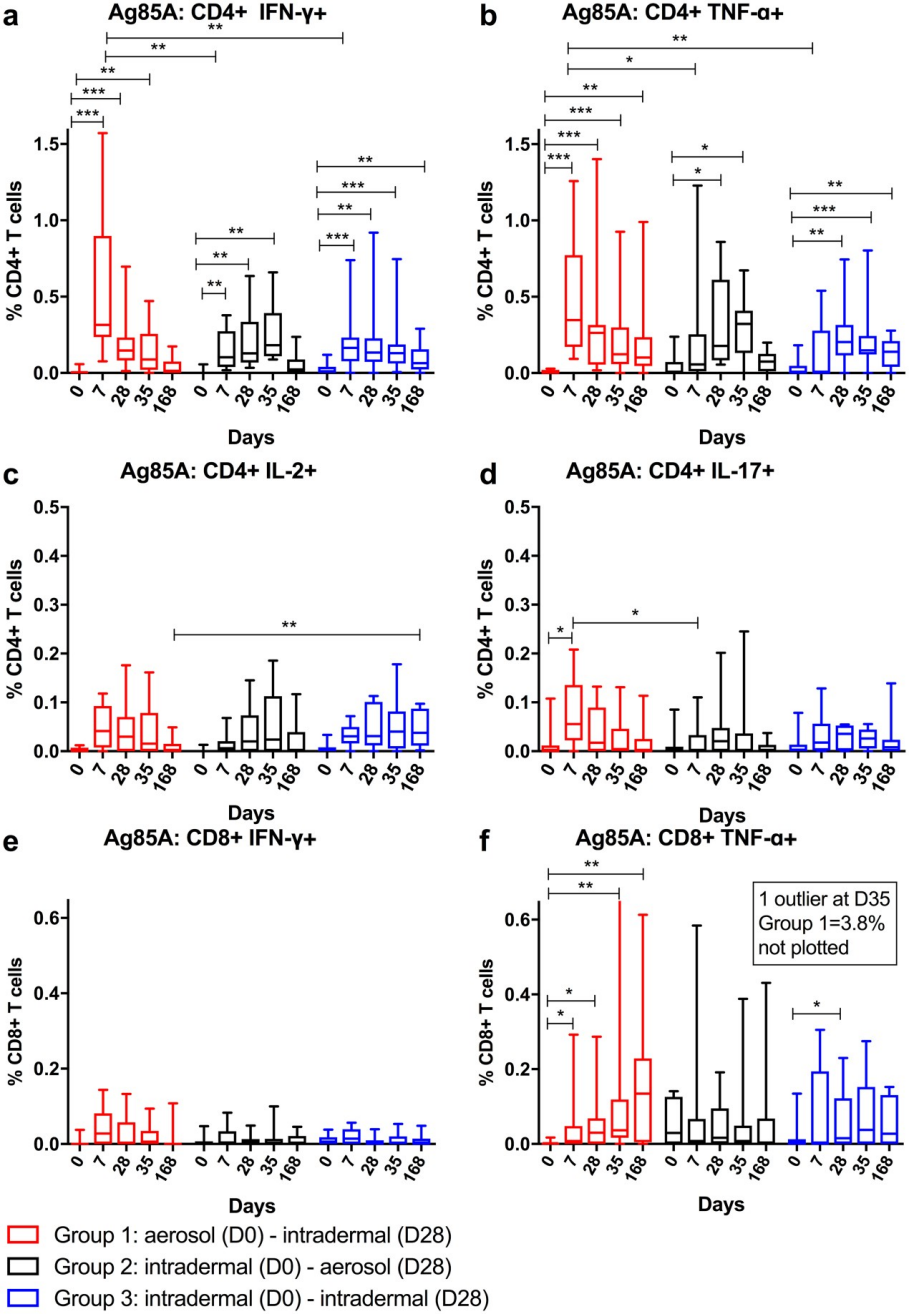
WB ICS Ag85A-specific CD4+ T cell responses. WB ICS Ag85A-specific CD4+ T cells positive for IFNγ (a), TNFα (b), IL2 (c), and IL17 (d) and CD8+ T cells positive for IFNγ (e) and TNFα (f). *X* axis = time points in days, *Y* axis = percent of CD4+ or CD8+ T cells positive for that cytokine. Statistically significant differences from baseline and between groups are shown. Non-significant differences (*p* > 0.05) are omitted for clarity. *p < 0.05, **p < 0.01, ***p < 0.001. Note the different *Y* axis scales. Ag85A, antigen 85A; ICS, intracellular cytokine staining; WB, whole blood.

Intracellular cytokines were measured on freshly stimulated, fixed and frozen WB (Fig 4). Median AUC analysis of Ag85A-specific CD4+ and CD8+ T cell cytokine responses showed no significant differences between groups (S8 and S9 Tables). In all 3 groups, the frequency of Ag85A-specific IFNγ+ CD4+ T cells increased on D7 (D0 versus D7: Group 1, *p* = 0.001; Group 2, *p* = 0.004; Group 3, *p* = 0.001). TNFα+ CD4+ cells increased at D7 for Group 1 (*p* = 0.001) and at D28 for Group 2 and Group 3 (*p* = 0.039 and *p* = 0.002, respectively). The Ag85A-specific IFNγ+ and TNFα+ CD4+ T cell responses were significantly higher on D7 in Group 1 (IFNγ: *p* = 0.009, Group 1 versus Group 2; *p* = 0.008, Group 1 versus Group 3; TNFα: *p* = 0.024, Group 1 versus Group 2; *p* = 0.009, Group 1 versus Group 3). Following the second vaccination there was no boosting in Ag85A-specific CD4+ T cell responses in any of the groups (*p* > 0.05 for all 3 groups), nor was there any difference between the groups on D35 (*p* = 0.191). Ag85A-specific IL2+ and IL17+ CD4+ T cell responses were much lower in frequency than IFNγ+ and TNFα+ responses and were not significantly different from baseline, except for IL17+ responses in Group 1 on D7 (D0 versus D7: *p* = 0.014).

There was no significant increase (compared to D0) in Ag85A-specific IFNγ+ CD8+ T cell responses in any of the groups at any time point (Fig 4). In Group 1, Ag85A-specific TNFα+ CD8+ T cell responses peaked on D7 and remained significantly elevated above baseline at D168 (D0 versus D7: *p* = 0.031; D0 versus D168: *p* = 0.003). There was no boosting of any Ag85A-specific CD8+ T cell responses in any of the groups. Ag85A-specific IL2+ and IL17+ CD8+ T cell responses were not detectable.

**Fig 5:**
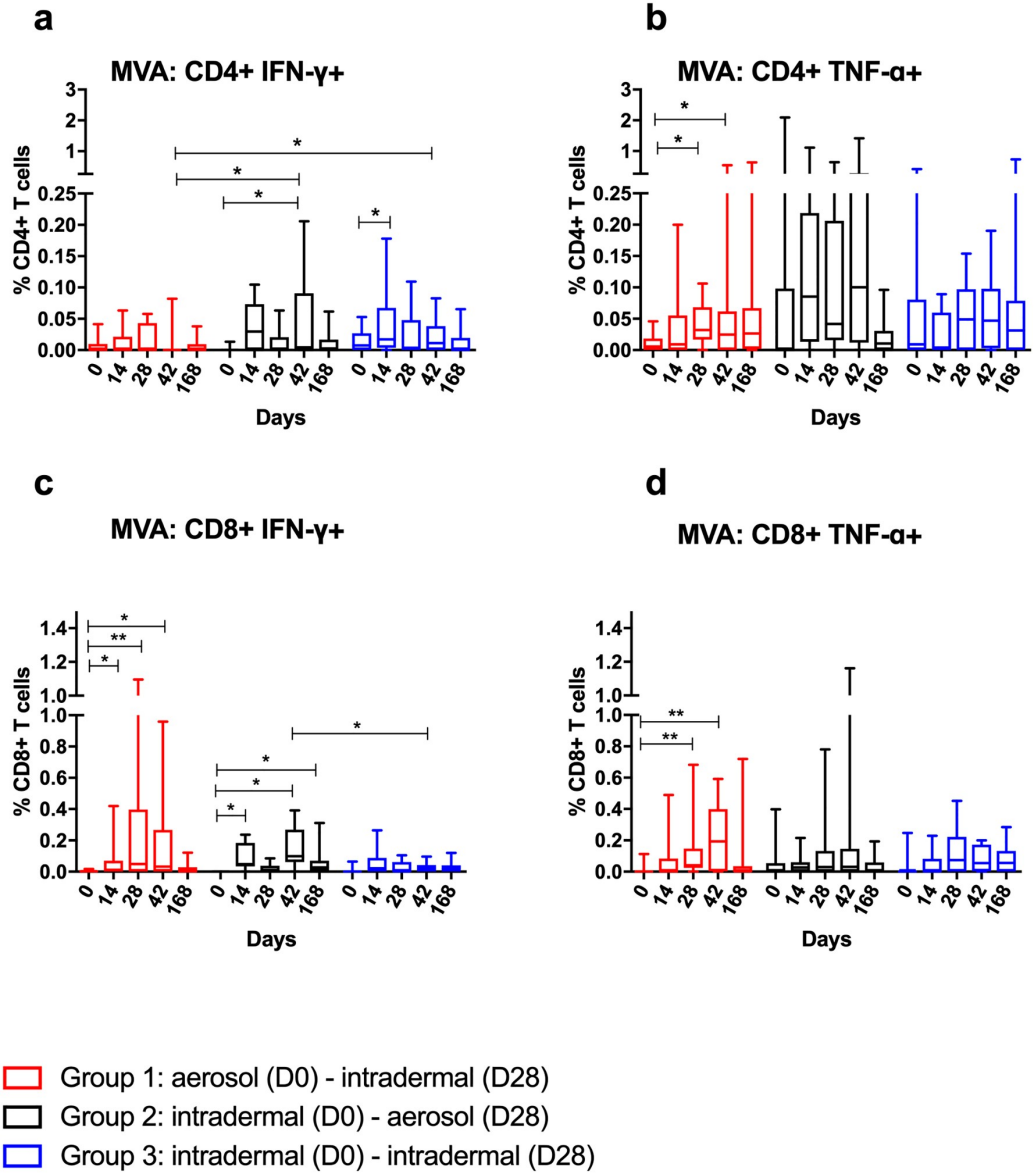
Whole blood ICS MVA-specific CD4+ and CD8+ T cells positive for IFNγ and TNFα. *X* axis = time points in days, *Y* axis = percent of CD4+ (a and b) or CD8+ T (c and d) cells positive for that cytokine. Statistically significant differences from baseline and between groups are shown. Non-significant differences (*p* > 0.05) are omitted for clarity. *p < 0.05, **p < 0.01. Note the different *Y* axis scales. Ag85A, antigen 85A; BAL, bronchoalveolar lavage; ICS, intracellular cytokine staining; MVA, modified vaccinia virus Ankara.

Median AUC analysis of MVA-specific CD4+ and CD8+ T cell IFNγ ELISpot responses demonstrated no significant differences between groups apart from MVA-specific CD8+ T cell IFNγ responses between Groups 2 and 3 (*p* = 0.012) (S8 and S10 Tables). CD4+ T cell responses were significantly higher than baseline on D42 in Group 2 (*p* = 0.031) and on D14 in Group 3 (*p* = 0.037) (Fig 5). MVA-specific TNFα+ CD4+ T cell responses were significantly higher than baseline in Group 1 only on D28 and D42 (*p* = 0.020 and *p* = 0.037, respectively). There was no boosting of MVA-specific IFNγ+ or TNFα+ CD4+ T cell responses following the D28 vaccination in any group (D28 versus D42: all *p* > 0.05).

MVA-specific IFNγ+ CD8+ T cell responses increased above baseline following the D0 vaccination in Groups 1 and 2 on D14 (D0 versus D14: Group 1, *p* = 0.020; Group 2, *p* = 0.016) (Fig 5). The response in Group 2 increased following the aerosol boost vaccination (D28 versus D42: *p* = 0.016). Group 2 was the only group to have detectable MVA-specific IFNγ+ CD8+ T cell responses above baseline at D168 (D0 versus D168: *p* = 0.016). MVA-specific TNFα+ CD8+ T cell responses increased at D28 and D42 in Group 1 (D28: *p* = 0.002; D42: *p* = 0.004).

MVA-specific IL2+ and IL17+ CD4+ and CD8+ T cell responses were not detectable.

Median AUC analysis of MVA-specific CD4+ and CD8+ T cell IFNγ ELISpot responses demonstrated no significant differences between groups apart from MVA-specific CD8+ T cell IFNγ responses between Groups 2 and 3 (*p* = 0.012) (S8 and S10 Tables).

### BAL intracellular cytokine responses

ICS was performed on freshly isolated BAL cells. The median of recovered BAL cells was 5.325 × 10^6^ (range = 1.8 × 10^6^ to 37 × 10^6^). The frequency of CD3+ T cells in the BAL fluid was higher after aerosol vaccination (Group 1 [D7] and Group 2 [D35]) than after intradermal vaccination (Groups 2 and 3 [D7] and Group 3 [D35]) (*p* = 0.016, 95% CI: 1.9% to 20.9%) (S3 Fig).

**Fig 6:**
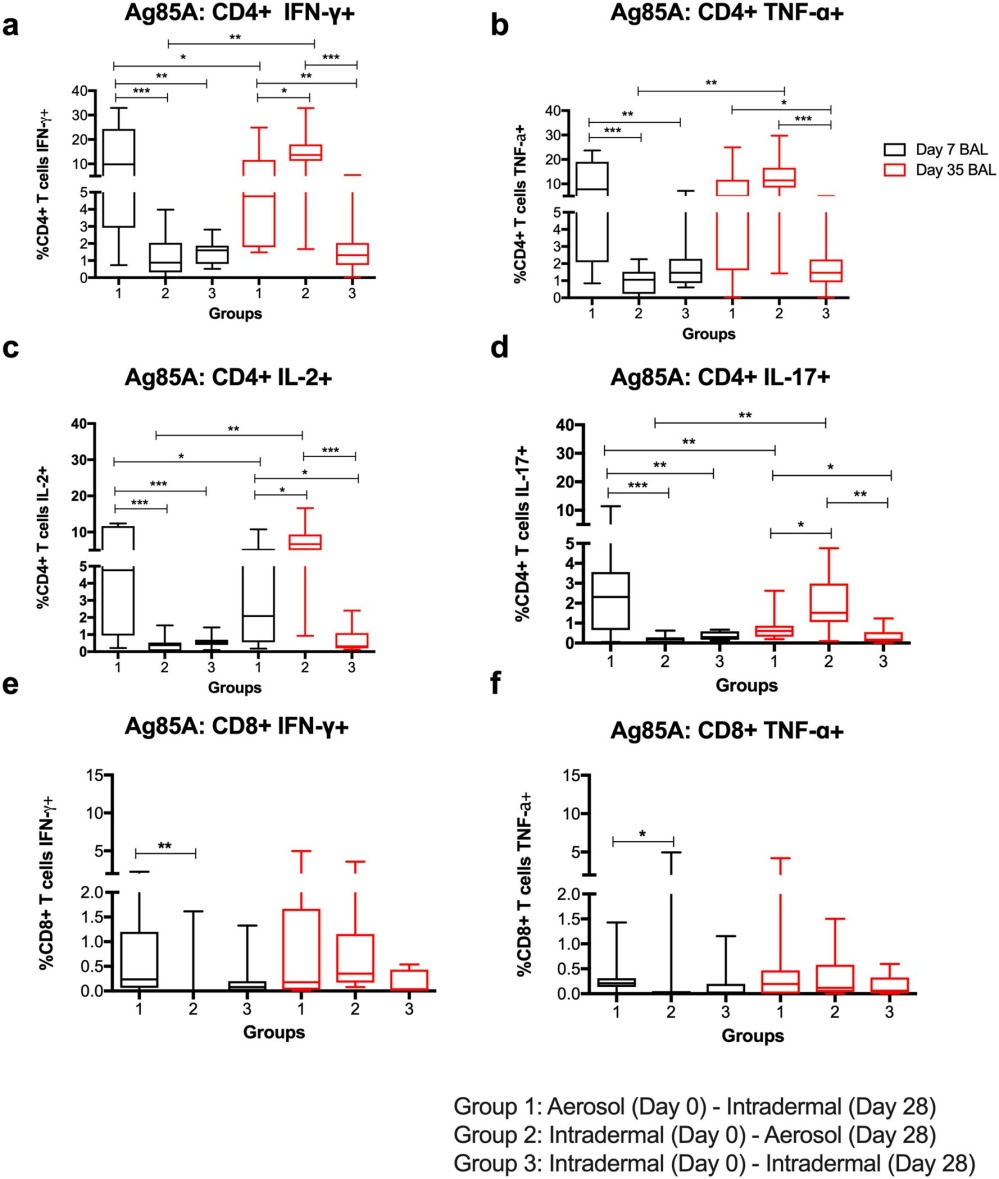
BAL Ag85A-specific CD4+ T cell responses. BAL Ag85A-specific CD4+ T cells positive for IFNγ (a), TNFα (b), IL2 (c), and IL17 (d) and Ag85A-specific CD8+ T cells positive for IFNγ (e) and TNFα (f). Non-significant differences (*p* > 0.05) are omitted for clarity. *p < 0.05, **p < 0.01, ***p < 0.001. Note the different *Y* axis scales. Ag85A, antigen 85A; BAL, bronchoalveolar lavage.

In the D7 BAL samples from Group 1 (aerosol prime), there was a significantly higher frequency of Ag85A-specific CD4+ T cells positive for all cytokines compared with Groups 2 and 3 (intradermal prime) (Fig 6; Table 1). Following the D28 boost vaccination, the frequency of cytokine positive Ag85A-specific CD4+ T cells was significantly higher in Group 2 (aerosol boost) (Fig 6; Table 1).

**Table 1:** Bronchoalveolar lavage Ag85A-specific CD4+ T cell responses (Mann–Whitney test).

Cytokine	Measure	Group 1 versus Group 2	Group 2 versus Group 3	Group 1 versus Group 3
**Day 7**				
IFNγ	*p*-Value	<0.001		0.001
	95% CI	2.013 to 21.390		1.350 to 20.930
TNFα	*p*-Value	0.001		0.008
	95% CI	1.090 to 16.310		0.513 to 15.480
IL-2	*p*-Value	<0.001		0.001
	95% CI	0.681 to 10.800		0.513 to 10.510
IL-17	*p*-Value	0.001		0.008
	95% CI	0.498 to 3.333		0.337 to 3.182
**Day 35**				
IFNγ	*p*-Value	0.041	<0.001	0.003
	95% CI	0.124 to 13.250	9.578 to 16.700	0.649 to 10.200
TNFα	*p*-Value	0.129	<0.001	0.037
	95% CI	−1.495 to 11.860	6.644 to 15.050	0.158 to 10.170
IL-2	*p*-Value	0.018	<0.001	0.011
	95% CI	0.742 to 7.354	4.387 to 8.085	0.166 to 3.617
IL-17	*p*-Value	0.028	0.003	0.025
	95% CI	0.230 to 2.134	0.701 to 2.528	0.045 to 0.724

Confidence intervals are percentage of cytokine+ CD4+ T cells.

Ag85A, antigen 85A.

D35 BAL Ag85A-specific CD4+ T cell responses in Group 2 were higher than those on D7 (D7 versus D35: *p* = 0.004 for all 4 cytokines). However, the D35 BAL Ag85A-specific CD4+ T cell responses in Group 2 were not significantly different from the D7 BAL responses in Group 1 for any cytokine.

Aerosol vaccination was more potent at inducing lung mucosal CD8+ T cell responses. Ag85A-specific IFNγ and TNFα CD8+ T cell responses in the D7 BAL were highest following aerosol prime vaccination (Group 1), but were only significantly different to Group 2 (IFNγ, *p* = 0.002; TNFα, *p* = 0.01; Fig 6). After the D28 boost vaccination, there was a tendency for higher responses in Groups 1 and 2 compared to Group 3, but this difference was not statistically significant.

**Fig 7:**
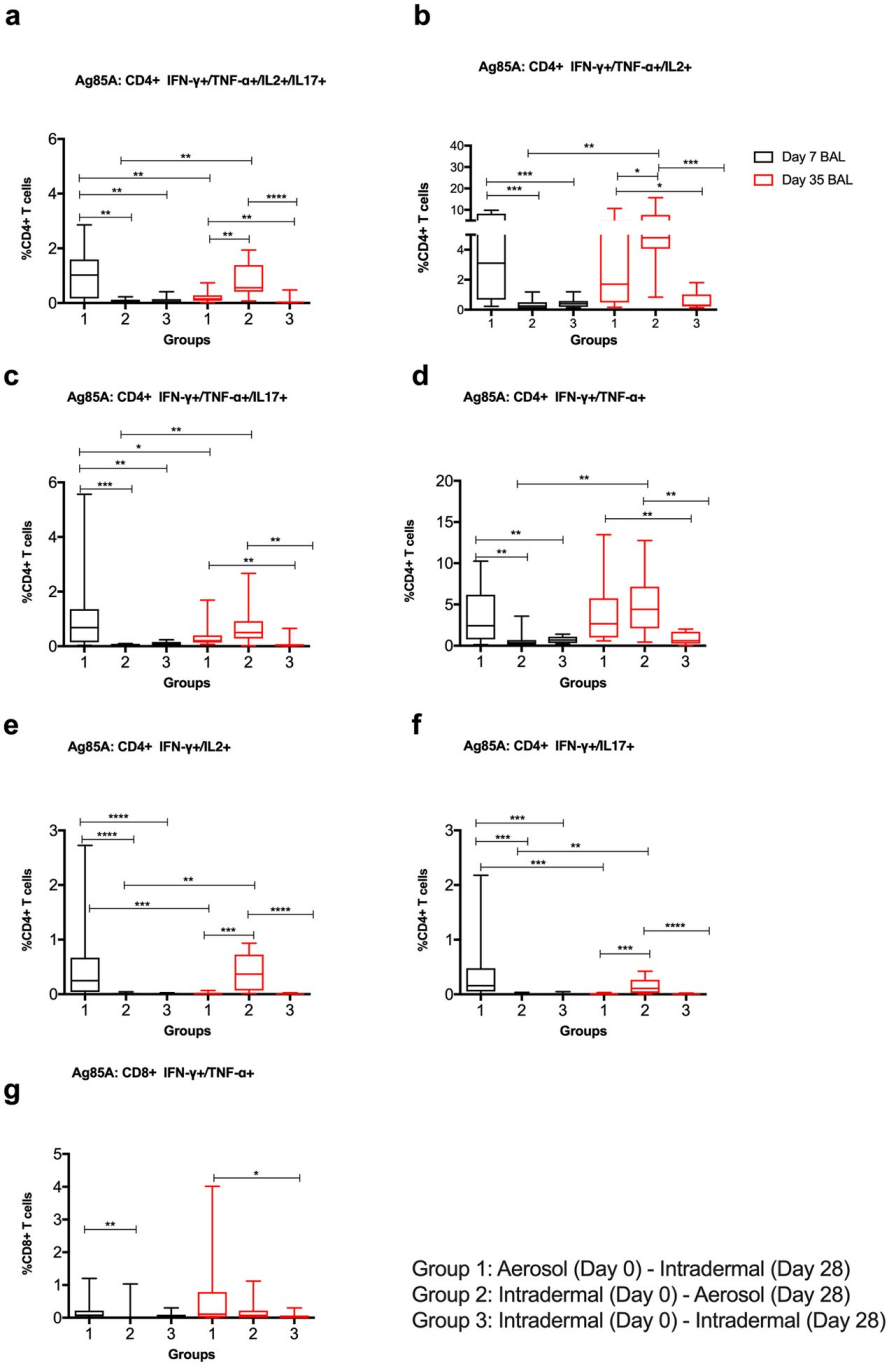
BAL Ag85A-specific polyfunctional CD4+ and CD8+ T cell responses. (a) CD4+ IFNγ+ TNFα+ IL2+ IL17+; (b) CD4+ IFNγ+ TNFα+ IL2+, (c) CD4+ IFNγ+ TNFα+ IL17+; (d) CD4+ IFNγ+ TNFα+; (e) CD4+ IFNγ+ IL2+, (f) CD4+ IFNγ+ IL17+; (g) CD8+ IFNγ+ TNFα+. Non-significant differences (*p* > 0.05) are omitted for clarity. *p < 0.05, **p < 0.01, ***p < 0.001. Note the different *Y* axis scales. Ag85A, antigen 85A; BAL, bronchoalveolar lavage.

Following the D0 vaccination, polyfunctional CD4+ T cells positive for multiple or dual cytokines were only detected in BAL cells of Group 1 volunteers at D7 (Fig 7). Following the D28 boost vaccination, responses were detectable in Groups 1 and 2 and were higher than in Group 3 for the detectable cytokine combinations (Fig 7). Responses in Group 2 were significantly higher than responses in Groups 1 and 3 for IFNγ+ TNFα+ IL2+ IL17+, IFNγ+ TNFα+ IL2+, IFNγ+ IL2+, and IFNγ+ IL17+ (Fig 7a, 7b, 7e, and 7f). Polyfunctional Ag85A-specific CD8+ T cells positive for IFNγ and TNFα had high responses following aerosol vaccination (Fig 7g).

**Fig 8:**
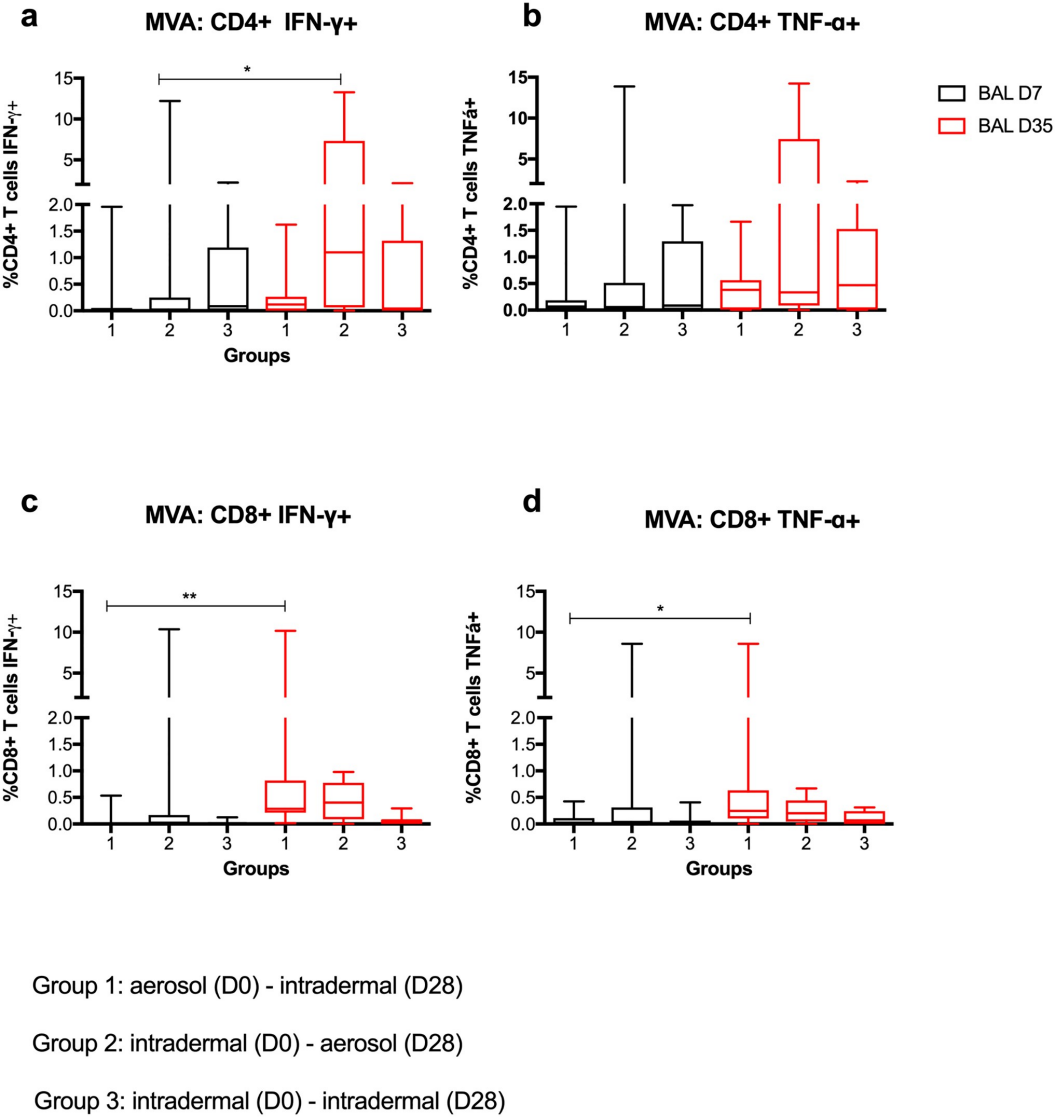
BAL MVA-specific CD4+ T cell responses. BAL MVA-specific CD4+ T cell responses (a and b) and CD8+ T cell responses (c and d) positive for cytokines IFNγ and TNFα. Non-significant differences (*p* > 0.05) are omitted for clarity. *p < 0.05, **p < 0.01. Ag85A, antigen 85A; BAL, bronchoalveolar lavage; MVA, modified vaccinia virus Ankara.

The magnitude of MVA-specific CD4+ T cell responses detected by BAL ICS was lower than the Ag85A-specific responses (Fig 8). MVA-specific IFNγ+ CD4+ T cell responses in Group 2 on D35 were significantly higher than on D7 (*p* = 0.016). The results followed a similar pattern for the other 3 cytokines, although did not reach statistical significance (Fig 8). MVA-specific IFNγ+ and TNFα+ CD8+ T cell responses in Group 1 were higher following intradermal boost on D35 than on D7 (*p* = 0.007 and *p* = 0.014 for IFNγ and TNFα, respectively). No Ag85A-specific and MVA-specific IL2+ or IL17+ CD8+ T cells were detected in the BAL samples. IFNγ was the most frequent cytokine detected in BAL cells. Mucosal Ag85A-specific IFNγ correlated positively with peripheral blood IFNγ response at both D7 (*p* = 0.001, *r* = 0.553) and D35 (*p* = 0.008, *r* = 0.474).

### ELISA

**Fig 9:**
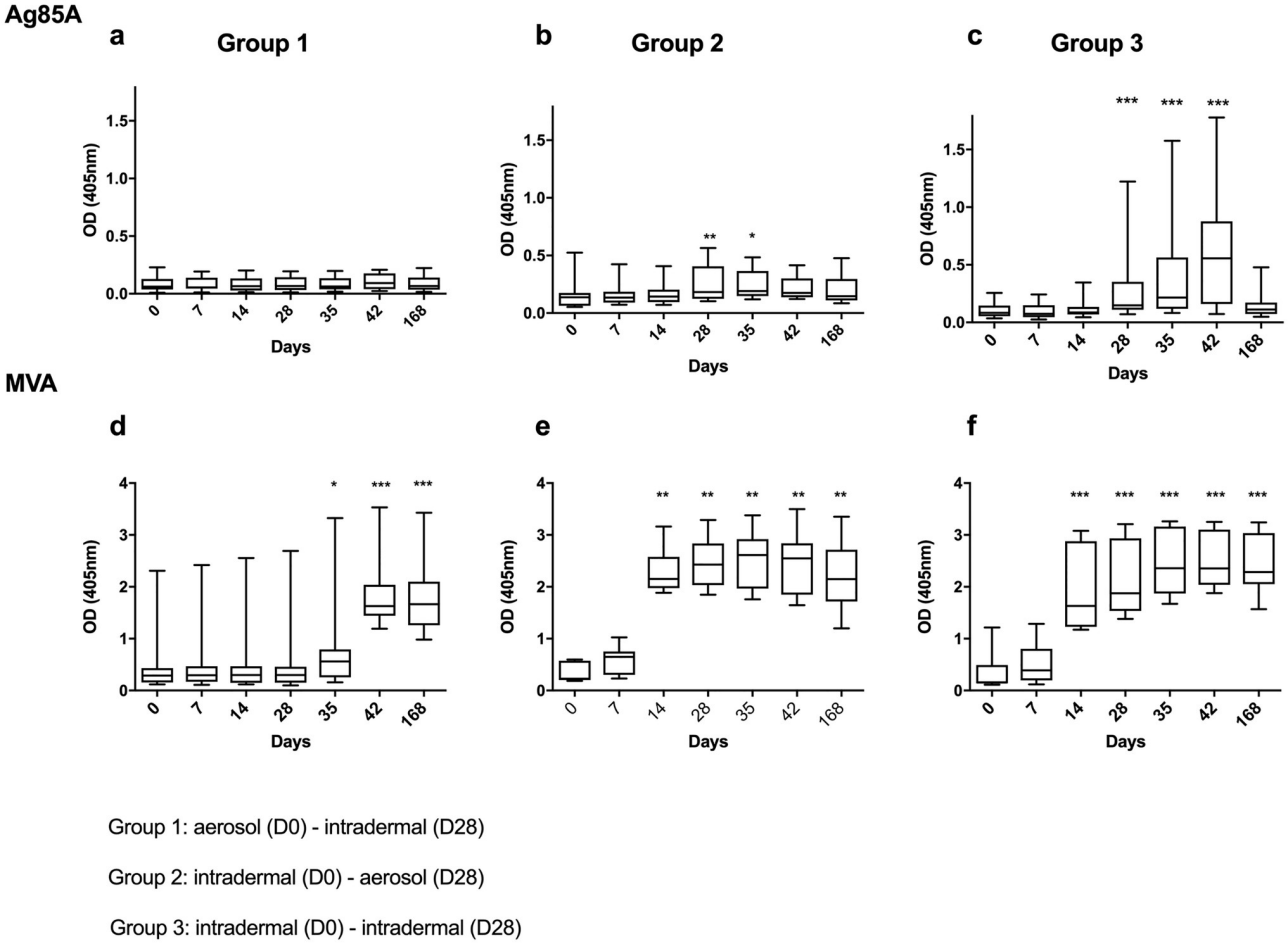
Serum IgG responses. Responses against Ag85A (a–c) and MVA (d–f) for Groups 1, 2, and 3 (from left to right). *X* axis = time point in days, *Y* axis = OD read at 405 nm. Non-significant differences (*p* > 0.05) are omitted for clarity. *p < 0.05, **p < 0.01, ***p < 0.001. *p*-Values denote significant differences from day 0. Note the different *Y* axis scales. Ag85A, antigen 85A; MVA, modified vaccinia virus Ankara; OD, optical density.

There were no significant differences between the 3 groups in baseline levels of anti-Ag85A or anti-MVA antibodies of the 2 isotypes IgG and IgA (all *p* > 0.05) (Fig 9; S11 Table).

Anti-Ag85A serum IgG responses peaked at D28 after D0 intradermal but not aerosol prime vaccination (Fig 9; D0 versus D28: Group 2, *p* = 0.004; Group 3, *p* = 0.001). Intradermal boosting in Group 3 resulted in a significant increase in Ag85A-specific IgG responses (Fig 9; D28 versus D42: *p* = 0.002). Median AUC analysis of Ag85A-specific IgG responses showed significant differences between Groups 1 and 2 (*p* = 0.004, 95% CI: 7.850 to 32.200 OD) and between Groups 1 and 3 (*p* = 0.003, 95% CI: 8.070 to 64.380 OD).

Anti-MVA serum IgG antibodies were induced by intradermal but not aerosol vaccination (Fig 9). Levels were detectable from D14 after the D0 intradermal prime vaccinations in Groups 2 and 3, and in Group 1 from D35 after the D28 intradermal boost vaccination, peaking on D42 (Fig 9; D0 versus D28: Group 2, *p* = 0.004; Group 3, *p* = 0.001; D0 versus D42: Group 1, *p* = 0.001). Median AUC analysis of MVA-specific IgG responses demonstrated significant differences between Groups 1 and 2 (*p* = 0.012, 95% CI: 38.100 to 206.700 OD) and between Groups 1 and 3 (*p* = 0.001, 95% CI: 69.400 to 229.800 OD).

The pattern of responses was similar for IgA (S11 Table). ELISAs were also performed on thawed BAL fluid, but responses were below the limit of detection. There were no significant correlations between vector and insert responses.

## Discussion

This clinical trial demonstrates that aerosol delivery of MVA85A as a priming vaccination has an acceptable AE profile, supporting the results of our first phase I study [19]. Further, we show that both the heterologous aerosol–intradermal vaccination regime and the homologous intradermal–intradermal Group 3 regime are well tolerated and feasible. Unfortunately, the profile of AEs induced by aerosolised MVA85A as a boost vaccination 1 month after an intradermal prime does not support the further development of this specific vaccination regimen.

For all groups, the most frequently occurring AEs were mild local reactions at the injection site after intradermal MVA85A vaccination. When comparing regimens, the only significant difference in any AEs was injection-site-related AEs in Group 3, the only group that received 2 MVA85A injections. However, when vaccinations were compared individually, the D28 boost vaccine in the intradermal–aerosol regimen (Group 2) induced transient but clinically significant respiratory AEs in 7 of 9 volunteers. With larger numbers, these AEs may have translated into significant differences across regimens. There were no SAEs in this study, and volunteers each underwent 2 bronchoscopies during the study period with no complications.

Delivering MVA85A by aerosol was a potent route to induce higher frequencies of mucosal Ag85A-specific T cell responses than intradermal immunisation, and induced comparable systemic Ag85A-specific T cell responses. However these responses did not seem to be attenuated by heterologous vaccination (either pre- or post-intradermal vaccination).

The aetiology of the unexpected respiratory AEs in Group 2 is unclear. Potential causes include a response to the MVA vector or hypersensitivity pneumonitis (HP) due to either the vector MVA or the insert Ag85A. The magnitude of cellular anti-vector responses observed in Group 2, in the lung and blood, would be in keeping with a heightened response on re-exposure. HP due to Ag85A is a potential concern in people with latent *M*.*tb* infection because of the potential for a Koch reaction or immunopathology at the site of infection. Preliminary results from a trial undertaken in our group evaluating the safety and immunogenicity of a single MVA85A immunisation delivered by aerosol in adults with latent *M*.*tb* infection do not demonstrate any safety concerns (ClinicalTrials.gov NCT02532036) [21]. Other possibilities include HP due to MVA, Ag85A, or any remnant chick embryonic fibroblast protein from the MVA manufacturing process. HP to avian protein has been previously described [22–25]. Adjusting the dose, adjusting the boosting interval, or using alternative viral vectors to prime and boost might mitigate the AE profile seen in this intradermal–aerosol regimen.

Induction of higher frequencies of mucosal Ag85A-specific T cell responses, and induction of comparable systemic Ag85A-specific T cell responses, following MVA85A aerosol delivery compared with intradermal immunisation supports the findings of our previous aerosol MVA85A study [19]. The mucosal immune response in Group 2 was higher in the D35 BAL than in the D7 BAL, suggesting that aerosol MVA85A, given as a boost to an intradermal priming vaccination, was the most potent way to induce high levels of Ag85A-specific T cell responses in the lung mucosa. However, the aerosol boost BAL responses in Group 2 were not significantly different from the aerosol prime BAL responses in Group 1 for any cytokine. Mucosal polyfunctional T cells were enhanced following MVA85A aerosol administration. The role of polyfunctional T cells in protection against TB is controversial, although these cells were associated with BCG-induced protection in a murine *M*.*tb* infection model [26]. In BCG-vaccinated South African infants, there was no correlation between polyfunctional BCG-specific peripheral blood CD4+ T cells and protection against TB [27]. The significance of the mucosal MVA85A-induced polyfunctional T cells demonstrated in this study remains to be investigated further.

In the blood, there was an increased Ag85A ELISpot response after the boost vaccination that was only detected in Group 2. However, there was no significant systemic boost on WB ICS detectable in Group 2, and the magnitude of the boost detected on ELISpot was modest. It is possible that the primary intradermal administration in Group 2 did not elicit an immune response in the lung, and that the second administration was “perceived” as a prime administration by the immune system. It would be interesting to evaluate a longer interval between prime and boost immunisation to determine whether that would result in a greater boosting effect.

Delivering an aerosol prime and intradermal boost immunisation did not result in a significant boosting effect. These findings are consistent with preclinical studies, where only systemic priming and local (mucosal) boosting resulted in significant boosting [28–31]. BCG-specific ELISpot responses have been demonstrated to associate with a reduced risk of TB disease in South African infants, and efforts to optimise systemic and mucosal T cell responses continue [32].

IFNγ provided the clearest and strongest signal among the studied cytokines in both blood and BAL. TNFα production correlated with IFNγ production, whereas the signal from IL2 and IL17 was weaker. However we continue to measure IL2, for the importance of central memory T cells, and IL17, for evidence of a protective effect in TB [33,34].

The significance of humoral immunity in TB is uncertain. A recent case–control analysis of BCG-vaccinated infants from the infant MVA85A efficacy trial [32] demonstrated that Ag85A-specific IgG antibodies were associated with a reduced risk of TB disease. In the trial reported here, serum anti-Ag85A antibodies were only induced after intradermal vaccination, and serum anti-Ag85A antibodies were only boosted by a second intradermal MVA85A vaccination. This is an important finding for any vaccination regimen aiming to induce high levels of humoral immunity.

MVA-specific IFNγ ELISpot responses were induced by both routes of vaccination. Induction of systemic cellular immune responses to the MVA vector was demonstrated in all regimens, but was highest in participants who received an intradermal prime vaccination. Antibodies to the vector MVA were also only demonstrated after intradermal MVA85A vaccination, and only boosted in the intradermal–intradermal regimen. This is of relevance for any vaccination regimen aiming to circumvent local anti-vector immunity.

### Strengths and limitations

With this blinded randomised controlled clinical trial we have demonstrated the feasibility of performing aerosol-based vaccine experimental medicine trials that interrogate different routes of immunisation in healthy volunteers. There were no SAEs in this study, and volunteers each underwent 2 bronchoscopies during the study period with no complications. By performing 2 bronchoscopies in each volunteer, we were able to directly compare changes in mucosal response following each vaccination. Importantly, this study demonstrates that changing the route of vaccination in prime-boost regimens substantially impacts the AE profile, as was seen with the intradermal–aerosol (Group 2) versus aerosol–intradermal regimens (Group 1). Further, by comparing responses following heterologous delivery regimes, we were able to demonstrate that antibodies to the vector MVA were only detectable after intradermal MVA85A vaccination, and only boosted in the intradermal–intradermal regimen. This could have important implications in future vaccine design, where development of anti-vector immunity limits delivery of boost vaccinations.

The main limitations of this study are the inherent restrictions imposed by performing such clinical trials in healthy human volunteers. Additional mucosal immunology data prior to vaccination and at various time points following vaccination other than 7 days after each vaccination could have been informative, especially given that many systemic responses were no longer detectable at 6 months. The bronchoscopy was performed 1 week following vaccination as this time point was thought most likely to capture peak mucosal responses based on preclinical findings [11]. However peak mucosal responses may have been earlier or later, particularly given that systemic immune responses did not consistently peak at 7 days after vaccination. Only 9 of the planned 12 Group 2 volunteers received the intended regimen, and this smaller sample size may have impacted on our ability to reach statistical significance for differences in some of the outcomes. In light of the potent immune response induced by this regime but also the unforeseen respiratory symptoms reported, if capacity had allowed, a comparator aerosol prime–aerosol boost regimen may have been very informative. Further, the transient symptoms experienced by volunteers may have been ameliorated by altering the prime–boost interval.

In this phase I trial we describe, to our knowledge, the first sequential administration of aerosol and intradermal vaccinations using a candidate TB vaccine, and we demonstrate, for a second time, the safety and potent immunogenicity of the aerosol route as a priming immunisation in humans. The findings add to our understanding of cellular and humoral immune responses to vector and insert induced by aerosolised MVA85A as a prime or boost in a heterologous route regimen. Further studies are needed to characterise in more detail the mucosal and systemic immune response to aerosolised candidate TB vaccines, and to optimise the tolerability and immunogenicity of vaccination regimes. These findings are important for the development of mucosal vaccines against TB and other respiratory pathogens.

## Supporting information

S1 CONSORT Checklist (DOC)

S1 Fig ICS gating strategy. Lymphocytes were gated on a forward scatter area (FSC-A) versus side scatter (SSC) (a). Next, duplets were excluded on a forward scatter height (FSC-H) versus FSC-A (b). CD14+ and CD19+ cells were excluded by gating on CD3+ Dump− (CD14 and CD19) (c). For BAL samples, dead cells were also excluded. Within CD3+ lymphocytes, CD4+ and CD8+ subsets were determined (d), and this was followed by gating on cytokine+ CD4+ T cells (e–g) and cytokine+ CD8+ T cells (h–j).
(TIF)

S2 Fig Median percent change of FEV_1_ and FVC from baseline at each time point in the 7 days following vaccination by group. Baseline spirometry taken from the volunteer’s D0 pre-vaccination reading in all panels. Median with interquartile range. FEV_1_, forced expiratory volume in 1 second; FVC, forced vital capacity.
(TIF)

S3 Fig Total CD3+ T lymphocytes detected in D7 and D35 BAL by group. (TIF)

S1 Protocol Clinical trial protocol. A phase I trial evaluating mucosal administration of a candidate TB vaccine, MVA85A, as a way to induce potent local cellular immune responses and avoid anti-vector immunity.
(PDF)

S1 Table Baseline characteristics. (PDF)

S2 Table The numbers of participants within each group reporting each related AE. (PDF)

S3 Table Systemic AEs by group, by vaccination, and by severity. (PDF)

S4 Table Respiratory AEs by group, by vaccination, and by severity. (PDF)

S5 Table Solicited AEs by volunteer, after each vaccination. (PDF)

S6 Table Solicited AEs by volunteer, by group. (PDF)

S7 Table ELISpot responses statistical analysis: AUC. (PDF)

S8 Table WB intracellular cytokines statistical analysis: AUC. One-way ANOVA (Kruskal–Wallis) adjusted for multiple comparisons.
(PDF)

S9 Table WB intracellular cytokines statistical analysis: AUC—85A. Mann–Whitney test (AUC medians).
(PDF)

S10 Table WB intracellular cytokines statistical analysis: AUC—MVA. Mann–Whitney test (AUC medians).
(PDF)

S11 Table Ag85A- and MVA-specific serum IgA response for Groups 1, 2, and 3. (PDF)

## Abbreviations

AE: adverse event
Ag85A: antigen 85A
AUC: area under the curve
BAL: bronchoalveolar lavage
BCG: bacille Calmette-Guérin
CFP-10: culture filtrate protein–10
D[number]: day [number]
ELISA: enzyme-linked immunosorbent assay
ELISpot: enzyme-linked immunospot
ESAT-6: early secreted antigenic target–6
FEV_1_: forced expiratory volume in 1 second
FVC: forced vital capacity
HP: hypersensitivity pneumonitis
ICS: intracellular cytokine staining
*M.tb*: *Mycobacterium tuberculosis*
MVA: modified vaccinia virus Ankara
OD: optical density
PBMC: peripheral blood mononuclear cell
SAE: serious adverse event
SEB: staphylococcal enterotoxin B
SPC: spot forming cells
TB: tuberculosis
WB: whole blood
