# Supplementary material for: Alternate aerosol and systemic immunisation with a recombinant viral vector for tuberculosis, MVA85A: A phase I randomised controlled trial
Source: PLoS Med. 2019 Apr 30;16(4):e1002790. doi: 10.1371/journal.pmed.1002790 (PMC6490884; doi:10.1371/journal.pmed.1002790)
Supplement: S1 CONSORT Checklist — (DOC) [file pmed.1002790.s001.doc]

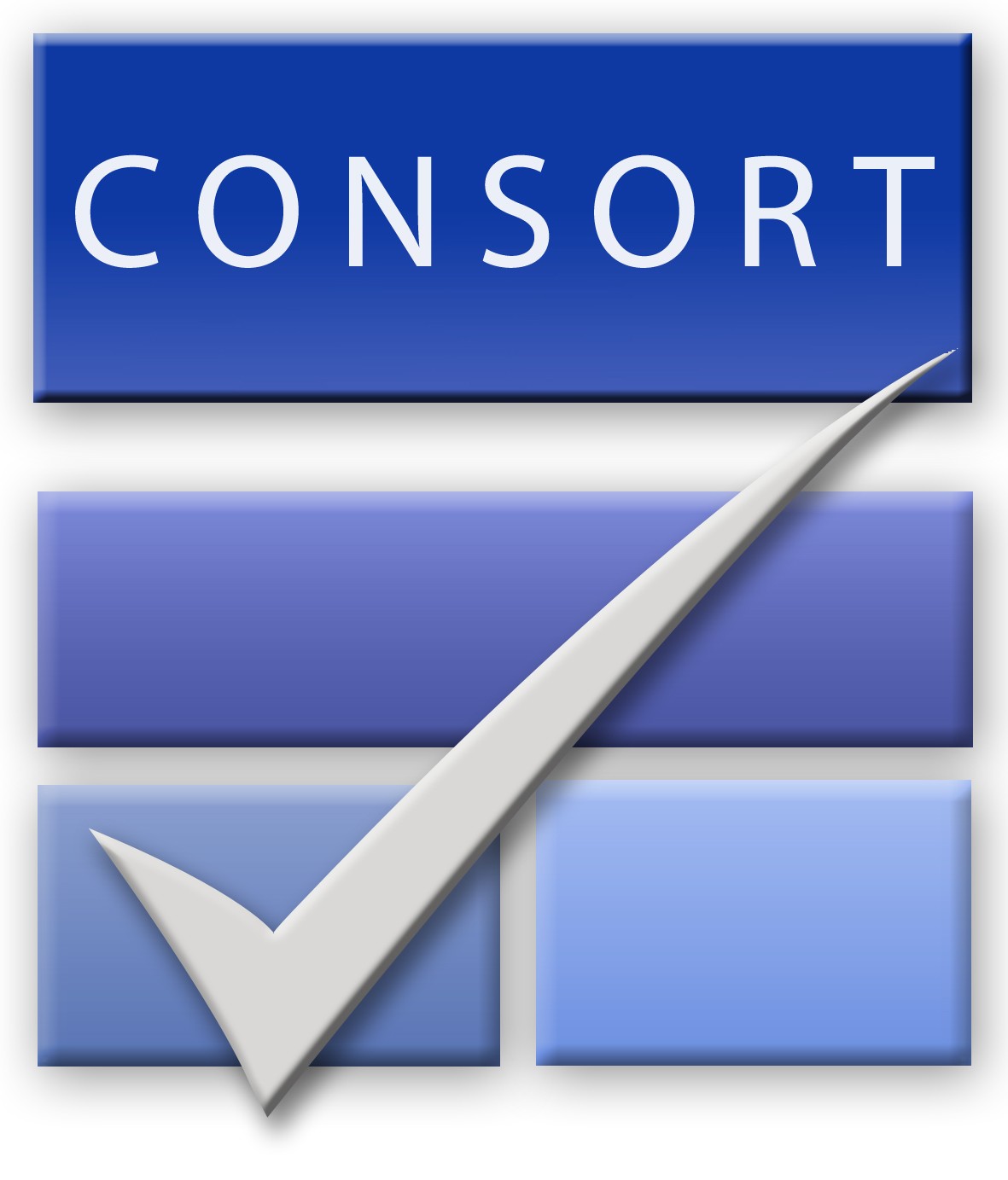
CONSORT 2010 checklist of information to include when reporting a randomised trial*

| Section/Topic | Item No | Checklist item | Reported on page No |
| --- | --- | --- | --- |
| Title and abstract | | | |
|  | 1a | Identification as a randomised trial in the title | Title |
|  | 1b | Structured summary of trial design, methods, results, and conclusions (for specific guidance see CONSORT for abstracts) | Abstract methods, findings and conclusion paragraphs |
| Introduction | | | |
| Background and objectives | 2a | Scientific background and explanation of rationale | Introduction paragraph 1, 2, 3 and 4 |
|  | 2b | Specific objectives or hypotheses | Introduction paragraph 5 |
| Methods | | | |
| Trial design | 3a | Description of trial design (such as parallel, factorial) including allocation ratio | Methods paragraph 1 and Randomisation and blinding paragraph |
|  | 3b | Important changes to methods after trial commencement (such as eligibility criteria), with reasons | Methods paragraph 3&4, Randomisation and blinding paragraph, Vaccination paragraph 2 |
| Participants | 4a | Eligibility criteria for participants | Methods paragraph 3 |
|  | 4b | Settings and locations where the data were collected | Methods paragraph 1 |
| Interventions | 5 | The interventions for each group with sufficient details to allow replication, including how and when they were actually administered | Methods paragraphs, Vaccination paragraphs, Bronchoscopy and BAL paragraphs, Clinical adverse event monitoring paragraph |
| Outcomes | 6a | Completely defined pre-specified primary and secondary outcome measures, including how and when they were assessed | Sample size and study endpoints paragraph, Clinical adverse event monitoring paragraph, Bronchoscopy and BAL paragraphs, Ex-vivo IFNy ELISpot paragraph, Statistical analysis paragraph |
|  | 6b | Any changes to trial outcomes after the trial commenced, with reasons | Methods paragraph 5, Randomisation and blinding paragraph, Vaccination paragraph 2 |
| Sample size | 7a | How sample size was determined | Sample size and study endpoints paragraph 1 and 2 |
|  | 7b | When applicable, explanation of any interim analyses and stopping guidelines | Methods paragraph 2, Randomisation and blinding paragraph |
| Randomisation: |  |  |  |
| Sequence generation | 8a | Method used to generate the random allocation sequence | Randomisation and blinding paragraph |
|  | 8b | Type of randomisation; details of any restriction (such as blocking and block size) | Randomisation and blinding paragraph |
| Allocation concealment mechanism | 9 | Mechanism used to implement the random allocation sequence (such as sequentially numbered containers), describing any steps taken to conceal the sequence until interventions were assigned | Randomisation and blinding paragraph |
| Implementation | 10 | Who generated the random allocation sequence, who enrolled participants, and who assigned participants to interventions | Randomisation and blinding paragraph |
| Blinding | 11a | If done, who was blinded after assignment to interventions (for example, participants, care providers, those assessing outcomes) and how | Randomisation and blinding paragraph, |
|  | 11b | If relevant, description of the similarity of interventions | Randomisation and blinding paragraph |
| Statistical methods | 12a | Statistical methods used to compare groups for primary and secondary outcomes | Vaccination paragraph |
|  | 12b | Methods for additional analyses, such as subgroup analyses and adjusted analyses | Statistical analysis paragraph 1 and 2 |
| Results | | | |
| Participant flow (a diagram is strongly recommended) | 13a | For each group, the numbers of participants who were randomly assigned, received intended treatment, and were analysed for the primary outcome | Fig 1 Consort diagram |
|  | 13b | For each group, losses and exclusions after randomisation, together with reasons | Fig 1 Consort diagram, Results participants paragraph |
| Recruitment | 14a | Dates defining the periods of recruitment and follow-up | Participants paragraph |
|  | 14b | Why the trial ended or was stopped | Participants paragraph |
| Baseline data | 15 | A table showing baseline demographic and clinical characteristics for each group | S1 table |
| Numbers analysed | 16 | For each group, number of participants (denominator) included in each analysis and whether the analysis was by original assigned groups | Statistical analysis paragraph 2, Fig 2, S2 Table, S3 Table, S4 Table |
| Outcomes and estimation | 17a | For each primary and secondary outcome, results for each group, and the estimated effect size and its precision (such as 95% confidence interval) | Fig 2, Clinical adverse events paragraphs, Ex-vivo Interferon-γ Enzyme-Linked ImmunoSpot paragraph and Fig 3 |
|  | 17b | For binary outcomes, presentation of both absolute and relative effect sizes is recommended | - |
| Ancillary analyses | 18 | Results of any other analyses performed, including subgroup analyses and adjusted analyses, distinguishing pre-specified from exploratory | Results paragraphs not specified above |
| Harms | 19 | All important harms or unintended effects in each group (for specific guidance see CONSORT for harms) | Clinical adverse events paragraphs |
| Discussion | | | |
| Limitations | 20 | Trial limitations, addressing sources of potential bias, imprecision, and, if relevant, multiplicity of analyses | Discussion Limitations paragraph |
| Generalisability | 21 | Generalisability (external validity, applicability) of the trial findings | Discussion strengths paragraph and concluding paragraph |
| Interpretation | 22 | Interpretation consistent with results, balancing benefits and harms, and considering other relevant evidence | Discussion paragraph 1-8 |
| Other information | | |  |
| Registration | 23 | Registration number and name of trial registry | Methods paragraph 1 |
| Protocol | 24 | Where the full trial protocol can be accessed, if available | Supporting information |
| Funding | 25 | Sources of funding and other support (such as supply of drugs), role of funders | Financial disclosure statement |

*We strongly recommend reading this statement in conjunction with the CONSORT 2010 Explanation and Elaboration for important clarifications on all the items. If relevant, we also recommend reading CONSORT extensions for cluster randomised trials, non-inferiority and equivalence trials, non-pharmacological treatments, herbal interventions, and pragmatic trials. Additional extensions are forthcoming: for those and for up to date references relevant to this checklist, see [www.consort-statement.org](http://www.consort-statement.org/).
