# Supplementary figures and images for: Alternate aerosol and systemic immunisation with a recombinant viral vector for tuberculosis, MVA85A: A phase I randomised controlled trial
Source: PLoS Med. 2019 Apr 30;16(4):e1002790. doi: 10.1371/journal.pmed.1002790 (PMC6490884; doi:10.1371/journal.pmed.1002790)

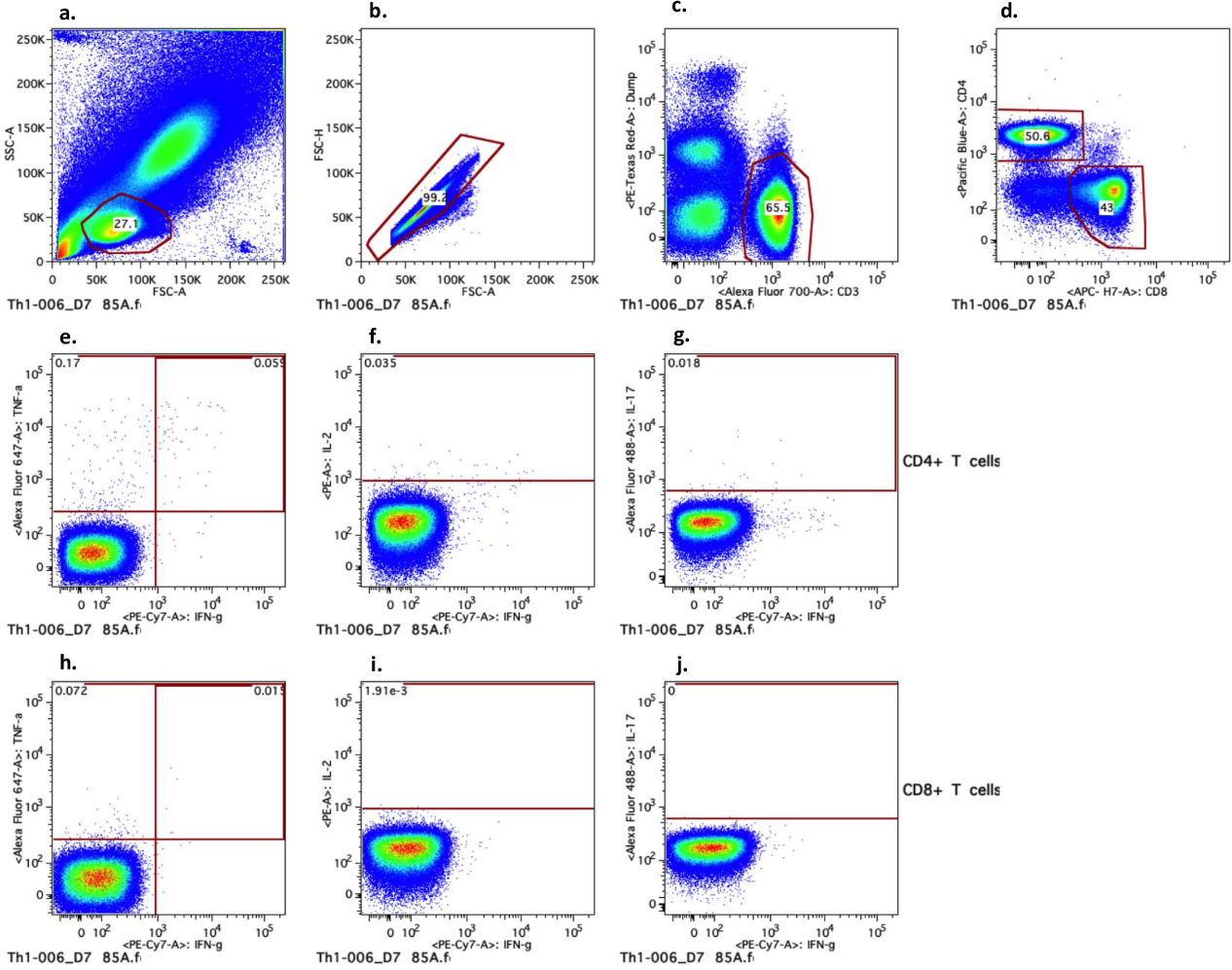

Supplement: S1 Fig — Lymphocytes were gated on a forward scatter area (FSC-A) versus side scatter (SSC) (a). Next, duplets were excluded on a forward scatter height (FSC-H) versus FSC-A (b). CD14+ and CD19+ cells were excluded by gating on CD3+ Dump− (CD14 and CD19) (c). For BAL samples, dead cells were also excluded. Within CD3+ lymphocytes, CD4+ and CD8+ subsets were determined (d), and this was followed by gating on cytokine+ CD4+ T cells (e–g) and cytokine+ CD8+ T cells (h–j). (TIF) [file pmed.1002790.s002.tif]

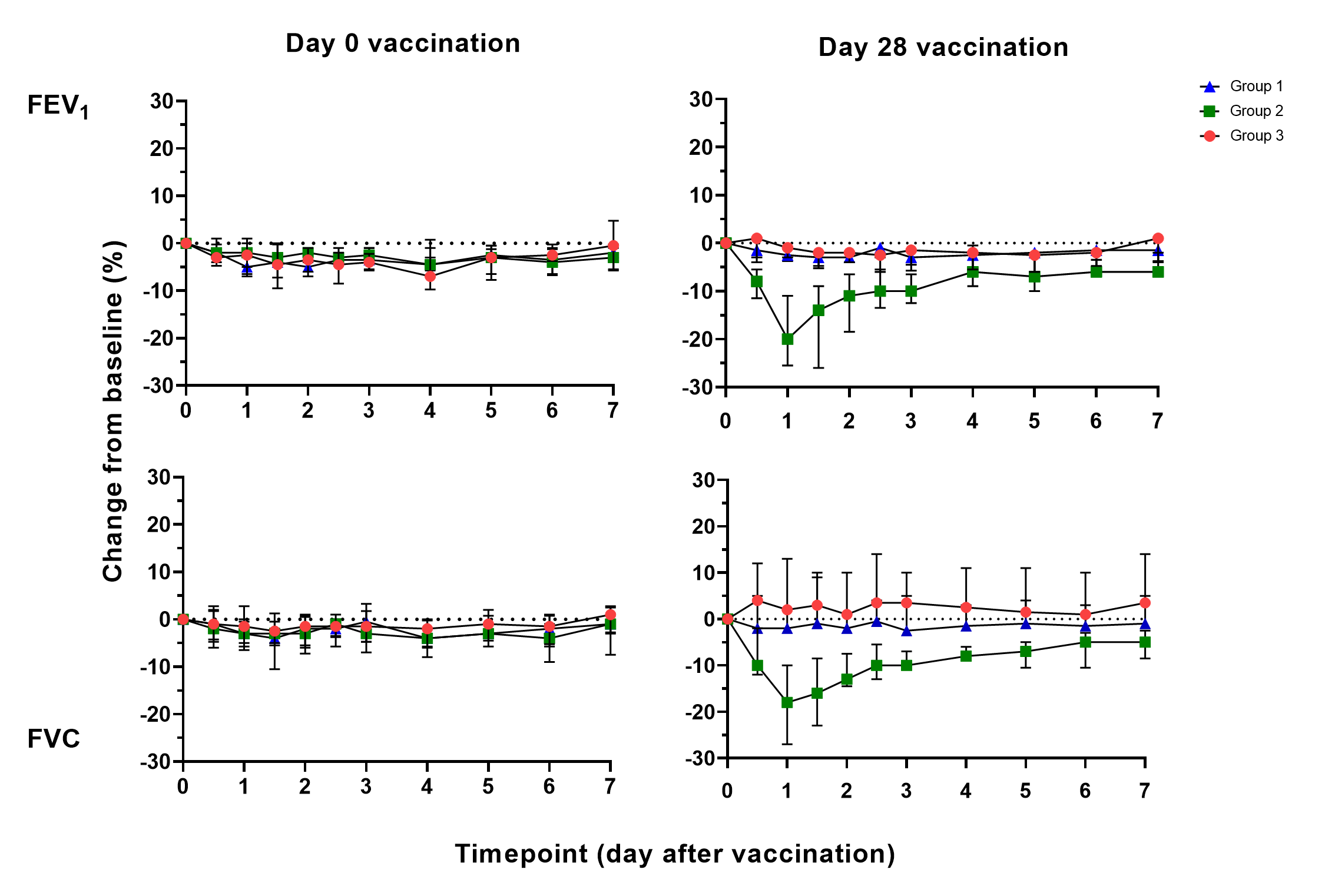

Supplement: S2 Fig — Baseline spirometry taken from the volunteer’s D0 pre-vaccination reading in all panels. Median with interquartile range. FEV1, forced expiratory volume in 1 second; FVC, forced vital capacity. (TIF) [file pmed.1002790.s003.tif]

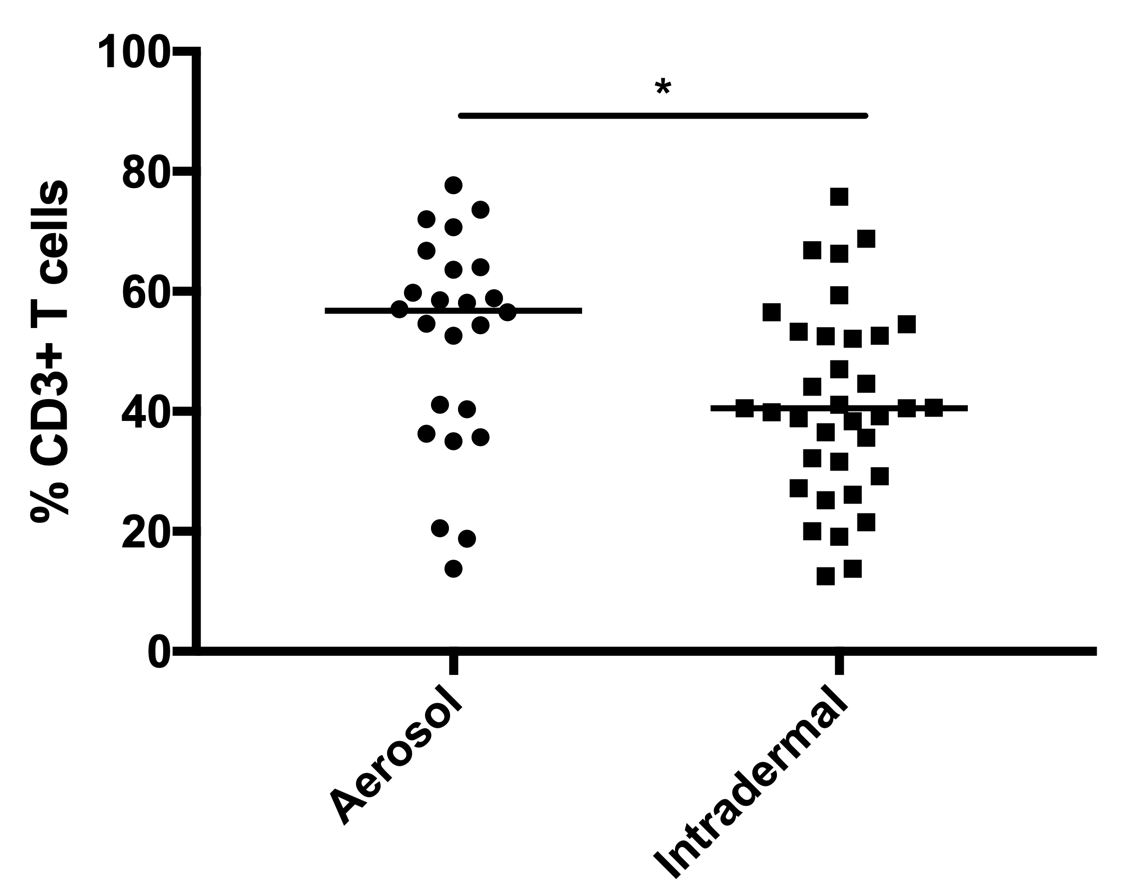

Supplement: S3 Fig — (TIF) [file pmed.1002790.s004.tif]
