# Supplementary material for: Alternate aerosol and systemic immunisation with a recombinant viral vector for tuberculosis, MVA85A: A phase I randomised controlled trial
Source: PLoS Med. 2019 Apr 30;16(4):e1002790. doi: 10.1371/journal.pmed.1002790 (PMC6490884; doi:10.1371/journal.pmed.1002790)
Supplement: S1 Protocol — A phase I trial evaluating mucosal administration of a candidate TB vaccine, MVA85A, as a way to induce potent local cellular immune responses and avoid anti-vector immunity. (PDF) [file pmed.1002790.s005.pdf]

CLINICAL TRIAL PROTOCOL

Trial reference: TB035

Title:
A Phase I trial evaluating mucosal administration of a candidate TB vaccine, MVA85A, as a way to induce potent local cellular immune responses and avoid anti-vector immunity

Trial Reference:	TB035
Protocol Version Number:	4.1
Date:	13th November 2015
EudraCT number:	2013-002020-16
REC Reference:	13/SC/0329
Sponsor:	University of Oxford
Chief Investigator:	Prof Helen McShane
Local Safety Committee Chair:	Dr Brian Angus
Funding body:	Wellcome Trust, NIHR BRC
Author:	Dr Zita-Rose Manjaly Thomas

STATEMENT OF COMPLIANCE

Investigator Agreement

"I have read this protocol and agree to abide by all provisions set forth therein. I agree to comply with the International Conference on Harmonisation Tripartite Guideline on Good Clinical Practice."

Prof Helen McShane	[signature]	14.11.15
Chief Investigator	Investigator Signature	Date

Confidentiality Statement

This document contains confidential information that must not be disclosed to anyone other than the Sponsor, the Investigator Team, and members of the Independent Ethics Committee. This information cannot be used for any purpose other than the evaluation or conduct of the clinical investigation without the prior written consent of Prof Helen McShane.

Table of Contents

Statement of Compliance **2**

Contents **3**

Key Roles and General Information **5**

List of abbreviations **7**

List of protocol amendments **8**

1. Trial Summary **10**

2. Background and Rationale **13**

2.1 Introduction 13
2.2 Hypothesis 20

3. Objectives and outcome measures/ Endpoints **20**

4. Trial design **21**

4.1 Trial Overview 21
Trial groups 21
4.2 Randomisation 22
4.3 Vaccine dosage 22
4.4 Risks and Benefits 23
4.5 Data Collection 25
4.6 Duration of Study 25
4.7 Definition of start and end of the trial 25

5 Selection and Withdrawal of Subjects **25**

5.1 Inclusion Criteria 25
5.2 Exclusion Criteria 26
5.3 Discontinuation/Withdrawal of Participants from Trial Treatment 27
5.4 Vaccination postponement Criteria 27
5.5 Bronchoscopy postponement criteria 28

6 Trial Vaccine, Medicines & Devices **28**

6.1 Candidate TB vaccine MVA85A 28
6.2 Saline 29
6.3 MicroAIR NE-U22 30
6.4 Sedative & anaesthetic agents for bronchoscopy 30

7 Trial Schedule **30**

7.1 Recruitment 30
7.2 Screening 31
7.3 Enrolment 32
7.4 Safety Review 32
7.5 Follow-up 32

8. Safety Reporting **32**

8.1 Definitions 32
8.2 Causality Assessment 33
8.3 Clinical Assessment 34
8.4 Severity Assessment 35
8.5 Laboratory Assessments 35

8.6 Reporting Procedure for Adverse Events ... 36
8. 7 Reporting of Serious Adverse Events ... 36
8.8 Reporting Procedures for SUSARs ... 37
8.9 Local Safety Committee ... 37
8.10 Development Safety Update report ... 37
8.11 Pregnancy ... 37
9. Statistics ... 37
9.1 Statistics ... 37
10. Data Management ... 38
10.1 Access to Data ... 38
10.2 Data Recording and Record Keeping ... 38
10.3 Source documents ... 39
10.4 Data Protection ... 39
11. Quality Assurance and Quality control ... 39
11.1. Quality Assurance ... 39
11.2. Monitoring ... 39
12. Ethical and Regulatory Considerations ... 40
12.1. Good Clinical Practice ... 40
12.2. Ethical Review ... 40
12.3. Informed Consent ... 40
12.4. Serious Breaches ... 40
13. Finance/Compensation/Insurance ... 40
13.1. Finance ... 40
13.2. Compensation ... 41
13.3. Insurance ... 41
14. Publication Policy ... 41
15. References ... 42

Key Roles and General Information

**Trial Centres:**	Centre for Clinical Vaccinology and Tropical Medicine (CCVTM)<br>& Oxford Centre for Respiratory Medicine<br>Churchill Hospital, Old Road, Headington<br>Oxford, OX3 7LE
**Chief Investigator:**	Prof Helen McShane<br>CCVTM
**Lead Investigator:**	Dr Zita-Rose Manjaly Thomas<br>CCVTM
**Other investigators:**	Dr Alice Minhinnick<br>TB vaccine trial physician<br>CCVTM<br><br>Dr Morven Wilkie<br>TB vaccine trial physician<br>CCVTM<br><br>Ian Poulton<br>Senior research nurse<br>CCVTM
**Collaborator(s):**	Dr Henry Bettinson<br>Consultant Respiratory and Intensive Care Physician<br>Oxford Centre for Respiratory Medicine,<br>Oxford University Hospitals NHS Trust<br>Oxford
**Project Managers:**	Dr Alison Lawrie & Mrs Samantha Vermaak<br>CCVTM
**Local Safety Monitor:**	Dr Brian Angus<br>Nuffield Department of Medicine<br>University of Oxford<br>Old Road,<br>Oxford, OX3 7BN
**Senior Immunologist:**	Dr Iman Satti<br>The Jenner Institute<br>ORCRB, University of Oxford<br>Roosevelt Drive<br>Oxford, OX3 7DQ
**Sponsor:**	Clinical Trials and Research Governance<br>Joint Research Office, University of Oxford<br>Block 60, Churchill Hospital<br>Old Road, Headington<br>Oxford OX3 7LE
**Monitor:**	Clinical Trials & Research Governance Office<br>Joint Research Office<br>University of Oxford<br>Block 60, Churchill Hospital

Old Road, Headington
Oxford OX3 7LE

List of abbreviations

**AE**	Adverse Event
**ALT**	Alanine Aminotransferase
**BAL**	Bronchoalveolar Lavage
**BCG**	Bacille Calmette-Guérin
**CCVTM**	Centre for Clinical Vaccinology and Tropical Medicine
**CFP-10**	Culture filtrate protein 10kDa
**CFU**	Colony-forming unit
**CRF**	Case Report Form
**CXR**	Chest X Ray
**DNA**	Deoxyribonucleic Acid
**EC**	Ethics Committee
**ELISPOT**	Enzyme-linked Immunospot
**ESAT-6**	Early-secreted antigenic target 6kDa protein
**FBC**	Full Blood Count
**GCP**	Good Clinical Practice
**GMO**	Genetically Modified Organism
**GLP**	Good Laboratory Practice
**GMP**	Good Manufacturing Practice
**GMSC**	Genetic Modification Safety Committee
**GP**	General Practitioner (Family Doctor)
**HBsAg**	Hepatitis B Surface Antigen
**HBV**	Hepatitis B Virus
**HCG**	Human Chorionic Gonadotrophin
**HCV**	Hepatitis C Virus
**HIV**	Human Immunodeficiency Virus
**HLA**	Human Leukocyte Antigen
**ICH**	International Committee on Harmonisation
**ID**	Intradermal
**IFN-γ**	Interferon Gamma
**IMP**	Investigational Medicinal Product
**INH**	Inhaled
**LFT**	Liver Function Test
**LSC**	Local Safety Committee
**LSM**	Local Safety Monitor
M. tb	*Mycobacterium tuberculosis*
**MVA**	Modified vaccinia Virus Ankara
**NHS**	National Health Service
**PBMC**	Peripheral Blood Mononuclear Cells
**PFT**	Pulmonary Function Tests
**QP**	Qualified person
**SAE**	Serious Adverse Event
**SFC**	Spot-forming cell
**SOP**	Standard Operating Procedure
**SUSAR**	Suspected Unexpected Serious Adverse Event
**TB**	Tuberculosis

Protocol v4.1 Non-Substantial Amendment

Section	From	Changed To
10	Data will be stored in either paper CRFs held in a key-locked cabinet at the CCVTM, or electronic CRFs on the OpenClinica™ database (which is currently being piloted at the CCVTM and which is stored electronically on a secure encrypted University of Oxford server), or both	Data will be stored in either paper CRFs held in a key-locked cabinet at the CCVTM, or electronic CRFs on the OpenClinica™ database (which is stored electronically on secure servers outsourced by OpenClinica™), or both

Protocol v 4.0 Substantial Amendment

Section	From	Changed To
1		*For Group 2 amendment please see section 4.1*
4.1		*** Change to Group 2 vaccinations** A change has been made for group 2 volunteers due to receive their day 28 boost vaccination on or after 2 September 2015, due to a higher frequency of mild respiratory adverse events in this group. Local (skin) and systemic symptoms were as expected and consistent with data from previous studies. These volunteers will not receive the day 28 boost vaccination, but instead will receive a placebo by both intradermal and aerosol inhaled routes to maintain blinding. Additional safety and immunological information will be gained by continuing with day 35 bronchoscopy in these volunteers. Any volunteer not wishing to proceed in this manner will remain in follow up with no vaccination or bronchoscopy.
6.1	Group 2: Intradermal MVA85A 5 x 10^7 pfu (and aerosol inhaled saline placebo) on day 0, followed by homologous boosting with MVA85A 5 x 10^7 pfu aerosol inhaled on day 28 (and intradermal saline placebo)	Group 2: Intradermal MVA85A 5 x 10^7 pfu (and aerosol inhaled saline placebo) on day 0, followed by homologous boosting with MVA85A 5 x 10^7 pfu aerosol inhaled on day 28 (and intradermal saline placebo) OR, following safety amendment (see section 4.1) Intradermal MVA85A 5 x 10^7 pfu (and aerosol inhaled saline placebo) on day 0, followed by aerosol inhaled saline placebo and intradermal saline placebo on day 28

Protocol v 3.0 Substantial Amendment

Section	From	Changed To
4.3		**Restart of enrolment into Group 2** When restarting enrolment into group 2 of the trial, the first participant will be enrolled and no further enrolment will take place until they have received their second bronchoscopy. A

		second volunteer will then be enrolled and once they have received their second bronchoscopy, providing there are no safety concerns, enrolment will continue as normal.
10.2	Subjects will be assigned individual unique trial numbers for identification on all trial records, except where the use of identifiable information is unavoidable (including on GP correspondence, registration documents and consent forms).	Subjects will be assigned individual unique trial numbers for identification on all trial records, except where the use of identifiable information is unavoidable (including on GP correspondence, registration documents, bank details request form and consent forms).
10.4	The study protocol, documentation, data and all other information generated will be held in strict confidence.	The study protocol, documentation, bank details request form, data and all other information generated will be held in strict confidence and in accordance with the Data Protection Act.

Protocol v 2.0 Substantial Amendment

Section	From	Changed To
5.2 Exclusion Criteria	Clinically significant history of skin disorder, allergy, immunodeficiency (including HIV), cancer (except BCC or CIS), cardiovascular disease, gastrointestinal disease, liver disease, renal disease, endocrine disorder, neurological illness, psychiatric disorder, drug or alcohol abuse	Clinically significant history of skin disorder, allergy, atopy, immunodeficiency (including HIV), cancer (except BCC or CIS), cardiovascular disease, gastrointestinal disease, liver disease, renal disease, endocrine disorder, neurological illness, psychiatric disorder, drug or alcohol abuse

**Protocol v 1.3 Non-Substantial Amendment**		
**Section**	**From**	**Changed to**
Key roles		Removal of Dr Sharon Sheehan (left department prior to start of the study)
1. Trial Summary	20ml exploratory immunology on D2 and D30	4ml of Biochemistry and 16ml of exploratory immunology i.e total volume of 20ml remains the same

Protocol v 1.2 Non-Substantial Amendment

Section	From	Changed To
Key roles		Addition of Alice Minhinnick and Morven Wilkie
8.3	All solicited AEs (listed in table 4) and any additional unexpected AEs during the diary card period will be documented in the AE line listing section of the CRF.	During the diary card period, all solicited AEs (listed in table 4) and any additional unexpected AEs are captured in the diary card and at clinic visits (CRF)

Protocol v1.1 Non-Substantial Amendment

**Section**	**From**	**Changed To**
Key Roles	Churchill Hospital	Oxford University Hospitals NHS Trust
1.	Churchill Hospital	Oxford University Hospitals NHS Trust

1. Trial Summary

**Trial Title**	A Phase I trial evaluating mucosal administration of a candidate TB vaccine, MVA85A, as a way to induce potent local cellular immune responses and avoid anti-vector immunity
**Trial Identifier**	TB035
**Clinical Phase**	I
**Active Ingredients of Vaccines**	MVA85A
**Finished Products**	MVA85A
**Dose(s)**	5 x 10^7 pfu
**Route(s)**	Aerosol inhalation by nebuliser and intradermal needle injection in the deltoid region of the arm
**Trial Interventions**	Chest x ray at screening<br>Pulmonary function tests at screening and after vaccination<br>Vaccination by both intradermal and aerosol inhaled routes<br>Venepuncture<br>Bronchoscopy and bronchoalveolar lavage
**Chief Investigator**	Prof Helen McShane
**Trial Centres**	Centre for Clinical Vaccinology and Tropical Medicine (CCVTM), Churchill Hospital, Old Road, Headington, Oxford, OX3 7LE<br>& Oxford Centre for Respiratory Medicine, Oxford University Hospitals NHS Trust
**Planned Trial Period**	Prospective start date for screening August 2013
**Trial Duration**	Estimated at 18 months
**Primary Objectives**	Safety of 5 x 10^7 pfu dose of MVA85A administered by aerosol and compared to the same dose administered intradermally
**Secondary Objectives**	- Characterise mucosal and systemic immunogenicity of viral vector (MVA) and insert (Ag85A) by comprehensive characterisation of humoral and cellular immune responses<br>- Evaluate functional relevance of anti-vector immunity induced by aerosol and systemic immunisation in MVA85A-prime followed by homologous MVA85A-boost administered 4 weeks later
**Planned Sample Size**	36 healthy, BCG vaccinated adult subjects; 12 subjects per treatment arm
**Allocation Method**	Variable block randomisation by sequentially numbered sealed envelopes
**Vaccination Schedule**	Priming vaccination at day 0 (aerosol or intradermal)<br>Homologous boosting vaccination 1 month later (aerosol or intradermal)
**Follow-up Duration**	24 weeks from enrolment
**Blood Sampling Schedule**	Safety blood tests (FBC, coagulation screen and clinical chemistry) as per schedule (table 1)<br>HIV, hepatitis B & hepatitis C antibody blood tests at screening<br>Exploratory immunology tests at screening, as per schedule (table 1)<br>HLA testing at day 0
**Primary Evaluation Criteria**	Actively and passively collected data on adverse events
**Secondary Evaluation Criteria**	Laboratory markers of cell mediated immunity in blood and bronchoalveolar lavage samples

Attendance number	1	2	3	4	5	6	7	8	9	10	11
Timeline (days)*	S	0	2	7	14	28	30	35	42	84	168
Timeline (weeks)*	S	0		1	2	4		5	6	12	24
Time windows (days)			±1	-2+3	±4	±5	±1	-2+3	±4	±14	±21
Inclusion/exclusion criteria	X										
Review contra-indications	X	X		X		X		X			
Informed consent	X										
Medical history	X	(X)	(X)	(X)	(X)	(X)	(X)	(X)	(X)	(X)	(X)
Physical examination	X			X				X			
Vital signs	X	X	X	X	X	X	X	X	X	X	X
Urinalysis	X										
Chest x ray	X										
Pulmonary function tests	X	X	X	X		X	X	X			
β-HCG urine test	X	X		X		X		X			
Vaccinations		X				X					
Bronchoscopy				X				X			
Local & systemic events/reactions		X	X	X	X	X	X	X	X	X	X
Diary cards provided		X				X					
Diary cards collected					X				X		
Biochemistry (4mL)***	X		X	X			X	X			
Haematology (2mL)****	X			X				X			
Coagulation (4mL)	X										
HBV, HCV, HIV (10mL)	X										
HLA typing (4mL)		X									
Exploratory immunology incl ELISPOT (20-60mL**)	X	X	X	X	X	X	X	X	X	X	X
Blood vol (mL)	50	64	20	66	60	60	20	66	60	60	60
Cumulative blood vol (mL)	50	114	134	200	260	320	340	406	466	526	586

S Screening
X Event scheduled to occur
(X) If considered necessary, emphasising any complaint or change in medications
* Timeline is approximate only, as exact timings (± time windows) of visits relate to the actual (not intended) date of the previous visit
** Exploratory immunology blood volume will be a total of 586 ml; these samples will be processed and analysed in the Jenner Laboratories, University of Oxford
*** Sodium, Potassium, Urea, Creatinine, Liver function; these samples will be analysed at OUH
**** Full blood count; these samples will be analysed at OUH
Grey Highlights vaccination day
Blue Highlights bronchoscopy day

Table 1: Schedule of trial procedures

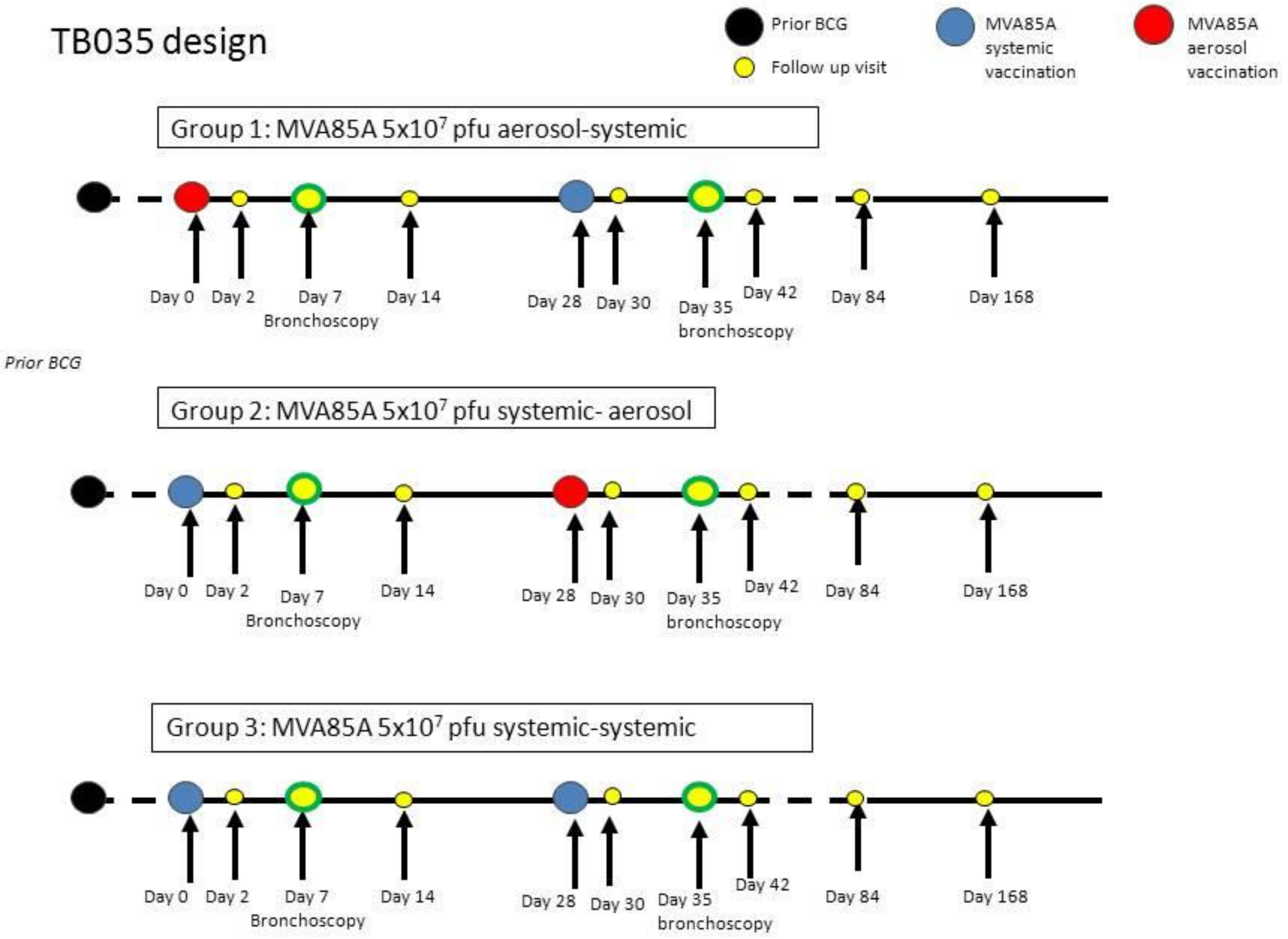

Figure 1. Trial schedule
For Group 2 amendment please see section 4.1

2. Background and Rationale

2.1 Introduction

Mycobacterium tuberculosis (*M. tb*) is a pathogen with worldwide preponderance which infects humans causing tuberculosis (TB), a transmissible disease resulting in very high mortality and morbidity. A third of the world's population is latently infected with *M. tb*, these people carry a 10% lifetime risk of developing active life-threatening disease (1). Worldwide there were an estimated 8.7 million new cases of TB and an estimated 1.4 million deaths associated with TB in 2011 (2) Co-infection with human immunodeficiency virus (HIV) greatly increases risk of TB reactivation and death (3, 4). Diagnosis is challenging and drug treatment can be prolonged, harmful, costly and complex. For these reasons an effective vaccine is a global public health priority.

The Bacille Calmette-Guérin (BCG) vaccine is the only licensed TB vaccine and it has been administered globally to several billion people over a 70 year period (5). Although it does not protect against pulmonary TB in many parts of the world, it is effective in preventing disseminated TB disease including tuberculous meningitis in childhood (4, 6, 7). Recently heterologous "prime-boost" vaccination strategies, in which two different candidate vaccines expressing antigens in common are given weeks or months apart, have generated strong and sustained cellular immune responses correlating with a TB protective effect in preclinical animal models. In such a "prime-boost" strategy, BCG would therefore be an ideal priming vaccine.

MVA85A is a recombinant attenuated Modified Vaccinia virus Ankara expressing the *M. tb* antigen 85A. This antigen is highly conserved in all strains of BCG and *M. tb*, and is immunodominant in both animal and human studies. Developed by the University of Oxford, MVA85A has now been administered to over 2300 human individuals with an excellent safety profile. It has been shown to be highly immunogenic as a booster vaccine in BCG-primed subjects (8). A large phase IIb efficacy trial in BCG-vaccinated infants in South Africa has recently reported no enhancement of BCG-induced protection (9), however, in this trial, the immunogenicity was significantly lower than that seen in adults, and more potent routes of administration are of interest in the field. A phase IIb trial in HIV infected adults in South Africa and Senegal is ongoing (ClinicalTrials.gov reference number NCT01151189).

To date, all new TB vaccine candidates had been administered systemically. However there is data from preclinical animal models with this and other virally vectored vaccines to suggest that immunising the respiratory mucosa may give superior protection against respiratory diseases (10-13).In addition, the aerosol inhaled route of MVA85A delivery could offer practical, tolerability and safety benefits over and above needle-based methods (see below).

A Phase I safety study assessing the safety and feasibility of administering low dose MVA85A by nebuliser direct into the airways has just been completed. To our knowledge there has been no previous clinical study of any vectored vaccine delivered by aerosol except for this completed study in our group.

Enrolment for this trial was completed in November 2012, no vaccine related Serious Adverse Events were reported and aerosol immunisation was well tolerated (Meyer *et al,* personal communication). In that trial 12 volunteers have had the MVA85A as aerosol vaccination- 10 of those had the low dose (1 x 10^7 pfu) and 2 volunteers had the high dose which we would normally give intradermally (1 x 10^8 pfu). The intermediate dose of 5 x 10^7 pfu will be used in this trial for the first time.

Rationale for the aerosol inhaled route for TB vaccination in humans

There are strong reasons for administering a TB vaccine via the airway. Preclinical animal work increasingly indicates that the best protection occurs when the route of immunisation matches the route of infection (10-12). The most common natural route of *M. tb* infection is by inhalation of

aerosolised infectious droplets containing tubercle bacilli leading to the establishment of primary infection in the lung. The lung has a distinct mucosal immune system characterised by bronchus associated lymphoid tissue (BALT) which is well adapted to encounter and process antigens presenting to the respiratory mucosa. Immunising via the airway should therefore have the advantage, over other routes of eliciting protective immune responses in the lung mucosal surface. We know that BCG, administered systemically, protects most effectively against systemic disease in childhood (6, 7). It may follow that airway vaccination is needed to induce effective protection from pulmonary disease.

The inhaled route is a well established route of drug delivery. Aerosolised droplets of bronchodilating, anti-inflammatory, and antimicrobial drugs are administered by inhalation to millions of patients each day. Clinical trials of aerosolised measles vaccines have shown that mass immunisation by the respiratory mucosal route in a developing country is feasible and can induce strong and long lasting immune responses (14-20). There is also valuable experience with aerosolised measles vaccine given to over 4 million schoolchildren in Mexico, where public acceptance was high, costs were low, and there were fewer side effects reported than with subcutaneous vaccination (21). A nasally administered influenza vaccine is licensed in North America (22-24) and others are in clinical trials (25, 26). Furthermore the principle of mucosal surface vaccination is well established as shown by those widely used in clinical practice against polio, typhoid, cholera and rotavirus.

There are numerous perceived advantages of aerosol inhaled vaccination: it is safe, needle-free, well-tolerated, popular, less technically demanding, economically beneficial, and allows self-administration of vaccine or administration by less technically trained community health workers (21). Furthermore, use of non-injectable vaccines reduces the likelihood of unsafe disposal and reuse of syringes in immunisation programmes (27).

A problem with all virally-vectored vaccines including MVA85A is that individuals build up immunity to the virus base (anti-vector immunity) which makes the vaccine less efficient and not suitable for re-use within short time frames. An important potential advantage of mucosal immunisation, in addition to the ones mentioned thus far, is that aerosol vaccination may minimise or avoid induction of systemic anti-vector immunity and allow homologous boosting with the same viral vector; thus minimising cost of vaccine development (28-30). Furthermore, for some vectors, e.g. human Adenovirus serotype-5, up to 80% of adults have antibodies to AdHu5 and this pre-existing immunity significantly limits the utility of this vector (31-34). Our hypothesis is that mucosal immunisation with MVA85A does not result in induction of systemic anti-vector immunity. Recent work in nonhuman-primates by our collaborators at the Health Protection Agency, comparing 2 routes of administration, showed anti-MVA antibody responses are seen in intradermally vaccinated animals but not in animals given MVA85A by aerosol (Figure 2; (35))

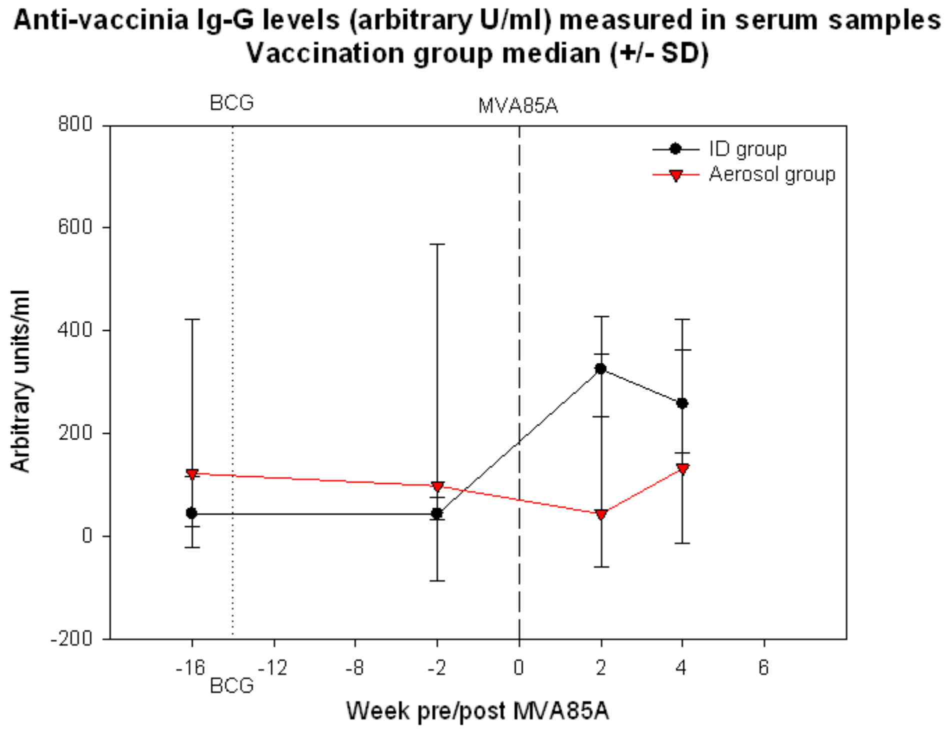

Figure 2: Vector specific IgG response induced by vaccination with MVA85A measured by ELISA. The plot shows the median change from prevaccination level for the BCG prime with MVA85A intradermal boost group with the closed circles and for the BCG prime with MVA85A aerosol boost group with triangles. Error bars are standard deviations from the median *(35).*

This effect of reducing anti-vector responses by mucosal administration has also been demonstrated in mice, rabbits and guinea pigs (12, 36, 37). There is an increased need to find strategies to overcome anti-vector immunity in vaccine development especially for vaccines that will need to be given repeatedly (e.g. seasonal influenza), so if this is also demonstrated in humans, this would be an important development for the field of viral vaccines and respiratory pathogens for which mucosal immunisation may improve efficacy (11, 12).

This project will quantify for the first time the induction of anti-vector immunity (humoral and cell-mediated) by both aerosol and systemic administration of MVA85A. The functional relevance of this anti-vector immunity will be evaluated by a second boost vaccination of MVA85A via a different administration route, one month after the first, priming immunisation. This data will have implications for the TB vaccine field but also for the wider vaccine community and for the development of mucosal vaccination strategies for other respiratory and mucosal pathogens.

Description and pre-clinical experience of MVA and MVA85A

Modified vaccinia virus Ankara (MVA) is a highly attenuated strain of vaccinia virus which cannot replicate in human cells. It is known to be highly immunogenic and is therefore suitable for use as a viral vector in new vaccine development. It has an excellent safety record as it was administered intradermally to approximately 120,000 people during the smallpox eradication campaign (38-41), and has since been used in numerous clinical trials of candidate vaccines against viral, mycobacterial and protozoal infections (42, 43). Meanwhile recombinant MVA vaccines administered by respiratory mucosal and gut mucosal routes have demonstrated strong immune responses in both rodents and non-human primates (44).

Antigen 85A is a highly conserved antigen expressed by *M. tb*, BCG, and all other mycobacterial species sequenced to date. It is a 32-kDa protein, and is an enzyme, mycolyl transferase, which is involved in cell wall biosynthesis (45). Antigen 85A is highly immunodominant in both animal and

human studies (46-48), and protects against *M. tb* challenge in mice, guinea pigs and non-human primates (49, 50). The recombinant MVA85A vaccine incorporates the 1176 base pair gene for antigen 85A into the viral DNA allowing expression of this secreted antigen.

Experiments in mice, guinea pigs, cattle, and non-human primates have shown that a prime-boost schedule of vaccination with BCG followed by MVA85A, either intradermally, intramuscularly or mucosally, can improve protective efficacy against subsequent *M. tb* challenge, compared to BCG alone (10, 50-52). A mouse GLP toxicity study using the intradermal route of administration revealed no differences from PBS-injected controls apart from irritation at the site of administration. An additional mouse GLP toxicology study of aerosol administration revealed no differences from controls given aerosolised PBS.

Clinical studies with MVA85A

Over 2300 subjects have now received MVA85A, of which 12 were by the intramuscular route, 12 by the aerosol route, and the remainder intradermally. 18 Phase I/II clinical studies of MVA85A have been completed, and a further 4 are on-going. These are summarised in Table 2.

Protocol	Phase	Population	Treatment groups[a]	N	Study Status
TB002	Phase I open label non-randomised	Healthy BCG-naïve adults, UK	5×10^7 pfu MVA85A (Days 0 & 21)	14	Completed
GM920	Phase I open label non-randomised	Healthy BCG-naïve or BCG-vaccinated adults, Gambia	5×10^7 pfu MVA85A (Days 0 & 21) BCG naïve<br>5×10^7 pfu MVA85A (Day 0) BCG-vaccinated	11<br>10	Completed
TB004	Phase I open label non-randomised	Healthy BCG-naïve adults, UK	1×10^6 pfu BCG prime (Day 0)<br>5×10^7 pfu MVA85A boost (after 1 month)	10	Completed
TB005	Phase I open label non-randomised	Healthy BCG-vaccinated adults, UK	5×10^7 pfu MVA85A (Day 0)	21	Completed
TB007	Phase I open label non-randomised	Healthy adults latently infected with *M. tb*, UK	5×10^7 pfu MVA85A (Day 0)	12	Completed
TB008	Phase I open label non-randomised	Healthy BCG-naïve or vaccinated adults & adolescents, South Africa	5×10^7 pfu MVA85A (Day 0) adults<br>5×10^7 pfu MVA85A (Day 0) adolescents	24<br>12	Completed
TB009	Phase I open label non-randomised	Healthy BCG-vaccinated adults, UK	1×10^7 pfu MVA85A (Day 0)<br>1×10^8 pfu MVA85A (Day 0)	12<br>12	Completed
TB010	Phase I open label non-randomised	HIV-positive adults, UK	5×10^7 pfu MVA85A (Day 0)<br>1×10^8 pfu MVA85A (Day 0)	10<br>10	Completed
TB011	Phase I open label non-randomised	Adults infected with *M. tb*, HIV, or both, South Africa	5×10^7 pfu MVA85A (Day 0) TB<br>5×10^7 pfu MVA85A (Day 0) HIV<br>5×10^7 pfu MVA85A (Day 0) TB & HIV<br>5×10^7 pfu MVA85A (Day 0) HIV on ARV treatment	12<br>12<br>12<br>12	Completed
TB012	Phase II open label non-randomised	Healthy BCG-vaccinated children & infants, South Africa	<u>Stage 1</u><br>EPI alone (Day 0)<br>2.5×10^7 pfu MVA85A with EPI (Day 0)<br>2.5×10^7 pfu MVA85A alone (Day 0)[b]<br>EPI alone (Day 0)<br>5×10^7 pfu MVA85A with EPI (Day 0)<br>5×10^7 pfu MVA85A alone (Day 0)[b]<br><u>Stage 2</u><br>EPI alone<br>5×10^7 pfu MVA85A with EPI (Day 0)<br>5×10^7 pfu MVA85A alone (Day 0)[b]	 12<br>12<br>12<br>12<br>12<br>12<br><br>47<br>47<br>48	<u>Stage 1</u><br>Completed<br><br><br><br><br><br><u>Stage 2</u><br>Completed
TB014	Phase II open label non-randomised	Healthy BCG-vaccinated children & infants, South Africa	5×10^7 pfu MVA85A (Day 0) children<br>2.5×10^7 pfu MVA85A (Day 0) infants<br>5×10^7 pfu MVA85A (Day 0) infants<br>1×10^8 pfu MVA85A (Day 0) infants<br>Prevenar (variable dose) (Day 0) infants	24<br>36<br>36<br>36<br>36	Completed

TB017	Phase I open label non-randomised	Healthy BCG-vaccinated adults, UK	5 x 10^7 pfu FP85A (Day 0)<br>5 x 10^7 pfu MVA85A (Day 0) then 5 x 10^7 pfu FP85A (Day 28)<br>5 x 10^7 pfu FP85A (Day 0) then 5 x 10^7 pfu MVA85A (Day 28)	12<br>12<br>7	Completed
TB018	Phase I open label non-randomised	Healthy BCG-vaccinated adults, UK	1 x 10^8 pfu MVA85A (Day 0)<br>1 x 10^8 pfu MVA85A (Day 0) then 1 g/kg deuterium labelled glucose (Day 4)<br>1 x 10^8 pfu MVA85A (Day 0) then 1 g/kg deuterium labelled glucose (Day 10)	4<br>4<br>4	Completed
TB019	Phase I open label non-randomised	HIV-infected adults, Senegal	1 x 10^8 pfu MVA85A (Day 0 & 168)<br>1 x 10^8 pfu MVA85A (Day 0 & 168) on ARV treatment	12<br>12	Completed
TB020	Phase II double blinded randomised	Healthy BCG-vaccinated HIV-negative infants, South Africa	1 x 10^8 pfu MVA85A (Day 0)<br>Placebo	1399<br>1398	Completed
TB021	Phase II double blinded randomised	Healthy HIV-infected adults, South Africa & Senegal	1 x 10^8 pfu MVA85A (Day 0)<br>Placebo	325<br>325	In follow up
TB022	Phase I open-label randomised	Healthy BCG-vaccinated adults, UK	Intramuscular 1 x 10^8 pfu MVA85A (Day 0)<br>Intradermal 1 x 10^8 pfu MVA85A (Day 0)	12<br>12	Completed
TB023	Phase I open-label non-randomised	Healthy BCG-naïve or vaccinated adults, UK	1x10^8 pfu MVA85A (Day 0) then BCG challenge (Day 28)<br>BCG: 100 µl ~ 2-8 x 10^5 pfu	24<br>24	Completed
TB026	Phase I randomised blinded	Healthy BCG-vaccinated adults, UK	1x10^7 pfu aerosol inhaled MVA85A with intradermal saline placebo<br>1x10^7pfu intradermal MVA85A injection with aerosol inhaled saline placebo	12<br>12	Completed
TB029	Phase II randomized controlled trial	Infants of HIV infected mothers, South Africa	1 x 10^8 pfu MVA85A or Candin control at birth<br>BCG:0.05mL 1-4 x 10^5 cfu at 8 weeks of age (Infants confirmed HIV uninfected)	170<br>170	Enrolling
TB032	Phase I open-label non-randomised	Healthy BCG-vaccinated adults, UK	1 x 10^{11} vp AERAS-402 (Day 0 and 28) followed by 1 x 10^8 pfu MVA85A (Day 119)<br>1 x 10^{11} vp AERAS-402 (Day 0) followed by 1 x 10^8 pfu MVA85A (Day 56)<br>1 x 10^{11} vp AERAS-402 (Day 0, 28 and 119)	15<br>15<br>15	Enrolling
TB034	Phase I open-label randomised trial	Healthy BCG-vaccinated adults, UK	Starter group: 5 x 10^9 vp ChAdOx1 85A<br>2.5 x 10^{10} vp ChAdOx1 85A<br>2.5 x 10^{10} vp ChAdOx1 85A (D0) followed by 1 x 10^8 pfu MVA85A (D56)	6<br>12<br>12	Enrolling

[a]Intradermal route of administration unless otherwise stated

[b]EPI deferred for 1 week

Table 2. Summary of clinical trials of MVA85A

Safety profile
The typical local reaction as a result of intradermal injection of MVA85A is temporary pain, redness and swelling at the site of the injection with a 5-10 mm central red swollen area and a paler red area ranging from 5-10 mm in diameter which peaks at 48 hours post vaccination. At seven days post vaccination, only the central area of redness remains. This fades over a few weeks and is not usually apparent eight weeks after vaccination. No signs of any Koch type reaction in subjects infected with *M. tb*, has been seen with this or any other candidate vaccine to date.

Systemic reactions to the vaccine are short-lived, and resemble influenza symptoms including fatigue, headache, malaise, feverishness, and muscle aches. These systemic symptoms are typically mild and occur in 50-90% of all subjects.

The preliminary analysis of local and systemic reactions induced by the aerosol route that were reported in the completed Phase I safety study are summarised in section 4.4 “Risks and Benefits”.

Dosing
Dose studies of MVA85A have been performed in a step-wise fashion to minimise the risk of a Koch reaction and the incidence of adverse events. The main dose-finding trial compared boosting doses of 1×10^7 pfu and 1×10^8 pfu to previous trials using boosting doses of 5×10^7 pfu. An intradermal dose of 1×10^8 pfu was found to be significantly more immunogenic than the lower doses without concomitant worsening of the adverse event profile, and has subsequently been adopted as the standard dose in all subsequent trials (53, 54). The aerosol dose administered in trial TB026, a Phase I safety study assessing the safety and feasibility of administering MVA85A by the aerosol route, was 1×10^7 pfu; safety data from this study supports dose escalating to 5×10^7 pfu is the safe and logical next step.

Immunogenicity
When evaluated by various assays of cellular immunology including interferon gamma (IFN-γ) ELISPOT and intracellular cytokine staining assays, MVA85A has induced a strong and sustained cell-mediated immune response which is known to be important for protective immunity (55). A Phase IIb efficacy trial of MVA85A in BCG-vaccinated infants in South Africa has recently been completed and work to identify correlates of protection is now underway (9).

Aerosol delivery devices

Currently the World Health Organisation (WHO) is making a major investment in developing new aerosol devices for portable, low cost vaccine delivery (56). The WHO’s Measles Aerosol Project aims to develop and license respiratory delivery for measles vaccines currently given by injectable methods. The Edmonton Zagreb strain of measles vaccine, a live virus vaccine, has been given by mesh nebuliser with excellent results. Modern devices are small, lightweight, portable, and aerosolise through a mesh to provide small and consistently-sized particles which deliver vaccine to the distal airway mucosa more consistently and precisely than conventional jet mechanisms.

One such mesh nebuliser is the MicroAIR NE-U22 (Omron® Healthcare Limited, Japan) which uses ultrasound to push liquid through a fine metal mesh. This generates an aerosol mist with a particle diameter of about 4 µm. It is in current use with licensed drugs such as bronchodilating and antimicrobial agents and achieves good bioavailability. MVA particles are typically around 400 nm in size and can therefore be aerosolised with minimal damage.

We demonstrated adequate delivery of our vaccine, MVA85A, by this device in preliminary plaque assays. Using a nebuliser adapted for rodent administration, we performed GLP standard toxicology studies of aerosolised MVA85A (1×10^8 pfu) in BalbC mice, demonstrating satisfactory safety and systemic immunogenicity. We further administered aerosolised MVA85A (1×10^8 pfu) by the MicroAIR NE-U22 mesh nebuliser to eight macaques, showing a good safety profile and satisfactory delivery by inhaled route (unpublished data).

Bronchoscopy and bronchoalveolar sampling

Protection from TB induced by intra-pulmonary vaccination correlates with cellular immune responses detected in the bronchoalveolar lavage (BAL) samples of immunised mice (57). In macaques receiving aerosolised MVA85A we detected significant levels of antigen-specific cellular immune responses in bronchoalveolar lavage samples (35). These pre-clinical findings provide the rationale for wishing to obtain BAL samples from our clinical trial subjects.

BAL samples are obtained by performing a bronchoscopy, which is a widely and safely used procedure in clinical research studies involving both healthy subjects and subjects with respiratory diseases such as asthma (58, 59). Clinical guidelines for performing investigative bronchoscopy in research studies are well established (60). The short procedure involves the insertion of a narrow flexible fibre-optic tube into the airway under light intravenous sedation. Under direct vision saline is delivered to a section of lung mucosa and then recollected by suction. In clinical trial TB026 bronchoscopies were well tolerated by volunteers.

Bronchoscopy is an essential component of this clinical trial because BAL samples are the ideal method to analyse the lung mucosal cellular immune responses which we intend to induce by vaccination (61). Clinical trials of intranasal vaccine candidates for other infectious diseases have analysed humoral immunity within nasal lavage, but a cellular immune response is essential for protective immunity against *M. tb* (62). It is unlikely that administering a vaccine intranasally in humans would result in the induction of a significant cell mediated immune response in the lower airways. Nasal mucosal samples are unlikely to provide adequate cellular samples as nasal lavage generally yields insufficient and variable numbers of cells (63). Furthermore as the vaccine is nebulised and inhaled orally we do not expect it to reach the nasal mucosa.

2.2 Hypothesis

Previous studies have demonstrated that a 3 week interval between homologous MVA85A–MVA85A administered systemically does not enhance responses (8). We hypothesise that responses will be boosted in the aerosol–systemic and systemic-aerosol group but not in the systemic–systemic group.

3. Objectives and outcome measures/ Endpoints

Objectives	Outcome Measures/Endpoints
**Primary Objective**<br>To evaluate the safety of 5 x 10^7 pfu dose of MVA85A administered by aerosol and compared to the same dose administered intradermally.	Safety of 5 x 10^7 pfu dose MVA85A administered by the inhaled route through actively and passively collected data on adverse events.
**Secondary Objectives**<br>To characterise mucosal and systemic immunogenicity of viral vector (MVA) and insert (Ag85A) by comprehensive characterisation of humoral and cellular immune responses<br><br>Evaluate functional relevance of anti-vector immunity induced by aerosol and systemic immunisation in MVA85A-prime followed by homologous MVA85A-boost administered 4 weeks after the MVA85A-prime	1. Induction of humoral and cell-mediated immunity to MVA by aerosol and systemic immunisation<br>2. Induction of cell-mediated and humoral immunity to Ag85A by aerosol and systemic immunisation<br>3. Effect of a boosting immunisation one month after prime on humoral and cell-mediated immunity to insert (Ag85A) and vector (MVA)

4. TRIAL DESIGN

4.1 Trial Overview

This is a Phase I clinical trial where subjects are randomised to 3 different groups of homologous MVA85A boosts by either intradermal or aerosol route of vaccination to evaluate safety and immunogenicity of homologous boosting by heterologous routes of immunisation.

Trial groups

This will be a Phase I clinical trial with a standard dose of MVA85A (pfu) in 3 groups of 12 volunteers with 3 different route permutations for boosting (see Table 3).

The overall investigational approach with our MVA85A trials is to develop an effective prime-boost vaccination strategy to prevent TB infection, with BCG as the priming vaccine.

This Phase I trial will have 3 treatment arms (homologous):

Group	Route of priming MVA85A	Bronchoscopy 1 week later	Route of boosting MVA85A	Bronchoscopy 1 week later
1	Aerosol	Yes	Systemic	Yes
2	Systemic	Yes	Aerosol*	Yes
3	Systemic	Yes	Systemic	Yes

Table 3: Route of immunisations and timing of bronchoscopy

*** Change to Group 2 vaccinations**
A change has been made for group 2 volunteers due to receive their day 28 boost vaccination on or after 2 September 2015, due to a higher frequency of mild respiratory adverse events in this group. Local (skin) and systemic symptoms were as expected and consistent with data from previous studies. These volunteers will not receive the day 28 boost vaccination, but instead will receive a placebo by both intradermal and aerosol inhaled routes to maintain blinding. Additional safety and immunological information will be gained by continuing with day 35 bronchoscopy in these volunteers. Any volunteer not wishing to proceed in this manner will remain in follow up with no vaccination or bronchoscopy.

As BCG is protective in childhood, it is routinely given at birth in TB endemic countries but is also in widespread use in children and adolescents in much of the rest of the world. Therefore, as in previous trials of intradermal MVA85A, we propose to vaccinate BCG-primed subjects. These will be healthy men and women aged 18-55 years who have been previously vaccinated with BCG.

All subjects will have received BCG vaccination at least six months prior to enrolment. MVA85A (5 $x10^7$ pfu) or saline placebo will be administered by systemic (intradermal) or aerosol (Omron jet nebuliser) route (paired placebo design). The interval between the two vaccinations is one month.

In order to minimise the risk from bronchoscopy, subjects with a history or evidence of lung disease will be excluded. This will be determined by our standard screening procedures (history, examination, urine analysis, and blood tests), and, in addition, a chest x ray and pulmonary function testing.

The peak effector T-cell response in blood is ~7 days post-vaccination with MVA85A. Pulmonary mucosal responses are expected to follow a similar time course and a preclinical study has demonstrated this in non-human primates (35). Thus bronchoscopy and broncholaveolar lavage will be performed seven days after each vaccination.

4.2 Randomisation

In order to reduce bias and the influence of any difference in the baseline characteristics between the three groups, we have chosen to randomise the allocation of subjects. This aims to achieve a balance between the three groups in any important confounding factors such as levels of pre-existing antimycobacterial immunity (influenced by, for example, country of origin and length of time elapsed since prior BCG vaccination). This will be performed by variable block randomisation using sequentially numbered sealed envelopes, prepared by an independent colleague at the Centre for Statistics in Medicine, to ensure allocation concealment. Volunteers are blinded as they will receive a paired placebo at the same time of vaccination. The bronchoscopist will be blinded to the route of vaccine administration. This is intended to eliminate any bias in the reporting of the appearance of the lung mucosa and extent of airway inflammation. In addition, the postdoctoral researcher in the laboratory overseeing the immunological analyses will also be blinded to reduce any bias that could be introduced at the sample processing stage. All samples will be anonymised.

4.3 Vaccine dosage

Trials of MVA85A to date have established 1 x 10^8 pfu as the optimal dose for intradermal injection in adults. There are no published studies of dose-response via the mucosal route of vaccination. Although the fractional delivered dose of aerosolised viral vectored vaccines to the mucosal surface may be considerably less than 25%, there is evidence that strong mucosal and systemic immune responses, comparable to the injectable route, are nevertheless achieved (64, 65). The first aerosol safety study (TB026) was completed using 1 x 10^7 pfu MVA85A with no severe or serious adverse events.

In this trial we will dose escalate to a medium dose to further evaluate safety and immunogenicity. The first volunteer in the study will receive a dose of 5 x 10^7 pfu MVA85A by the aerosol route. This volunteer will be vaccinated ahead of any other volunteers and the profile of adverse events will be examined. No other volunteers will be vaccinated until at least 48 hours has elapsed following the first volunteer being vaccinated. The Chief Investigator will then decide whether it is safe to proceed to vaccinate the remaining volunteers post review of this safety data. The Chair of the Local Safety Committee will be informed if an untoward adverse event profile occurs that differs from past experience with MVA85A.

Restart of enrolment into Group 2

When restarting enrolment into group 2 of the trial, the first participant will be enrolled, and no further enrolment will take place until they have received their second bronchoscopy. A second volunteer will then be enrolled and once they have received their second bronchoscopy, providing there are no safety concerns, enrolment will continue as normal.

Time points

Prior BCG – trial enrolment time window

The specified period for prior receipt of BCG is such that no less than six months must have elapsed between prior BCG vaccination and subsequent trial enrolment. This is to allow for the peak BCG-induced immune response to have occurred, which is usually around 2-4 weeks post BCG vaccination.

Screening – trial enrolment time window

Enrolment should take place no longer than 120 days following the date of screening appointment. If more than 120 days elapse, the screening visit should be repeated in full prior to enrolment in order to minimise the risk to participants of any new unidentified health problems having arisen during that period.

Follow up period

The follow up period will be five months from the last vaccination (i.e. 24 weeks from enrolment), in accordance with findings from previous trials in which adequate safety data and reliable markers of immunogenicity have been obtained in this time interval.

4.4 Risks and Benefits

Potential risks

The potential risks to participants in this trial include risks associated with:

1. Venepuncture and intravenous cannulation

Localised bruising and discomfort can occur at the site of venepuncture. Infrequently fainting may occur. The total volume of blood drawn over a six month period will be 586 mL which should not compromise the health of these healthy individuals.

An intravenous cannula is routinely inserted into a peripheral (usually forearm) vein prior to bronchoscopy to administer intravenous sedation, and removed once the procedure is completed. The risks of cannulation are identical to those associated with venepuncture. Additionally cannulation carries a small risk of soft tissue infection which will be minimised by an aseptic insertion technique and is easily recognisable and treatable. The short duration of cannulation (a few hours) further minimises this risk.

2. Vaccination

MVA85A has undergone thorough pre-clinical testing and has now been administered to over 2300 healthy human individuals with no serious adverse events. The potential known adverse events associated with vaccination are:

Local reaction from intradermal vaccination
The typical local reaction as a result of intradermal injection is temporary pain, redness and swelling at the site of the injection with a 5-10 mm central red swollen area and a paler red area ranging from 5-10 mm in diameter which peaks at 48 hours post vaccination. At seven days post vaccination, only the central area of redness remains. This fades over a few weeks and is not usually apparent eight weeks after vaccination. Scaling occurs as part of the healing process, and itching is possible.

Local reaction from aerosol inhaled vaccination
Enrolment for the first ever trial administering a virally vectored vaccine by the aerosol route has just been completed in our group. The trial was single blinded paired placebo, so all volunteers received either an intradermal vaccine with saline by aerosol or vice versa. There was no significant difference between systemic adverse events, respiratory adverse events or pulmonary function tests between the two groups.

Constitutional influenza-like symptoms such as fatigue, headache, malaise, feverishness, and muscle aches can occur with any vaccination lasting for 2-3 days. In the case of MVA85A, fatigue and headache are most common, occurring in 50-90% of subjects, and these are nearly always mild. Presyncopal and syncopal episodes may occur at the time of vaccination which rapidly resolve. As with any other vaccine, temporary ascending paralysis (the Guillain-Barré syndrome, GBS) or immune mediated reactions that can lead to organ damage may occur. For influenza vaccines an excess of approximately 1 GBS case per million persons immunized has been observed. No cases were observed in people under 45 years of age. However, this has never been seen with the MVA85A vaccine or vaccines containing any of its components.

Allergic reactions
Allergic reactions from mild to severe may occur in response to any constituent of a vaccine preparation. Anaphylaxis is extremely rare but can occur.

3. Bronchoscopy

Bronchoscopy is a widely and safely used investigative procedure in clinical research studies involving both healthy subjects and subjects with respiratory conditions such as asthma and interstitial lung disease (58, 59). Clinical guidelines for performing investigative bronchoscopy in research studies are well established (60). In 98% of cases subjects have no memory of the procedure.

The bronchoscopies will be carried out in a dedicated NHS bronchoscopy suite with an excellent safety record by highly skilled and experienced respiratory physicians. Intravenous sedation and local anaesthesia are administered prior to bronchoscopy to reduce discomfort, facilitate the procedure, and remove memory of the event. Trained, experienced staff and facilities for resuscitation and drugs for reversal of sedation will be available. No transbronchial or endobronchial biopsies will be taken. To further minimise risk, subjects will be excluded if they have an abnormal chest x ray, a significant smoking history, or any evidence of lung disease, including asthma (as defined by: a clinical diagnosis of asthma; prescription of asthma medication; airflow obstruction on spirometry; history of nocturnal or exercise-induced wheeze).

The risks of bronchoscopy are: infection secondary to bronchoalveolar lavage, bleeding, and respiratory depression secondary to sedation:

- Bronchoalveolar lavage is a routine procedure in investigative bronchoscopy and carries minimal bleeding risk. The risk of infection or febrile reactions will be minimised by full bronchoscope asepsis and superflush.

- Respiratory depression secondary to sedation is rare. No rescue medication for oversedation has been required in a 3 year audit period and sedation with midazolam and fentanyl removed memory of the event in over 98% of subjects (internal audit data).

- Allergic reactions from mild to severe may occur in response to any constituent of the local anaesthetic or sedative agents. Anaphylaxis is extremely rare but can occur.

The Summary of Product Characteristics (SmPC) for the local anaesthetic and sedative agents contain full details of the indications and side effects of these licensed medications.

Bronchoscopies were well tolerated in the volunteers of the recently completed Phase I trial TB026. No serious adverse events were reported during or as a result of bronchoscopy.

4. Chest X-Ray

A chest x-ray is a painless routine radiological investigation which exposes subjects to approximately 0.02 milliSieverts of radiation, equivalent to around three days of natural background radiation. The additional risk of cancer due to one chest x ray is extremely small, approximately 1 in 900,000.

5. Pulmonary Function Tests

Vigorous respiratory maneouvres such as forced expiration through the spirometer can occasionally lead to coughing or lightheadedness, but these symptoms are mild and rapidly self-limiting.

Known potential benefits

Subjects are not expected to benefit directly from participation in this trial. Subjects will gain some information about their general health as a result of the screening history, examination, blood tests, urine tests, chest x-ray, and pulmonary function tests. They may also gain health information from the bronchoscopy. It is hoped that their contribution will enable the development of a safe and successful vaccine for TB and further our knowledge about mucosal vaccines.

4.5 Data Collection

The bronchoscopic procedure is not being assessed for safety as it is a standard medical technique with a known profile of expected risks as described in section 4.4. Vaccination safety data for both routes will be collected both actively and passively as follows:

- Information about local and systemic adverse events will be collected at each visit by medical history, examination and measurement of the injection site, pulmonary function testing, bronchoscopic examination of the lungs, and the use of diary cards.
- Vital signs will be recorded at each visit.
- Safety blood tests (FBC, LFT, U&E) will be collected at screening and day 7.

This information will be entered into the CRF contemporaneously at each visit. Results of laboratory investigations and immunological data will be entered into the CRF. The CRFs will be the source data for this trial.

4.6 Duration of Study

Volunteers will all be followed up to week 24, with clinic visits and blood test as outlined in table 1.

4.7 Definition of start and end of the trial

The start of trial is defined as the date of the first vaccination of the first volunteer. The end of trial is the date of the last visit of the last volunteer.

4.8 Discontinuation of the trial

The trial will be discontinued in the event of any of the following:

- New scientific information is published to indicate that subjects in the trial are being exposed to undue risks as a result of administration of the IMP, or as a result of the trial procedures or follow-up schedule.
- Serious concerns about the safety of the IMP arise as a result of one or more vaccine related SAE(s) occurring in the subjects enrolled in this or any other on-going trial of the MVA85A vaccine.
- For any other reason at the discretion of the Chief Investigator.

5 Selection and Withdrawal of Subjects

5.1 Inclusion Criteria

Subjects must meet all of the following criteria to enter the trial:

- Healthy adult aged 18-55 years
- Resident in or near Oxford for the duration of the trial period
- No relevant findings in medical history or on physical examination
- Confirmation of prior vaccination with BCG not less than 6 months prior to projected trial vaccination date (by visible BCG scar on examination or written documentation)
- Allow the Investigators to discuss the individual's medical history with their GP

- Use effective contraception for the duration of the trial period (females only)
- Refrain from blood donation during the trial
- Give written informed consent
- Allow the Investigator to register subject details with a confidential database to prevent concurrent entry into clinical trials
- Able and willing (in the Investigator's opinion) to comply with all the trial requirements

5.2 Exclusion Criteria

Subjects must meet none of the following criteria to enter the trial:

- Any respiratory disease, including asthma
- Current smoker
- Clinically significant abnormality on screening chest x-ray[a]
- Clinically significant abnormality of pulmonary function tests[a]
- Any nasal, pharyngeal, or laryngeal finding which precludes bronchoscopy
- Current use of any medication taken through the nasal or inhaled route including cocaine or other recreational drugs
- Laboratory evidence at screening of latent *M. tb* infection as indicated by a positive ELISPOT response to ESAT6 or CFP10 antigens[b]
- Clinical, radiological, or laboratory evidence of current active TB disease[a]
- Previous vaccination with candidate vaccine MVA85A or candidate vaccine FP85A or any other recombinant MVA vaccine
- Clinically significant history of skin disorder, allergy, atopy, immunodeficiency (including HIV), cancer (except BCC or CIS), cardiovascular disease, gastrointestinal disease, liver disease, renal disease, endocrine disorder, neurological illness, psychiatric disorder, drug or alcohol abuse
- History of serious psychiatric condition
- Concurrent oral or systemic steroid medication or the concurrent use of other immunosuppressive agents
- History of anaphylaxis to vaccination or any allergy likely to be exacerbated by any component of the trial vaccine, sedative drugs, or any local or general anaesthetic agents
- Any abnormality of screening blood or urine tests that is deemed to be clinically significant or that may compromise the safety of the subject in the trial[a]
- Positive HBsAg, HCV or HIV antibodies[a]
- Female currently lactating, confirmed pregnancy or intention to become pregnant during trial period
- Use of an investigational medicinal product or non-registered drug, live vaccine, or medical device other than the trial vaccine for 30 days prior to dosing with the trial vaccine, or planned use during the trial period
- Administration of immunoglobulins and/or any blood products within the three months preceding the planned trial vaccination date
- Any other significant disease, disorder, or finding, which, in the opinion of the Investigator, may either put the subject at risk or may influence the result of the trial or may affect the subject's ability to participate in the trial

[a]Subjects who are excluded from the trial because they have been discovered during screening procedures to be suffering from a previously undiagnosed condition thought to require further medical attention will be referred appropriately to their GP or an NHS specialist service for further investigation and treatment.

[b]Subjects discovered to have evidence of latent *M. tb* infection as defined by a positive ELISPOT test will be referred for a plain chest x-ray and reviewed with the TB nurse specialists and considered for chemoprophylaxis. If there is any evidence of active TB disease either on clinical or radiological grounds, further investigation and treatment will be offered under the supervision of a consultant physician in respiratory or infectious diseases.

5.3 Discontinuation/Withdrawal of Participants from Trial Treatment

Subjects may be withdrawn from the trial early:

- By withdrawing voluntarily
- On the decision of the Chief Investigator
- On the advice of the LSC

The Chief Investigator may withdraw the subject for any of the following reasons:

- Confirmed pregnancy during the trial
- Any adverse event which results in inability to comply with trial procedures
- Ineligibility either arising during the trial or retrospectively (having been overlooked at screening)
- Significant protocol deviation
- Subject non-compliance with trial requirements
- Loss to follow up (applies to a subject who does not return for protocol trial visits, is not reachable by telephone or other means of communication and/or is not able to be located)

The reason for withdrawal will be recorded in the CRF. Subjects withdrawn from the trial may be replaced on the decision of the Investigator. If the subject is withdrawn due to an AE, the Investigator will arrange for appropriate specialist management or follow up visits or telephone calls until the AE has resolved or stabilised. The extent of follow up after premature discontinuation will be determined by the Investigator but will be at least for the whole trial period, and if pregnant, until pregnancy outcome.

Subjects found during bronchoscopy to have a previously undiagnosed condition thought to require further medical attention will be referred appropriately to their GP or an NHS specialist service for further investigation and treatment. If a volunteer withdraws from the study, blood samples collected before their withdrawal from the trial will be used/ stored unless the volunteer specifically requests otherwise

5.4 Vaccination postponement Criteria

Vaccination will not proceed on the scheduled day in any of the following situations:

- The subject has a current or recent upper respiratory tract infection, unless they have been symptom-free for at least one week
- The subject has a positive pregnancy test
- The subject has a temperature > 37.5^{o}C
- The Investigator judges the subject to have an acute moderate or severe illness (whether febrile or not)
- The subject has received a live non-trial vaccine within the preceding three weeks
- The Investigator has any other concern that vaccination may not be in the subject's best interests

In this case the subject may be vaccinated at a later date or withdrawn from the trial at the discretion of the Investigator.

5.5 Bronchoscopy postponement criteria

Bronchoscopy will not proceed on the scheduled day in any of the following situations:

- The subject has a temperature > 37.5^{o}C
- The subject has a positive pregnancy test
- The Investigator judges the subject to have an acute moderate or severe illness (whether febrile or not)
- The Investigator has any other concern that bronchoscopy may not be in the subject's best interests

In the event of postponement the bronchoscopy will be rescheduled where possible up to a 3 week window.

6 TRIAL VACCINE, MEDICINES & DEVICES

6.1 Candidate TB vaccine MVA85A

Description

The MVA85A vaccine consists of the attenuated vaccinia virus MVA vector with a 1176 base-pair insert, which is almost the complete *M. tb* gene for Ag85A, with the tissue plasminogen activator (TPA) signal sequence preceding the N terminus and a monoclonal antibody tag (pk) at the C terminus. Expression of the antigen 85A DNA sequence is regulated by the vaccinia P7.5 early/late promoter. MVA85A is manufactured under Good Manufacturing Practice conditions by IDT Biologika GmbH (IDT), Germany.

Formulation

MVA85A lot 0050811 is supplied as a sterile liquid in glass vials. Each vial contains 400 µL of vaccine at a concentration of 8.4 x 10^{8}pfu/mL in 10mM Tris buffer 140 mM NaCl, pH 7.7. The dose of MVA85A to be used in this trial will be 5 x 10^{7} pfu (60 µL) in a single intradermal injection into the deltoid region of the upper arm or diluted in saline and administered by aerosol administration. Therefore two doses may be obtained from a single vial, enabling two volunteers to be vaccinated within the same time period.

Vaccine Supply and Product storage

MVA85A will be shipped from IDT directly to the Clinical Biomanufacturing Facility (CBF) on dry ice and in the presence of a temperature logger. The vaccine will be certified and labelled for use in trial TB035 by a qualified person (QP) at the University of Oxford.

The vaccine will be stored between –70°C and –90°C, in a locked freezer, at the University of Oxford, Churchill Hospital

Dispensing and administration

All movements of vials of the trial vaccine between IDT and the University of Oxford and in or out of the locked freezer will be documented. Vaccine accountability, storage, shipment and handling will be in accordance with local SOPs and other relevant local forms. On vaccination day, vaccines will be allowed to thaw to room temperature and will be administered within 1 hour. The vaccine will be administered intradermally over the deltoid region of each upper arm, according to the site-specific SOP. The vaccine will administered by aerosol inhalation according to the site-specific SOP. Subjects will stay in the unit for 60 minutes (±10 minutes) after vaccination. During the administration of the

vaccine, monitoring equipment, oxygen, medicines including bronchodilators and resuscitation equipment will be immediately available for the management of anaphylaxis and bronchospasm according to the site SOP.

In order to minimise dissemination of the recombinant vectored vaccine virus into the environment, a number of measures will be instituted during and following vaccination:

- The intradermal injection site will be covered with a dressing after vaccination to absorb any virus that may leak out through the needle track. The dressing will be removed from the injection site after 30 minutes.
- The aerosol inhaled vaccination will take place in a closed clinic room and not opened until 10 minutes after the end of nebulisation.
- The dilution process will be carried out in a sealed vial in accordance with the site-specific SOP.
- During aerosol inhalation the Investigator will wear appropriate protective clothing as agreed with the GMSC.
- All disposable items including nebuliser parts, needles, vials, dressings, and protective clothing will be disposed of as GMO waste by autoclaving, in accordance with the current approved SOPs and standard UK practice.
- The hard surfaces within close range (1-2 metres) of aerosol vaccination will be cleaned and disinfected in accordance with GMSC recommendations.
- Non-disposable parts of the MicroAIR NE-U22 nebuliser will be cleaned and disinfected in accordance with the device documentation and GMSC recommendations.

Trial regimen

Group 1: Aerosol inhaled MVA85A 5 x 10^7 pfu (and intradermal saline placebo) on day 0, followed by homologous boosting with MVA85A 5 x 10^7 pfu intradermal on day 28 (and aerosol inhaled saline placebo)
Group 2: Intradermal MVA85A 5 x 10^7 pfu (and aerosol inhaled saline placebo) on day 0, followed by homologous boosting with MVA85A 5 x 10^7 pfu aerosol inhaled on day 28 (and intradermal saline placebo) OR, following safety amendment (see section 4.1)
Intradermal MVA85A 5 x 10^7 pfu (and aerosol inhaled saline placebo) on day 0, followed by aerosol inhaled saline placebo and intradermal saline placebo on day 28
Group3: Intradermal MVA85A 5 x 10^7 pfu (and aerosol inhaled saline placebo) on day 0, followed by homologous boosting with MVA85A 5 x 10^7 pfu intradermal on day 28 (and aerosol inhaled saline placebo)

Labelling and packaging

The vaccine, including intended route of administration, will be QP certified and labelled with Annex 13 compliant labels for trial TB035 at the CBF. The vials will be packaged into labelled cardboard boxes at the CBF.

6.2 Saline

Sterile saline (0.9%) will be used for dilution and placebo doses. The volume of saline used as placebo will be identical to the vaccine volume by the same route. For volunteers receiving the aerosol vaccination we will administer 5 x 10^7 pfu of vaccine made up to 1ml with saline and at the same time a paired placebo of 135 microlitres of normal saline for intradermal injection. For volunteers receiving the vaccine intradermally we will administer 5 x 10^7 pfu of vaccine made up with normal saline to a vaccine volume of 135 microlitres and at the same time a paired placebo of 1ml of saline inhaled. We require 60ul of MVA85A vaccine to give a dose of 5 x 10^7 pfu. For the aerosol vaccine the total volume is 1ml, i.e. ~940 microlitres of saline and 60 microlitres of vaccine. The volume for intradermal injection is 135 microlitres, i.e. ~75 microlitres of saline with 60microlitires of vaccine.

6.3 MicroAIR NE-U22

This is an approved electromedical device, CE0197, EAN code 40 15672 10142 1. Information about the specifications, usage, and maintenance of the nebuliser device can be found in the device documentation (66). This is the same device used for the study TB026.

6.4 Sedative & anaesthetic agents for bronchoscopy

Fentanyl is a licensed opioid used routinely to provide analgesia and sedation during medical procedures. It will be stored, dispensed and administered in accordance with standard NHS procedures and the Summary of Product Characteristics (SmPC).

Midazolam is a licensed benzodiazepine used routinely to provide sedation and amnesia during medical procedures. It will be stored, dispensed and administered in accordance with standard NHS procedures and the Summary of Product Characteristics (SmPC).

Lignocaine is a licensed local anaesthetic used routinely during medical procedures. It will be stored, dispensed and administered in accordance with standard NHS procedures and the Summary of Product Characteristics (SmPC).

Other licensed drugs may be used during bronchoscopy at the discretion of the Investigators.

7 TRIAL SCHEDULE

7.1 Recruitment

Subjects may be recruited by use of an advertisement formally approved by the ethics committee and distributed or posted in the following places:

- In public places (including NHS hospitals and university buildings) with the agreement of the owner or proprietor
- In newspapers or other literature for circulation
- On radio via announcements
- On a website operated by our group or with the agreement of the owner or operator
- As a post on a Twitter account owned and operated by our group
- By e-mail distribution to a group or list only with the express agreement of the network administrator or with equivalent authorization
- On stalls or stands at exhibitions or fairs
- Via presentations (e.g. presentations at lectures or invited seminars)
- Direct mail-out. This will involve obtaining names and addresses of adults via the most recent Electoral Roll (with the contact details of individuals who have indicated that they do not wish to receive postal mail-shots already removed). The company providing this service is registered under the Data Protection Act 1998. Investigators would not be given dates of birth or ages of individuals but the list supplied would only contain names of those aged between 18-55 years (as per the inclusion criteria).

• Oxford Vaccine Centre databases: We may contact individuals from databases of groups within the CCVTM, (including the Oxford Vaccine Centre database) of previous trial participants who have expressed an interest in receiving information about all future studies for which they may be eligible.

A copy of intended advertising will be submitted with the initial application for ethical approval and any significant changes to this advert will be submitted as an amendment to the ethics committee for approval before use.

7.2 Screening

Subjects will be offered a screening visit if they express an interest in participating in the trial. They will be assigned a subject identifier number and provided with and asked to read the subject information sheet at least 24 hours prior to attending for screening. At the screening visit the general process of screening will be outlined and all questions about the screening process and the trial answered. The following general principles will be emphasised:

- Participation in the trial is entirely voluntary
- Refusal to participate involves no penalty or loss of medical benefits
- The subject may withdraw from the trial at any time
- The subject is free to ask questions at any time to allow him or her to understand the purpose of the trial and the procedures involved
- The trial involves administration of a unlicensed vaccine and a bronchoscopy
- There is no direct benefit from participating
- The subject’s GP will be contacted to corroborate their medical history if possible

The aims of the trial and all tests to be carried out will be explained by the Investigator or the Clinical Research Nurse. The subject will be given the opportunity to ask about details of the trial, and will then have time to consider whether or not to participate. If they do decide to participate, they will sign and personally date two copies of the consent form, one for them to take away and keep, and one for the Investigator. These forms will also be signed and dated by the Investigator or Clinical Research Nurse.

Having agreed to undergo screening, a baseline medical history (including concomitant medication) and physical examination will be performed. Inclusion and exclusion criteria will be checked using a tabulated format. Demographic and occupational data relating to risk of prior TB exposure will be collected. Vital signs will be checked. Subjects will be counselled by one of the Investigators for HIV, Hepatitis B and Hepatitis C testing. Blood tests as specified in the schedule of procedures will be taken. Urinalysis and a pregnancy test will be performed. Chest x ray and pulmonary function testing will be performed on the same day if possible, or scheduled on a separate visit. The total duration of the screening visit will be approximately two hours.

Subjects will be informed that there may be leftover samples of their blood (after all testing for this trial is completed), and that such samples may be stored up to a maximum of 15 years for possible future research. Samples will be stored in liquid nitrogen storage at the Jenner Institute, University of Oxford under the approved study code “Lab T001: Finding an Immune Correlate of Protection against Tuberculosis' (Berkshire REC Reference Number: 06/Q1602/146). Subjects will be able to decide if they will permit such future use of any leftover samples. If they elect not to permit this, all of those leftover samples will be discarded after the required period of storage to meet Good Clinical Practice (GCP) and regulatory requirements.

The subject’s general practitioner will be contacted with the written permission of the subject after satisfactory screening as notification that the subject is taking part in the trial and to ascertain any significant medical history.

7.3 Enrolment

Subjects who fulfil the screening criteria, satisfy all the inclusion criteria, and meet none of the exclusion criteria will be enrolled into the trial and their visits scheduled in the calendar. The ongoing eligibility of the subject will be reviewed on the day of enrolment and any new events, medications, or changes to the screening documents recorded. Subjects will be enrolled on the vaccination visit (day 0). The vaccination visit will last approximately two hours, which includes a 60 minute follow up period in the clinic for observation after vaccination. The bronchoscopy visit (day 7) will last several hours. All other pre-vaccination and follow up visits will last approximately 15 to 30 minutes.

Enrolment should take place no longer than 120 days following the date of screening appointment. If more than 120 days elapse, the screening visit should be repeated in full prior to enrolment.

7.4 Safety Review

There will be a short visit for safety review 2 days following vaccination at which the diary card will be reviewed, pulmonary function test repeated and blood tests repeated.

7.5 Follow-up

The maximum trial duration will be around 6 months after the date of enrolment. Procedures will be performed at the time points indicated in the schedule of procedures (Table 1). Additional procedures or laboratory tests may be performed, at the discretion of the investigators if deemed clinically necessary.

8. Safety Reporting

8.1 Definitions

Adverse Event (AE)	Any untoward medical occurrence in a participant to whom a medicinal product has been administered, including occurrences which are not necessarily caused by or related to that product.
Adverse Reaction (AR)	An untoward and unintended response in a participant to an investigational medicinal product which is related to any dose administered to that participant.<br>The phrase "response to an investigational medicinal product" means that a causal relationship between a trial medication and an AE is at least a reasonable possibility, i.e. the relationship cannot be ruled out.<br>All cases judged by either the reporting medically qualified professional or the Sponsor as having a reasonable suspected causal relationship to the trial medication qualify as adverse reactions.
Serious Adverse Event (SAE)	A serious adverse event is any untoward medical occurrence that:<br>• results in death<br>• is life-threatening<br>• requires inpatient hospitalisation or prolongation of existing hospitalisation

	• results in persistent or significant disability/incapacity<br>• consists of a congenital anomaly or birth defect.<br><br>Other 'important medical events' may also be considered serious if they jeopardise the participant or require an intervention to prevent one of the above consequences.<br><br>NOTE: The term "life-threatening" in the definition of "serious" refers to an event in which the participant was at risk of death at the time of the event; it does not refer to an event which hypothetically might have caused death if it were more severe.
Serious Adverse Reaction (SAR)	An adverse event that is both serious and, in the opinion of the reporting Investigator, believed with reasonable probability to be due to one of the trial treatments, based on the information provided.
Suspected Unexpected Serious Adverse Reaction (SUSAR)	A serious adverse reaction, the nature and severity of which is not consistent with the information about the medicinal product in question set out:<br><br>• in the case of a product with a marketing authorisation, in the summary of product characteristics (SmPC) for that product<br>• in the case of any other investigational medicinal product, in the investigator's brochure (IB) relating to the trial in question.

NB: To avoid confusion or misunderstanding of the difference between the terms "serious" and "severe", the following note of clarification is provided: "Severe" is often used to describe intensity of a specific event, which may be of relatively minor medical significance. "Seriousness" is the regulatory definition supplied above.

8.2 Causality Assessment

The Investigator and the Local Safety Monitor both determine causality. It is expected that communication and consultation may occur in the assessment of the causality of AEs. The greatest degree of causal relationship (definite > probable > possible > unlikely related > not related) determined by either the Investigator or Local Safety Monitor after their discussions (and review of the volunteer if necessary) will determine the ultimate classification of the AE. Definite (4), probable (3) and possible (2) are considered to be related. No relationship (0) and unlikely (1) are considered to be unrelated. The following guidelines will be used to assess the relationship of all AEs to the administration of the vaccine, with the premise that all local reactions will be considered causally related to the vaccination:

0 = No relationship:
- No temporal relationship to vaccine; *and*
- Alternate aetiology (clinical state, environmental or other interventions); *and*
- Does not follow known pattern of response to the vaccine

1 = Unlikely relationship:
- Unlikely temporal relationship to vaccine; *and*
- Alternate aetiology likely (clinical state, environmental or other interventions); *and*
- Does not follow known typical or plausible pattern of response to the vaccine

2 = Possible relationship:
- Reasonable temporal relationship to vaccine; *or*
- Event not readily produced by clinical state, environmental or other interventions; *or*
- Similar pattern of response to that seen with the vaccine

3 = Probable relationship:
- Reasonable temporal relationship to vaccine; *and*
- Event not readily produced by clinical state, environment, or other interventions *or*

- Known pattern of response seen with the vaccine

4 = Definite relationship:

- Reasonable temporal relationship to vaccine; *and*
- Event not readily produced by clinical state, environment, or other interventions; *and*
- Known pattern of response seen with the vaccine

8.3 Clinical Assessment

All AEs (whether reported by the subject or solicited by the Investigator) will be clinically assessed at each visit and recorded with start and stop dates and details of any treatment undergone. Final grading of adverse events will be determined by the Investigator on the trial.

This information will be **collected** as follows:

- Subjects are asked to complete diary cards for 14 days after MVA85A vaccination by either route. They are provided with a thermometer and tape measure to enable daily recording of temperature and local redness and swelling at the intradermal injection site. They are also provided with a spirometer to record pulmonary function.
- Outside the diary card periods, expected local and systemic AEs (listed in table 4 below) will be solicited, in other words specifically asked about at each visit, and graded by severity and causality (as detailed in sections 9.2 and 9.4).
- Subjects will be given the opportunity to report any other new symptoms since the last visit with start and stop dates and any treatments undergone.
- Vital signs will be performed in the normal manner at each visit.
- The overall appearance of the lung mucosa will be assessed at the day 7 bronchoscopy.

	**Adverse event**
**Local (intradermal route)**	Pain/tenderness at the injection site
	Redness at the injection site
	Swelling at injection site
	Warmth at the injection site
	Itch at the injection site
	Scaling/pustules at the injection site
**Local (aerosol inhaled route)**	Cough
	Sore throat
	Wheeze
	Shortness of breath
	Sputum
	Haemoptysis
	Chest pain
**Systemic**	Documented fever (oral temperature > 37.5° C)
	Symptoms of feverishness
	Malaise
	Arthralgia
	Headache
	Myalgia
	Nausea / vomiting

Table 4: Routinely solicited local and systemic adverse events

The information will be **recorded** as follows:

- During the diary card period, all solicited AEs (listed in table 4) and any additional unexpected AEs are captured in the diary card and at clinic visits (CRF)
- Outside the diary card periods all AEs will be documented in the AE line listing section of the CRF with start and stop dates and details of any treatments undergone.
- Vital signs will be recorded in the CRF.

8.4 Severity Assessment

AEs will be graded by severity as follows (where 0 = absent, 1 = mild, 2 = moderate, 3 = severe):

Adverse event	Grade	Measurement
Tenderness or pain at intradermal injection site	0	No pain at all
	1	Painful on touch; easily tolerated
	2	Painful when limb is moved; interferes with daily activity
	3	Severe pain at rest; prevents daily activity
Redness at intradermal injection site	0	0 mm
	1	1 – 50 mm
	2	51 – 100 mm
	3	> 100 mm
Swelling at intradermal injection site	0	0 mm
	1	1 – 20 mm
	2	21 – 50 mm
	3	> 50 mm
Fever (oral)	0	≤37.5°C
	1	37.6 – 38.0 °C
	2	38.1 – 39.0 °C
	3	>39.0 °C
Other AEs	0	Absence of symptom
	1	Awareness of symptom but tolerated; transient or mild discomfort; little or no medical intervention required
	2	Discomfort enough to cause limitation of usual activity; some medical intervention or therapy required
	3	Incapacitating, absent from work, or bed rest required; hospitalisation possible

Table 5. Severity grading for AEs

8.5 Laboratory Assessments

All laboratory results will be reviewed and recorded in the CRF after being signed by the Investigator. A note will be made of whether each result is normal, abnormal but not clinically significant, or abnormal *and* clinically significant. In the latter case trial eligibility will be reviewed and necessary referrals or tests will be offered.

The severity of laboratory test abnormalities will be assessed according to the scale in Table 6 (adapted from (67))

Laboratory Test	Grade 1	Grade 2	Grade 3
Hb (female) – decrease from testing laboratory LLN in gm/dl	>1.0 - <1.5	≥1.5 & <2.0	≥2.0
Hb (male) – decrease from testing laboratory LLN in gm/dl	≥1.5 & <2.0	≥2.0 & <2.5	≥2.5
Absolute neutrophil count (ANC, cells/mm^3)	1000-1499	500-999	<500
Leukopenia (WBC, cells/mm^3)	<3500 - ≥2500	<2500 - ≥1500	<1500
Platelets (cells/mm^3)	125,000 – 135,000	100,000 – 124,000	20,000-99,000
Bilirubin – when accompanied by any increase in Liver Function Test increase by factor	1.1 – 1.25 x ULN	1.26 – 1.5 x ULN	1.51 – 1.75 x ULN

ALT	1.25 – 2.5 x ULN	>2.6 – 5.0 x ULN	>5.0 x ULN
Creatinine	1.1 – 1.5 x ULN	>1.6 – 3.0 x ULN	>3.0 x ULN

Table 6: Severity grading criteria for clinically significant laboratory abnormalities (67)

8.6 Reporting Procedure for Adverse Events

Adverse events will be recorded in the CRF. Those adverse events likely to be related to the vaccine, whether serious or not, which persist at the end of the trial will be followed up by the Investigator until their complete disappearance. Moreover, any serious adverse event likely to be related to the vaccine and occurring after trial termination should be reported by the Investigator according to the procedure described below.

The outcome of any non-serious adverse event occurring within 30 days post-vaccination or any SAE reported during the entire trial will be assessed as:

- Recovered/resolved
- Not recovered/not resolved
- Recovering/resolving
- Recovered with sequelae/resolved with sequelae
- Fatal

Subjects who have moderate or severe on-going AEs at the completion of the trial will be advised to consult their General Practitioner (National Health Service) if the event is not considered to be related to the vaccine. A follow-up visit will be arranged to manage the problem and to determine the severity and duration of the event, if it is considered to be related to the vaccine. If appropriate, specialist review within the NHS will be arranged.

8. 7 Reporting of Serious Adverse Events

All SAEs will be documented accurately and notification deadlines respected as specified in the site-specific SOP. SAEs will be reported within 24 hours by the Investigator to the Sponsor or person with appropriate responsibility delegated from the Sponsor (Chief Investigator), and to the Local Safety Committee (see section 4.4). This will be performed by emailing an electronic version of the completed SAE Initial Report Form (CCVTM form VC004F1). Any relevant information concerning the SAE that becomes available after the SAE Initial Report Form has been sent (outcome, precise history, results of investigations, copy of hospital report, etc.) will be forwarded in a timely manner using the SAE Update Report Form (CCVTM form VC004F2). The Sponsor, or person with appropriate responsibility delegated from the Sponsor (Chief Investigator/medically qualified investigator), is responsible for commencing, maintaining and completing the SAE Sponsor Report Form (VC004F3). The anonymity of subjects shall be respected when forwarding this information.

SAEs which are not SUSARs will not normally be reported to the EC and MHRA, unless there is a clinically important increase in occurrence rate, an unexpected outcome, or a new event that is likely to affect safety of trial subjects.

In addition to these reporting requirements, the Investigator shall submit once a year throughout the trial, or on request, a safety report to the EC and MHRA (and a copy to the Sponsor).

We will follow every volunteer up for adverse event until their last visit. We will finish collection of AE data at the end of the trial, last volunteer last visit.

8.8 Reporting Procedures for SUSARs

The Chief Investigator will report all SUSARs to the MHRA and ethical committee(s) within required timelines. The Chief Investigator will also inform all investigators concerned of relevant information about SUSARs that could adversely affect the safety of participants

If the SAE is a SUSAR it will be reported to the EC and MHRA within 7 days of the Chief Investigator having first knowledge of the event for fatal and life-threatening cases and within 15 days for all other SUSARs (and the report copied to the Sponsor). Any deaths occurring during the trial will be reported to the Sponsor. For all deaths, available autopsy reports will be made available for reporting to the regulatory authorities.

8.9 Local Safety Committee

The local safety committee (LSC) will provide safety oversight for this trial. Its chair will be a clinician with extensive and relevant experience of infectious disease and clinical trials work. At the time of writing the chair is Dr Brian Angus, Clinical Tutor in Medicine, Honorary Consultant Physician and Director of the Centre for Tropical Medicine at Oxford University. There will be a minimum of two other appropriately qualified committee members as specified in the site-specific SOP for safety reporting.

8.10 Development Safety Update report

A development safety update report (DSUR) for each IMP is prepared annually, and submitted within 60 days of the anniversary of the first approval date from the regulatory authority for each IMP. This will be submitted by the chief Investigator to the competent authority and ethical committee(s).

8.11 Pregnancy

Subjects who become pregnant during the trial after the vaccination may continue trial procedures including venepuncture, if appropriate, at the discretion of the Investigator. Subjects who become pregnant before MVA85A vaccination will be withdrawn and will not be vaccinated. Subjects who become pregnant between vaccination and bronchoscopy will not undergo bronchoscopy. The Investigator will collect pregnancy information on any subject who becomes pregnant while participating in this trial. The subject will be followed up to determine the outcome of the pregnancy.

9. Statistics

9.1 Statistics

This is primarily a trial with descriptive endpoints. Twelve subjects will be recruited into each arm of the trial (i.e. 36 in total). Our previous experience with clinical trials using MVA85A over the last 10 years suggests that this sample size is a feasible number to recruit, screen, enroll, and follow up in practical terms, whilst also allowing the determination of any substantial and clinically significant differences in safety and cell-mediated immunity between the three groups. A twofold increase in the magnitude of the cellular immune response (as measured by Elispot response to 85A peptide stimulation) is considered immunologically meaningful. Extrapolating effect size and variability from the completed initial aerosol vaccine safety study (TB026), twelve volunteers are needed to have 80% power to detect an immunologically meaningful difference in the summed peptide pools and 8

volunteers to achieve the same for single peptide pool of 85A. This sample size is appropriate for a proof-of-concept Phase I safety study.

It is anticipated that the immunological endpoints will not follow a normal distribution. A log transformation will be applied and if the resulting data are normally distributed they will be summarised by group using means and standard deviations. The one-week and six-month responses will be compared between the three groups using a t-test. If the resulting data are not normally distributed then medians and interquartile ranges will be used to summarise the data and the Mann Whitney U test will be used for statistical comparisons. An area-under-curve analysis will be used to compare the overall responses over time between the three groups. This analysis will make use of data collected at all time points.

Differences between means or medians between the three groups will be presented along with their 95% confidence intervals. Within each group, paired data will be analysed using either a paired t-test or the Wilcoxon matched pairs test depending on the normality of the data.

10. Data Management

The Chief Investigator will be responsible for collecting, recording, analysing, and storing all the data accruing from the trial. These tasks may be delegated to other Investigators. Data will be stored in either paper CRFs held in a key-locked cabinet at the CCVTM, or electronic CRFs on the OpenClinica™ database (which is stored electronically on secure servers outsourced by OpenClinica™), or both. Some data may be duplicated anonymously into an electronic Microsoft Excel™ file on the CCVTM secure server.

10.1 Access to Data

Access to the source data documents will be provided if necessary to the EC and MHRA and to the Sponsor for trial-related monitoring and audit. All information relating to the trial and its subjects will be held in strict confidence, and in accordance with ICH E6 GCP and institutional requirements.

10.2 Data Recording and Record Keeping

Trial records will be held by the Chief Investigator for as long as required by legislation (currently until at least 2 years after the last marketing authorisation for the product or 2 years after discontinuation of clinical development of an investigational product), initially in a key-locked cabinet at the CCVTM and subsequently in a secure archive facility, and in accordance with the Data Protection Act. Subjects will be assigned individual unique trial numbers for identification on all trial records, except where the use of identifiable information is unavoidable (including on GP correspondence, registration documents, bank details request form and consent forms).

All clinical data will be recorded on paper case report forms and the volunteer files kept securely as described (key locked cabinets at the CCVTM during trial period and secure archive facility afterwards)

10.3 Source documents

Source documents are original documents, data and records from which the volunteer`s CRF data are obtained. For this study these will include the medical records (history and examination), the volunteer consent form, the TOPS database registration receipt, the blood and microbiology results, the chest xray report, the GP response letters, copy of the bronchoscopy report and any correspondence relating to that volunteer concerning medical/clinical issues.

10.4 Data Protection

The study protocol, documentation, bank details request form, data and all other information generated will be held in strict confidence and in accordance with the Data Protection Act. No information concerning the study or the data will be released to any unauthorized third party, without prior written approval of the sponsor.

11. Quality Assurance and Quality control

11.1. Quality Assurance

Investigator procedures

Approved site-specific SOPs will be used at all clinical and laboratory sites.

Modification to protocol

No substantial amendments to this protocol will be made without consultation with, and agreement of, the Sponsor. Any substantial amendments to the trial that appear necessary during the course of the trial must be discussed by the Investigator and Sponsor concurrently. If agreement is reached concerning the need for an amendment, it will be produced in writing by the Chief Investigator and will be made a formal part of the protocol following ethical and regulatory approval.

An administrative change to the protocol is one that modifies administrative and logistical aspects of a protocol but does not affect the subjects' safety, the objectives of the trial and its progress or scientific value of the project. An administrative change is a non-substantial amendment and does not require EC approval.

The Chief Investigator is responsible for ensuring that changes to an approved trial, during the period for which EC approval has already been given, are not initiated without EC review and approval except to eliminate apparent immediate hazards to the subject.

Protocol deviation

All deviations from the protocol will be documented in a protocol deviation form and filed in the trial master file.

11.2. Monitoring

Data will be evaluated for compliance with the protocol and accuracy in relation to source documents. The trial will be conducted in accordance with procedures identified in the protocol. Regular monitoring will be performed according to ICH GCP. According to applicable SOPs, the Monitors will

verify that the clinical trial is initiated, conducted and completed, and data are generated, documented and reported in compliance with the protocol, GCP and the applicable regulatory requirements.

12. Ethical and Regulatory Considerations

12.1. Good Clinical Practice

This trial will be conducted in accordance with the principles of the Declaration of Helsinki as agreed by the World Medical Association General Assembly (Washington 2002), ICH Good Clinical Practice (GCP), the requirements of the Medicines for Human Use (Clinical Trial) Regulations 2004, and local regulatory requirements.

12.2. Ethical Review

A copy of the protocol, proposed informed consent form, other written subject information and the proposed advertising material will be submitted to an independent EC for written approval. The Investigators will submit and, where necessary, obtain approval from the EC for all subsequent substantial amendments to the protocol and informed consent document.

12.3. Informed Consent

Written informed consent will be obtained at screening as detailed in section 7.2.

12.4. Serious Breaches

The Medicines for Human Use (Clinical Trials) Regulations contain a requirement for the notification of "serious breaches" to the MHRA within 7 days of the Chief Investigator becoming aware of the breach. A serious breach is defined as “A breach of GCP or the trial protocol which is likely to affect to a significant degree:

- the safety or physical or mental integrity of the subjects of the trial
- the scientific value of the trial”

In the event that a serious breach is suspected, the Sponsor will be contacted as soon as possible.

13. Finance/Compensation/Insurance

13.1. Finance

This trial will be financed by research grants from The Wellcome Trust and Biomedical Research Council held by Prof Helen McShane and a NIHR BRC clinical research training fellowship held by Dr Zita-Rose Manjaly Thomas.

13.2. Compensation

Subjects will be compensated *pro rata* for their time and travel and for trial procedures while participating in the trial, amounting to a total of approximately £800, depending on the exact number of visits and whether any repeat or additional visits are necessary.

13.3. Insurance

The University has a specialist insurance policy in place which would operate in the event of any participant suffering harm as a result of their involvement in the research (Newline Underwriting Management Ltd, at Lloyd’s of London, policy numbered :WD1200463).

14. Publication Policy

When the trial is complete, a manuscript describing the primary trial results will be written and published in a peer-reviewed, open access journal. International guidelines will be followed regarding authorship. There may also be secondary publications on more exploratory results.

15. REFERENCES

1. Dye C, Scheele S, Dolin P, Pathania V, Raviglione MC. Consensus statement. Global burden of tuberculosis: estimated incidence, prevalence, and mortality by country. WHO Global Surveillance and Monitoring Project. JAMA : the journal of the American Medical Association. 1999;282(7):677-86. Epub 1999/10/12.
2. Organization WH. Global tuberculosis report 2012. http://appswhoint/iris/bitstream/10665/75938/1/9789241564502_engpdf. 2012.
3. Corbett EL, Marston B, Churchyard GJ, De Cock KM. Tuberculosis in sub-Saharan Africa: opportunities, challenges, and change in the era of antiretroviral treatment. Lancet. 2006;367(9514):926-37. Epub 2006/03/21.
4. Colditz GA, Brewer TF, Berkey CS, Wilson ME, Burdick E, Fineberg HV, et al. Efficacy of BCG vaccine in the prevention of tuberculosis. Meta-analysis of the published literature. JAMA : the journal of the American Medical Association. 1994;271(9):698-702.
5. Fine PE. The BCG story: lessons from the past and implications for the future. Reviews of infectious diseases. 1989;11 Suppl 2:S353-9. Epub 1989/03/01.
6. Trunz BB, Fine P, Dye C. Effect of BCG vaccination on childhood tuberculous meningitis and miliary tuberculosis worldwide: a meta-analysis and assessment of cost-effectiveness. Lancet. 2006;367(9517):1173-80. Epub 2006/04/18.
7. Rodrigues LC, Diwan VK, Wheeler JG. Protective effect of BCG against tuberculous meningitis and miliary tuberculosis: a meta-analysis. Int J Epidemiol. 1993;22(6):1154-8. Epub 1993/12/01.
8. McShane H, Pathan AA, Sander CR, Keating SM, Gilbert SC, Huygen K, et al. Recombinant modified vaccinia virus Ankara expressing antigen 85A boosts BCG-primed and naturally acquired antimycobacterial immunity in humans. Nature medicine. 2004;10(11):1240-4. Epub 2004/10/27.
9. Tameris MD, Hatherill M, Landry BS, Scriba TJ, Snowden MA, Lockhart S, et al. Safety and efficacy of MVA85A, a new tuberculosis vaccine, in infants previously vaccinated with BCG: a randomised, placebo-controlled phase 2b trial. Lancet. 2013. Epub 2013/02/09.
10. Goonetilleke NP, McShane H, Hannan CM, Anderson RJ, Brookes RH, Hill AV. Enhanced immunogenicity and protective efficacy against Mycobacterium tuberculosis of bacille Calmette-Guerin vaccine using mucosal administration and boosting with a recombinant modified vaccinia virus Ankara. Journal of immunology. 2003;171(3):1602-9. Epub 2003/07/23.
11. Santosuosso M, McCormick S, Zhang X, Zganiacz A, Xing Z. Intranasal boosting with an adenovirus-vectored vaccine markedly enhances protection by parenteral Mycobacterium bovis BCG immunization against pulmonary tuberculosis. Infect Immun. 2006;74(8):4634-43. Epub 2006/07/25.
12. Xing Z, McFarland CT, Sallenave JM, Izzo A, Wang J, McMurray DN. Intranasal mucosal boosting with an adenovirus-vectored vaccine markedly enhances the protection of BCG-primed guinea pigs against pulmonary tuberculosis. PloS one. 2009;4(6):e5856. Epub 2009/06/12.
13. Xing Z, Lichty BD. Use of recombinant virus-vectored tuberculosis vaccines for respiratory mucosal immunization. Tuberculosis. 2006;86(3-4):211-7. Epub 2006/03/01.
14. Sabin AB. Immunization against measles by aerosol. Reviews of infectious diseases. 1983;5(3):514-23. Epub 1983/05/01.
15. Sabin AB, Flores Arechiga A, Fernandez de Castro J, Sever JL, Madden DL, Shekarchi I, et al. Successful immunization of children with and without maternal antibody by aerosolized measles vaccine. I. Different results with undiluted human diploid cell and chick embryo fibroblast vaccines. JAMA : the journal of the American Medical Association. 1983;249(19):2651-62. Epub 1983/05/20.
16. Dilraj A, Cutts FT, de Castro JF, Wheeler JG, Brown D, Roth C, et al. Response to different measles vaccine strains given by aerosol and subcutaneous routes to schoolchildren: a randomised trial. Lancet. 2000;355(9206):798-803. Epub 2000/03/11.
17. Dilraj A, Sukhoo R, Cutts FT, Bennett JV. Aerosol and subcutaneous measles vaccine: measles antibody responses 6 years after re-vaccination. Vaccine. 2007;25(21):4170-4. Epub 2007/04/06.
18. LiCalsi C, Maniaci MJ, Christensen T, Phillips E, Ward GH, Witham C. A powder formulation of measles vaccine for aerosol delivery. Vaccine. 2001;19(17-19):2629-36. Epub 2001/03/21.
19. Bellanti JA, Zeligs BJ, Mendez-Inocencio J, Garcia-Garcia ML, Islas-Romero R, Omidvar B, et al. Immunologic studies of specific mucosal and systemic immune responses in Mexican school

children after booster aerosol or subcutaneous immunization with measles vaccine. Vaccine. 2004;22(9-10):1214-20. Epub 2004/03/09.
20. Hiremath GS, Omer SB. A meta-analysis of studies comparing the respiratory route with the subcutaneous route of measles vaccine administration. Human vaccines. 2005;1(1):30-6. Epub 2006/10/14.
21. Cutts FT, Clements CJ, Bennett JV. Alternative routes of measles immunization: a review. Biologicals : journal of the International Association of Biological Standardization. 1997;25(3):323-38. Epub 1997/11/05.
22. Belshe RB, Mendelman PM, Treanor J, King J, Gruber WC, Piedra P, et al. The efficacy of live attenuated, cold-adapted, trivalent, intranasal influenzavirus vaccine in children. The New England journal of medicine. 1998;338(20):1405-12. Epub 1998/05/15.
23. Belshe RB, Edwards KM, Vesikari T, Black SV, Walker RE, Hultquist M, et al. Live attenuated versus inactivated influenza vaccine in infants and young children. The New England journal of medicine. 2007;356(7):685-96. Epub 2007/02/16.
24. Gaglani MJ, Piedra PA, Herschler GB, Griffith ME, Kozinetz CA, Riggs MW, et al. Direct and total effectiveness of the intranasal, live-attenuated, trivalent cold-adapted influenza virus vaccine against the 2000-2001 influenza A(H1N1) and B epidemic in healthy children. Archives of pediatrics & adolescent medicine. 2004;158(1):65-73. Epub 2004/01/07.
25. Langley JM, Halperin SA, McNeil S, Smith B, Jones T, Burt D, et al. Safety and immunogenicity of a Proteosome -trivalent inactivated influenza vaccine, given nasally to healthy adults. Vaccine. 2006;24(10):1601-8. Epub 2005/11/24.
26. Stephenson I, Zambon MC, Rudin A, Colegate A, Podda A, Bugarini R, et al. Phase I evaluation of intranasal trivalent inactivated influenza vaccine with nontoxigenic Escherichia coli enterotoxin and novel biovector as mucosal adjuvants, using adult volunteers. Journal of virology. 2006;80(10):4962-70. Epub 2006/04/28.
27. Omer SB, Hiremath GS, Halsey NA. Respiratory administration of measles vaccine. Lancet.375(9716):706-8. Epub 2010/03/02.
28. Song K, Bolton DL, Wei CJ, Wilson RL, Camp JV, Bao S, et al. Genetic immunization in the lung induces potent local and systemic immune responses. Proceedings of the National Academy of Sciences of the United States of America. 2010;107(51):22213-8. Epub 2010/12/08.
29. Tucker SN, Tingley DW, Scallan CD. Oral adenoviral-based vaccines: historical perspective and future opportunity. Expert review of vaccines. 2008;7(1):25-31. Epub 2008/02/07.
30. Belyakov IM, Moss B, Strober W, Berzofsky JA. Mucosal vaccination overcomes the barrier to recombinant vaccinia immunization caused by preexisting poxvirus immunity. Proceedings of the National Academy of Sciences of the United States of America. 1999;96(8):4512-7. Epub 1999/04/14.
31. Appaiahgari MB, Pandey RM, Vrati S. Seroprevalence of neutralizing antibodies to adenovirus type 5 among children in India: implications for recombinant adenovirus-based vaccines. Clinical and vaccine immunology : CVI. 2007;14(8):1053-5. Epub 2007/06/29.
32. Thorner AR, Vogels R, Kaspers J, Weverling GJ, Holterman L, Lemckert AA, et al. Age dependence of adenovirus-specific neutralizing antibody titers in individuals from sub-Saharan Africa. Journal of clinical microbiology. 2006;44(10):3781-3. Epub 2006/10/06.
33. Ersching J, Hernandez MI, Cezarotto FS, Ferreira JD, Martins AB, Switzer WM, et al. Neutralizing antibodies to human and simian adenoviruses in humans and New-World monkeys. Virology. 2010;407(1):1-6. Epub 2010/08/28.
34. Dudareva M, Andrews L, Gilbert SC, Bejon P, Marsh K, Mwacharo J, et al. Prevalence of serum neutralizing antibodies against chimpanzee adenovirus 63 and human adenovirus 5 in Kenyan children, in the context of vaccine vector efficacy. Vaccine. 2009;27(27):3501-4. Epub 2009/05/26.
35. White AD, Sibley L, Dennis MJ, Gooch K, Betts G, Edwards N, et al. Evaluation of the Safety and Immunogenicity of a Candidate Tuberculosis Vaccine, MVA85A, Delivered by Aerosol to the Lungs of Macaques. Clinical and vaccine immunology : CVI. 2013;20(5):663-72. Epub 2013/03/01.
36. Xiang ZQ, Gao GP, Reyes-Sandoval A, Li Y, Wilson JM, Ertl HC. Oral vaccination of mice with adenoviral vectors is not impaired by preexisting immunity to the vaccine carrier. Journal of virology. 2003;77(20):10780-9. Epub 2003/09/27.
37. Hodges BL, Taylor KM, Chu Q, Scull SE, Serriello RG, Anderson SC, et al. Local delivery of a viral vector mitigates neutralization by antiviral antibodies and results in efficient transduction of rabbit liver. Molecular therapy : the journal of the American Society of Gene Therapy. 2005;12(6):1043-51. Epub 2005/09/06.
38. Stickl H, Hochstein-Mintzel V, Mayr A, Huber HC, Schafer H, Holzner A. [MVA vaccination against smallpox: clinical tests with an attenuated live vaccinia virus strain (MVA) (author's transl)].

Dtsch Med Wochenschr. 1974;99(47):2386-92. Epub 1974/11/22. MVA-Stufenimpfung gegen Pocken. Klinische Erprobung des attenuierten Pocken-Lebendimpfstoffes, Stamm MVA.
39. Mayr A, Stickl H, Muller HK, Danner K, Singer H. [The smallpox vaccination strain MVA: marker, genetic structure, experience gained with the parenteral vaccination and behavior in organisms with a debilitated defence mechanism (author's transl)]. Zentralbl Bakteriol B. 1978;167(5-6):375-90. Epub 1978/12/01. Der Pockenimpfstamm MVA: Marker, genetische Struktur, Erfahrungen mit der parenteralen Schutzimpfung und Verhalten im abwehrgeschwachten Organismus.
40. Hochstein-Mintzel V, Huber HC, Stickl H. [Virulence and immunogenicity of a modified vaccinia virus (strain MVA) (author's transl)]. Z Immunitatsforsch Exp Klin Immunol. 1972;144(2):104-56. Epub 1972/09/01. Virulenz und Immunogenitat eines modifizierten Vaccinia-Virus (Stamm MVA).
41. Hochstein-Mintzel V, Hanichen T, Huber HC, Stickl H. [An attenuated strain of vaccinia virus (MVA). Successful intramuscular immunization against vaccinia and variola (author's transl)]. Zentralbl Bakteriol Orig A. 1975;230(3):283-97. Epub 1975/01/01. Vaccinia- und variolaprotektive Wirkung des modifizierten Vaccinia-Stammes MVA bei intramuskularer Immunisierung.
42. Goonetilleke N, Moore S, Dally L, Winstone N, Cebere I, Mahmoud A, et al. Induction of multifunctional human immunodeficiency virus type 1 (HIV-1)-specific T cells capable of proliferation in healthy subjects by using a prime-boost regimen of DNA- and modified vaccinia virus Ankara-vectored vaccines expressing HIV-1 Gag coupled to CD8+ T-cell epitopes. J Virol. 2006;80(10):4717-28. Epub 2006/04/28.
43. Bejon P, Peshu N, Gilbert SC, Lowe BS, Molyneux CS, Forsdyke J, et al. Safety profile of the viral vectors of attenuated fowlpox strain FP9 and modified vaccinia virus Ankara recombinant for either of 2 preerythrocytic malaria antigens, ME-TRAP or the circumsporozoite protein, in children and adults in Kenya. Clin Infect Dis. 2006;42(8):1102-10. Epub 2006/04/01.
44. Gherardi MM, Esteban M. Recombinant poxviruses as mucosal vaccine vectors. J Gen Virol. 2005;86(Pt 11):2925-36. Epub 2005/10/18.
45. Belisle JT, Vissa VD, Sievert T, Takayama K, Brennan PJ, Besra GS. Role of the major antigen of Mycobacterium tuberculosis in cell wall biogenesis. Science. 1997;276(5317):1420-2. Epub 1997/05/30.
46. Denis O, Tanghe A, Palfliet K, Jurion F, van den Berg TP, Vanonckelen A, et al. Vaccination with plasmid DNA encoding mycobacterial antigen 85A stimulates a CD4+ and CD8+ T-cell epitopic repertoire broader than that stimulated by Mycobacterium tuberculosis H37Rv infection. Infect Immun. 1998;66(4):1527-33. Epub 1998/04/07.
47. McShane H, Brookes R, Gilbert SC, Hill AV. Enhanced immunogenicity of CD4(+) t-cell responses and protective efficacy of a DNA-modified vaccinia virus Ankara prime-boost vaccination regimen for murine tuberculosis. Infect Immun. 2001;69(2):681-6. Epub 2001/02/13.
48. Vordermeier HM, Rhodes SG, Dean G, Goonetilleke N, Huygen K, Hill AV, et al. Cellular immune responses induced in cattle by heterologous prime-boost vaccination using recombinant viruses and bacille Calmette-Guerin. Immunology. 2004;112(3):461-70. Epub 2004/06/16.
49. Huygen K, Content J, Denis O, Montgomery DL, Yawman AM, Deck RR, et al. Immunogenicity and protective efficacy of a tuberculosis DNA vaccine. Nat Med. 1996;2(8):893-8. Epub 1996/08/01.
50. Verreck FA, Vervenne RA, Kondova I, van Kralingen KW, Remarque EJ, Braskamp G, et al. MVA.85A boosting of BCG and an attenuated, phoP deficient M. tuberculosis vaccine both show protective efficacy against tuberculosis in rhesus macaques. PLoS One. 2009;4(4):e5264. Epub 2009/04/16.
51. Williams A, Hatch GJ, Clark SO, Gooch KE, Hatch KA, Hall GA, et al. Evaluation of vaccines in the EU TB Vaccine Cluster using a guinea pig aerosol infection model of tuberculosis. Tuberculosis (Edinb). 2005;85(1-2):29-38. Epub 2005/02/03.
52. Vordermeier HM, Dean GS, Rosenkrands I, Agger EM, Andersen P, Kaveh DA, et al. Adjuvants induce distinct immunological phenotypes in a cattle tuberculosis vaccine model. Clin Vaccine Immunol. 2009. Epub 2009/07/31.
53. Pathan AA, Minassian AM, Sander CR, Rowland R, Porter DW, Poulton ID, et al. Effect of vaccine dose on the safety and immunogenicity of a candidate TB vaccine, MVA85A, in BCG vaccinated UK adults. Vaccine. 2012;30(38):5616-24. Epub 2012/07/14.
54. Scriba TJ, Tameris M, Mansoor N, Smit E, van der Merwe L, Mauff K, et al. Dose-finding study of the novel tuberculosis vaccine, MVA85A, in healthy BCG-vaccinated infants. The Journal of infectious diseases. 2011;203(12):1832-43. Epub 2011/05/25.
55. McShane H. Developing an improved vaccine against tuberculosis. Expert Rev Vaccines. 2004;3(3):299-306. Epub 2004/06/05.

56. WHO. IVR strategic plan 2010 - 2020. http://www.who.int/immunization/documents/WHO_IVB_10.02/en/index.html (accessed 28 July 2010) 2010.
57. Santosuosso M, McCormick S, Roediger E, Zhang X, Zganiacz A, Lichty BD, et al. Mucosal luminal manipulation of T cell geography switches on protective efficacy by otherwise ineffective parenteral genetic immunization. J Immunol. 2007;178(4):2387-95. Epub 2007/02/06.
58. Kariyawasam HH, Aizen M, Kay AB, Robinson DS. Safety and tolerability of three consecutive bronchoscopies after allergen challenge in volunteers with mild asthma. Thorax. 2007;62(6):557-8. Epub 2007/05/31.
59. Elston WJ, Whittaker AJ, Khan LN, Flood-Page P, Ramsay C, Jeffery PK, et al. Safety of research bronchoscopy, biopsy and bronchoalveolar lavage in asthma. The European respiratory journal : official journal of the European Society for Clinical Respiratory Physiology. 2004;24(3):375-7. Epub 2004/09/11.
60. Busse WW, Wanner A, Adams K, Reynolds HY, Castro M, Chowdhury B, et al. Investigative bronchoprovocation and bronchoscopy in airway diseases. Am J Respir Crit Care Med. 2005;172(7):807-16. Epub 2005/07/16.
61. Ho LP, Davis M, Denison A, Wood FT, Greening AP. Reduced interleukin-18 levels in BAL specimens from patients with asthma compared to patients with sarcoidosis and healthy control subjects. Chest. 2002;121(5):1421-6. Epub 2002/05/15.
62. Flynn JL, Chan J. Immunology of tuberculosis. Annual review of immunology. 2001;19:93-129. Epub 2001/03/13.
63. Kongerud J, Madden MC, Hazucha M, Peden D. Nasal responses in asthmatic and nonasthmatic subjects following exposure to diesel exhaust particles. Inhalation toxicology. 2006;18(9):589-94.
64. Corbett M, Bogers WM, Heeney JL, Gerber S, Genin C, Didierlaurent A, et al. Aerosol immunization with NYVAC and MVA vectored vaccines is safe, simple, and immunogenic. Proc Natl Acad Sci U S A. 2008;105(6):2046-51. Epub 2008/02/14.
65. Wong-Chew RM, Islas-Romero R, Garcia-Garcia Mde L, Beeler JA, Audet S, Santos-Preciado JI, et al. Immunogenicity of aerosol measles vaccine given as the primary measles immunization to nine-month-old Mexican children. Vaccine. 2006;24(5):683-90. Epub 2005/09/13.
66. OMRON. OMRON Microair U22 website http://www.omron-healthcare.com/en/product/respiratory_therapy/NE-U22-E.html (accessed 08 October 2010). 2010.
67. FDA. Toxicity Grading Scale for Healthy Adult & Adolescent Volunteers Enrolled in Preventative Vaccine
Clinical Trials. 2007.
