# Supplementary material for: Alternate aerosol and systemic immunisation with a recombinant viral vector for tuberculosis, MVA85A: A phase I randomised controlled trial
Source: PLoS Med. 2019 Apr 30;16(4):e1002790. doi: 10.1371/journal.pmed.1002790 (PMC6490884; doi:10.1371/journal.pmed.1002790)
Supplement: S1 Table — (PDF) [file pmed.1002790.s006.pdf]

S1 Table. Baseline characteristics

Characteristic	Group 1<br>Aerosol; ID<br>(n = 12)	Group 2<br>ID; Aerosol<br>(n = 13)	Group 3<br>ID; ID<br>(n = 12)	P value
Female, n (%)	11 (92%)	11 (85%)	6 (50%)	0.04*
Median age in years (range)	26 (22-42)	26 (22-39)	27 (21-41)	0.71
Median time interval since BCG in years (range)	13 (1-36)	17 (1-28)	14 (1-30)	0.99
BMI (kg/m^2) (range)	23.8 (18.2-37.5)	24.0 (18.3-29.6)	22.6 (19.1-33.7)	0.86
Spirometry				
Median % predicted FEV_1 (range)	102.5 (90-117)	91 .5 (82-103)	98 (82-120)	0.66
Median % predicted FVC (range)	101 (95-108)	97 (87-105)	96.5 (85-114)	0.25
Median baseline % S_pO_2 (range)	97 (95-99)	98 (96-99)	98 (96-99)	0.25
Continent of birth				
Europe	10	12	12	
Africa	0	1	0	
Americas	1	0	0	
Asia	1	0	0	

ID: Intradermal vaccination route; BCG: Bacillus Calmette-Guérin; BMI: Body mass index; FEV_1: forced expiratory volume in 1 second; FVC: forced vital capacity; SaO_2: peripheral oxygen saturation

* Group 3 had significantly more male participants than Group 1 and Group 2
