# Supplementary material for: Alternate aerosol and systemic immunisation with a recombinant viral vector for tuberculosis, MVA85A: A phase I randomised controlled trial
Source: PLoS Med. 2019 Apr 30;16(4):e1002790. doi: 10.1371/journal.pmed.1002790 (PMC6490884; doi:10.1371/journal.pmed.1002790)
Supplement: S2 Table — (PDF) [file pmed.1002790.s007.pdf]

S2 Table. The numbers of subjects within each group reporting each related adverse event[1]

Adverse events	Route	Aerosol Day 0	Intradermal Day 28	Intradermal Day 0	Aerosol Day 28	Intradermal Day 0	Intradermal Day 28
	Group	1	1	2	2	3	3
	N	12	12	13[2]	9[3]	12	12
**Solicited respiratory AEs**							
Chest pain		1 (8%)	0	0	5 (56%)	0	0
Haemoptysis		0	0	0	0	0	0
Coughing phlegm		0	0	2 (15%)	3 (33%)	3 (25%)	0
Shortness of breath		2 (17%)	0	1 (8%)	4 (44%)	1 (8%)	0
Wheeze		0	0	0	2 (22%)	2 (17%)	0
Sore throat		2 (17%)	2 (17%)	3 (23%)	2 (22%)	3 (25%)	5 (42%)
Cough		4 (33%)	2 (17%)	4 (31%)	7 (78%)	4 (33%)	5 (42%)
**Unsolicited respiratory AEs[4]**							
Nasal congestion		1 (8%)	0	0	0	0	0
Rhinorrhoea		0	0	1(8%)	0	1 (8%)	1 (8%)
Face feeling swollen		0	0	0	1 (11%)	0	0
Chest discomfort		0	0	0	1 (11%)	0	0

**Solicited systemic AEs**						
Malaise	3 (25%)	2 (17%)	4 (31%)	7 (78%)	1 (8%)	2 (17%)
Nausea	1 (8%)	1 (8%)	0	5 (56%)	1 (8%)	1 (8%)
Fatigue	5 (42%)	6 (50%)	3 (23%)	7 (78%)	5 (42%)	4 (33%)
Headache	5 (42%)	5 (42%)	6 (46%)	8 (89%)	4 (33%)	4 (33%)
Arthralgia	3 (25%)	2 (17%)	2 (15%)	4 (44%)	4 (33%)	1 (8%)
Myalgia	5 (42%)	6 (50%)	5 (38%)	7 (78%)	6 (50%)	4 (33%)
Felt feverish	2 (17%)	2 (17%)	3 (23%)	7 (78%)	3 (25%)	3 (25%)
Documented fever	2 (17%)	0	1 (8%)	6 (67%)	1 (8%)	0
**Unsolicited systemic AEs[4]**						
Neck or Back pain	0	1 (8%)	0	1 (11%)	0	0
Chills	1 (8%)	0	0	0	0	0
Presyncope	0	0	2 (15%)	2 (22%)	0	0
Syncope	0	1(8%)	0	0	0	0
Insomnia	0	0	0	0	1 (8%)	0
Muscle spasm	0	0	0	0	0	1 (8%)

**Solicited local AEs**						
Local erythema	-	12 (100%)	13 (100%)	-	12 (100%)	12 (100%)
Local swelling	-	12 (100%)	13 (100%)	-	12 (100%)	12 (100%)
Local scaling	-	12 (100%)	13 (100%)	-	12 (100%)	8 (67%)
Local pain	-	11 (92%)	12 (92%)	-	11 (92%)	4 (100%)
Local pruritus	-	10 (83%)	12 (92%)	-	11 (92%)	9 (75%)
Local warmth	-	11 (92%)	12 (92%)	-	9 (75%)	8 (67%)
**Unsolicited local AEs[4]**						
Axillary pain	-	0	2 (15%)	-	2 (17%)	1 (8%)
Lymphadenopathy	-	0	2 (15%)	-	0	1 (8%)
**Laboratory AEs[5]**						
Anaemia	0	0	1 (11%)	0	0	0
Lymphopaenia	0	0	0	1 (11%)	0	0
Neutropaenia	0	0	0	1 (11%)	0	0

[1] All related adverse event data. Respiratory and solicited adverse event data shown for the first 7 days following each vaccination, as AEs following bronchoscopy at Day 7 were not deemed to be related. All adverse event data at the injection site and laboratory parameters data for 1 month period following each vaccination were deemed to be related.
[2] 3 placebo controls excluded from analysis
[3] Includes one subject who withdrew post first vaccination but prior to boost vaccination so was replaced
[4] Unsolicited symptoms are those deemed possibly, probably or definitely related to the vaccine
[5] All laboratory AEs were a maximum of Grade 1 severity
