# Supplementary material for: Alternate aerosol and systemic immunisation with a recombinant viral vector for tuberculosis, MVA85A: A phase I randomised controlled trial
Source: PLoS Med. 2019 Apr 30;16(4):e1002790. doi: 10.1371/journal.pmed.1002790 (PMC6490884; doi:10.1371/journal.pmed.1002790)
Supplement: S3 Table — (PDF) [file pmed.1002790.s008.pdf]

S3 Table. Systemic adverse events (AEs) by group, by vaccination and by severity

Number of participants per group		Group 1<br>12	Group 2<br>13*	Group 3<br>12	Total AEs
**Systemic AEs by group**					
**Total possible systemic AEs[#]**		**192**	**176**	**192**	**560**
**Severity**	Mild	35	43	42	120
	Moderate	10	17	2	29
	Severe	5	15	0	20
**Total**	**n (frequency)**	**50 (0.26)**	**75 (0.43)**	**44 (0.23)**	**169 (0.30)**

Systemic AEs by route		Group 1<br>Day 0<br>Aerosol	Group 1<br>Day 28<br>Intradermal	Group 2<br>Day 0<br>Intradermal	Group 2<br>Day 28<br>Aerosol	Group 3<br>Day 0<br>Intradermal	Group 3<br>Day 28<br>Intradermal	Total
**Total possible systemic AEs**		**96**	**96**	**104**	**72**	**96**	**96**	
	Mild	18	17	22	21	24	18	
	Moderate	5	5	2	15	1	1	
	Severe	3	2	0	15	0	0	
**Total**	**n (frequency)**	**26 (0.27)**	**24 (0.25)**	**24 (0.23)**	**51 (0.71)**	**25 (0.26)**	**19 (0.20)**	**169 (0.30)**

*Includes one subject who withdrew post first vaccination but prior to boost vaccination so was replaced. The last three enrolled subjects received placebo not MVA85A boost following safety concerns

[#] Maximum of 8 solicited systemic adverse events per subject. Total possible adverse events calculated as follows. Group 1: 12 subjects x 8 solicited adverse events x 2 vaccinations= 192; Group 2: (13 subjects x 8 solicited adverse events received 1st vaccination) + (9 subjects x 8 solicited adverse events received 2nd vaccination) = 176; Group 3: 12 subjects x 8 solicited adverse events x 2 vaccinations= 192
