# Supplementary material for: Alternate aerosol and systemic immunisation with a recombinant viral vector for tuberculosis, MVA85A: A phase I randomised controlled trial
Source: PLoS Med. 2019 Apr 30;16(4):e1002790. doi: 10.1371/journal.pmed.1002790 (PMC6490884; doi:10.1371/journal.pmed.1002790)
Supplement: S4 Table — (PDF) [file pmed.1002790.s009.pdf]

S4 Table. Respiratory adverse events (AEs) by group, by vaccination and by severity

Number of participants per group		Group 1<br>12		Group 2<br>13*		Group 3<br>12		Total AEs
**Respiratory AEs by group**								
**Total possible respiratory AEs**[+]		**168**		**154**		**168**		**490**
**Severity**	Mild	13		27		23		63
	Moderate	0		5		0		5
	Severe	0		1		0		1
**Total**	**n (frequency)**	**13 (0.08)**		**33 (0.21)**		**23 (0.14)**		**69 (0.14)**
**Respiratory AEs by route**		**Group 1<br>Day 0<br>Aerosol**	**Group 1<br>Day 28<br>Intradermal**	**Group 2<br>Day 0<br>Intradermal**	**Group 2<br>Day 28<br>Aerosol**	**Group 3<br>Day 0<br>Intradermal**	**Group 3<br>Day 28<br>Intradermal**	**Total**
**Total possible respiratory AEs**		**84**	**84**	**91**	**63**	**84**	**84**	
	Mild	9	4	10	17	13	10	
	Moderate	0	0	0	5	0	0	
	Severe	0	0	0	1	0	0	
**Total**	**n (frequency)**	**9 (0.11)**	**4 (0.05)**	**10 (0.11)**	**23 (0.37)**	**13 (0.15)**	**10 (0.12)**	**69 (0.14)**

*Includes one subject who withdrew post first vaccination but prior to boost vaccination so was replaced. The last three enrolled subjects received placebo not MVA85A boost following safety concerns

[+] Maximum of 7 solicited respiratory adverse events per subject. Total possible adverse events calculated as follows. Group 1: 12 subjects x 7 respiratory adverse events x 2 vaccinations= 168; Group 2: (13 subjects x 7 solicited adverse events received 1st vaccination) + (9 subjects x 7 solicited adverse events received 2nd vaccination) = 154; Group 3: 12 subjects x 7 solicited adverse events x 2 vaccinations= 168
