# Supplementary material for: Alternate aerosol and systemic immunisation with a recombinant viral vector for tuberculosis, MVA85A: A phase I randomised controlled trial
Source: PLoS Med. 2019 Apr 30;16(4):e1002790. doi: 10.1371/journal.pmed.1002790 (PMC6490884; doi:10.1371/journal.pmed.1002790)
Supplement: S5 Table — (PDF) [file pmed.1002790.s010.pdf]

S5 Table. Solicited adverse events by volunteer, after each vaccination

Adverse events	Route	Aerosol Day 0	Intradermal Day 28	Intradermal Day 0	Aerosol Day 28	Intradermal Day 0	Intradermal Day 28
	**Group**	**1**	**1**	**2**	**2**	**3**	**3**
	**N**	**12**	**12**	**13+**	**9***	**12**	**12**
**Solicited respiratory AEs**							
Number of volunteers reporting at least one AE		5 (42%)	3 (25%)	5 (38%)	8 (89%)	7 (58%)	6 (50%)
Median AEs per volunteer (Range)		2 (1-3)	1 (1-2)	2 (1-3)	2 (1-5)	1 (1-5)	2 (1-2)
**Solicited systemic AEs**							
Number of volunteers reporting at least one AE		9 (70%)	9 (75%)	8 (62%)	8 (89%)	7 (58%)	6 (50%)
Median AEs per volunteer (Range)		2 (1-7)	2 (1-6)	3 (2-5)	7 (2-8)	3 (2-6)	3 (1-7)
**Solicited local AEs**							
Number of volunteers reporting at least one AE		-	12 (100%)	13 (100%)	-	12 (100%)	12 (100%)
Median AEs per volunteer (Range)		-	6 (4-6)	6 (4-6)	-	6 (5-6)	4.50 (2-6)

Median adverse events per volunteer calculated for volunteers who reported at least adverse event. Total possible solicited respiratory AEs per volunteer: 7; systemic AEs 8; Local AEs 6.

+Includes one subject who withdrew post first vaccination but prior to boost vaccination so was replaced

*3 placebo controls excluded from analysis
