# Supplementary material for: Alternate aerosol and systemic immunisation with a recombinant viral vector for tuberculosis, MVA85A: A phase I randomised controlled trial
Source: PLoS Med. 2019 Apr 30;16(4):e1002790. doi: 10.1371/journal.pmed.1002790 (PMC6490884; doi:10.1371/journal.pmed.1002790)
Supplement: S6 Table — (PDF) [file pmed.1002790.s011.pdf]

S6 Table. Solicited adverse events by volunteer, by group

Adverse events by individual	Group	1	2	3	P value
	N	12	9*	12	
**Solicited respiratory AEs**					
Median AEs per volunteer (Range)		0.5 (0-4)	2 (0-7)	2 (0-5)	0.12 (not significant)
**Solicited systemic AEs**					
Median AEs per volunteer (Range)		4 (0-10)	8 (0-13)	2 (0-12)	0.07 (not significant)
**Solicited local AEs**					
Median AEs per volunteer (Range)		7 (4-8)	7 (6-10)	10 (7-12)	Grp 1 vs Grp 3: p= 0.0001<br>Grp 2 vs Grp 3: p= 0.014

*3 placebo controls excluded from analysis
