# Supplementary material for: Alternate aerosol and systemic immunisation with a recombinant viral vector for tuberculosis, MVA85A: A phase I randomised controlled trial
Source: PLoS Med. 2019 Apr 30;16(4):e1002790. doi: 10.1371/journal.pmed.1002790 (PMC6490884; doi:10.1371/journal.pmed.1002790)
Supplement: S7 Table — (PDF) [file pmed.1002790.s012.pdf]

S7 Table. ELISpot responses statistical analysis: AUC.

**Ag85A**		
**Groups**	**P-value**	**95% CI (**SFC per 1 × 10^6 PBMCs)
1 vs 2	0.808	-30996 to 44597
2 vs 3	0.917	-35469 to 35518
1 vs 3	0.713	-23551 to 39739
**MVA-CD4**		
**Groups**	**P-value**	**95% CI**
1 vs 2	0.754	-10283 to 6377
2 vs 3	0.382	-18757 to 1922
1 vs 3	0.114	-389 to 10510
**MVA-CD8**		
**Groups**	**P-value**	**95% CI**
1 vs 2	0.702	-24041 to 45602
2 vs 3	0.464	-9506 to 39652
1 vs 3	0.291	-10886 to 50309
