# Supplementary material for: Alternate aerosol and systemic immunisation with a recombinant viral vector for tuberculosis, MVA85A: A phase I randomised controlled trial
Source: PLoS Med. 2019 Apr 30;16(4):e1002790. doi: 10.1371/journal.pmed.1002790 (PMC6490884; doi:10.1371/journal.pmed.1002790)
Supplement: S8 Table — One-way ANOVA (Kruskal–Wallis) adjusted for multiple comparisons. (PDF) [file pmed.1002790.s013.pdf]

S8 Table. Whole blood intracellular cytokines statistical analysis: Area Under the Curve (AUC). One-way-Anova (Kruskal_Wallis) adjusted for multiple comparisons

	P-value
**85A**	
**CD4 IFN-g**	0.8882
**CD8 IFN-g**	0.2313
**CD4 TNF-a**	0.9029
**CD8 TNF-a**	0.1814
**CD4 IL-2**	0.3911
**CD4 IL-17**	0.543
**MVA**	
**CD4 IFN-g**	0.3395
**CD8 INF-g**	0.0755
**CD4 TNF-a**	0.7261
**CD8 TNF-a**	0.5401
