# Supplementary material for: Alternate aerosol and systemic immunisation with a recombinant viral vector for tuberculosis, MVA85A: A phase I randomised controlled trial
Source: PLoS Med. 2019 Apr 30;16(4):e1002790. doi: 10.1371/journal.pmed.1002790 (PMC6490884; doi:10.1371/journal.pmed.1002790)
Supplement: S9 Table — Mann–Whitney test (AUC medians). (PDF) [file pmed.1002790.s014.pdf]

S9 Table. Whole blood intracellular cytokines statistical analysis: Area Under the Curve (AUC). 85A Mann-Whitney test (AUC medians)

**CD4 IFNγ**		
**Groups**	**P-value**	**95% CI**
1 vs 2	0.6016	-15.25 - 14.49
2 vs 3	0.8078	-15.75 - 8.901
1 vs 3	0.8874	-18.55 - 11.97
**CD4 TNFα**		
1 vs 2	>0.9999	-30.43 - 21.41
2 vs 3	0.8078	-23.45 - 12.26
1 vs 3	0.6707	-30.39 - 10.9
**CD4 IL-2**		
1 vs 2	0.8619	-2.847 - 7.213
2 vs 3	0.3723	-4.16 - 6.42
1 vs 3	0.1978	-0.898 - 6.189
**CD4 IL-17**		
1 vs 2	0.6505	-3.109 - 2.529
2 vs 3	0.3093	-2.061 - 4.878
1 vs 3	0.524	-2.115 - 4.03
**CD8 IFNγ**		
1 vs 2	0.1106	-4.495 - 0.3738
2 vs 3	0.5885	-0.8663 - 2.546
1 vs 3	0.2652	-4.112 - 1.038
**CD8 TNFα**		
1 vs 2	0.0673	-17.62 - 1.583
2 vs 3	0.8074	-4.136 - 12.81
1 vs 3	0.2189	-15.22 - 4.168
