# Supplementary material for: Alternate aerosol and systemic immunisation with a recombinant viral vector for tuberculosis, MVA85A: A phase I randomised controlled trial
Source: PLoS Med. 2019 Apr 30;16(4):e1002790. doi: 10.1371/journal.pmed.1002790 (PMC6490884; doi:10.1371/journal.pmed.1002790)
Supplement: S10 Table — Mann–Whitney test (AUC medians). (PDF) [file pmed.1002790.s015.pdf]

S10 Table. Whole blood intracellular cytokines statistical analysis: Area Under the Curve (AUC). MVA Mann-Whitney test (AUC medians)

**CD4 IFNγ**		
**Groups**	**P-value**	**95% CI**
1 vs 2	0.3186	-0.6062 - 7.496
2 vs 3	0.9333	-5.67 - 3.093
1 vs 3	0.1639	-0.264 - 3.536
**CD8 IFNγ**		
1 vs 2	0.2188	-8.2 - 13.08
2 vs 3	0.0118	-22.12 - -2.306
1 vs 3	0.6011	-15.82 - 1.864
**CD4 TNFα**		
1 vs 2	0.6016	-4.797 - 31.08
2 vs 3	0.433	-30.98 - 4.658
1 vs 3	0.7987	-4.871 - 6.541
**CD8 TNFα**		
1 vs 2	0.3443	-22.58 - 8.807
2 vs 3	0.6871	-10.37 - 13.25
1 vs 3	0.4428	-22.53 - 5.09
