# Supplementary material for: Alternate aerosol and systemic immunisation with a recombinant viral vector for tuberculosis, MVA85A: A phase I randomised controlled trial
Source: PLoS Med. 2019 Apr 30;16(4):e1002790. doi: 10.1371/journal.pmed.1002790 (PMC6490884; doi:10.1371/journal.pmed.1002790)
Supplement: S11 Table — (PDF) [file pmed.1002790.s016.pdf]

S11 Table. Ag85A- and MVA-specific serum IgA response for Groups 1, 2 and 3

Group 1

85A							MVA						
Day 0	Day 7	Day 14	Day 28	Day 35	Day 42	Day 168	Day 0	Day 7	Day 14	Day 28	Day 35	Day 42	Day 168
0.060	0.052	0.063	0.051	0.057	0.031	0.040	0.214	0.220	0.166	0.138	0.530	1.517	0.225
0.057	0.038	0.042	0.034	0.044	0.029	0.030	1.154	1.189	1.243	1.318	1.605	1.525	0.839
0.024	0.041	0.041	0.055	0.057	0.053	0.048	0.188	0.157	0.127	0.129	0.192	0.999	0.425
0.057	0.072	0.059	0.072	0.074	0.066	0.053	0.156	0.147	0.151	0.155	0.391	0.546	0.160
0.087	0.046	0.038	0.036	0.053	0.056	0.044	0.120	0.102	0.118	0.103	1.695	0.898	0.213
0.014	0.016	0.030	0.018	0.033	0.019	0.045	0.241	0.218	0.228	0.222	0.650	1.692	0.760
0.000	0.006	0.015	0.010	0.010	0.011	0.019	0.031	0.034	0.034	0.028	0.050	0.065	0.045
0.035	0.032	0.027	0.043	0.063	0.051	0.028	0.063	0.091	0.071	0.067	0.136	0.397	0.156
0.031	0.028	0.028	0.027	0.038	0.031	0.030	0.444	0.441	0.396	0.396	1.284	0.969	0.455
0.061	0.062	0.053	0.053	0.059	0.068	0.046	0.316	0.265	0.275	0.280	0.497	0.857	0.247
0.082	0.066	0.072	0.067	0.131	0.082	0.069	0.134	0.123	0.143	0.130	0.256	0.730	0.324
0.108	0.075	0.078	0.097	0.107	0.095	0.091	0.227	0.210	0.200	0.236	0.453	0.766	0.248

Group 2

85A							MVA						
Day 0	Day 7	Day 14	Day 28	Day 35	Day 42	Day 168	Day 0	Day 7	Day 14	Day 28	Day 35	Day 42	Day 168
0.053	0.045	0.051	0.052	0.053	0.041	0.043	0.130	0.234	1.044	0.459	0.355	0.668	0.819
0.053	0.061	0.060	0.062	0.073	0.060	0.068	0.230	0.488	0.920	0.226	0.219	0.265	0.306
0.052	0.048	0.057	0.045	0.042	0.042	0.046	0.105	0.585	0.645	0.160	0.153	0.142	0.142
0.033	0.030	0.022	0.026	0.039	0.056	0.053	0.193	0.247	0.502	0.175	0.151	0.365	0.358
0.506	0.392	0.374	0.355	0.450	0.449	1.026	0.123	0.328	0.691	0.303	0.277	0.340	0.678
0.096	0.105	0.092	0.090	0.128	0.107	0.105	0.133	0.187	0.613	0.259	0.206	0.269	0.194
0.086	0.092	0.173	0.122	0.120	0.114	0.116	0.245	0.398	1.461	0.555	0.527	0.369	0.322
0.109	0.128	0.159	0.158	0.145	0.145	0.127	0.224	0.414	0.882	0.506	0.549	0.560	0.438
0.023	0.037	0.034	0.036	0.039	0.041	0.026	0.080	0.116	0.576	0.233	0.205	0.205	0.158

Group 3

85A							MVA						
Day 0	Day 7	Day 14	Day 28	Day 35	Day 42	Day 168	Day 0	Day 7	Day 14	Day 28	Day 35	Day 42	Day 168
0.045	0.037	0.026	0.024	0.032	0.036	0.033	0.070	0.263	0.906	0.374	0.393	0.278	0.333
0.085	0.086	0.092	0.098	0.131	0.092	0.096	0.447	0.469	0.926	0.594	0.540	0.419	0.387
0.133	0.129	0.136	0.121	0.145	0.122	0.166	0.162	0.245	0.347	0.169	0.168	0.151	0.231
0.015	0.027	0.031	0.035	0.040	0.035	0.076	0.078	0.244	1.185	0.494	0.437	0.500	0.207
0.049	0.027	0.049	0.071	0.065	0.074	0.040	0.507	0.540	1.584	0.704	0.641	0.727	0.703
0.084	0.096	0.092	0.091	0.098	0.089	0.065	0.263	0.379	0.716	0.250	0.283	0.258	0.266
0.091	0.114	0.149	0.096	0.080	0.056	0.042	0.165	0.483	1.176	0.459	0.431	0.282	0.245
0.028	0.024	0.032	0.028	0.042	0.040	0.039	0.065	0.225	0.869	0.350	0.334	0.334	0.297
0.069	0.046	0.053	0.044	0.047	0.051	0.051	0.216	0.343	1.241	0.525	0.501	0.418	0.372
0.039	0.052	0.048	0.049	0.060	0.055	0.051	0.293	0.421	2.295	0.709	0.805	0.752	0.440
0.133	0.155	0.120	0.119	0.136	0.123	0.136	0.403	0.467	1.563	0.788	0.913	0.753	0.404
0.116	0.078	0.064	0.067	0.063	0.073	0.073	0.301	0.517	0.844	0.557	0.678	0.749	0.467
